# Oral Chinese Herbal Medicine plus usual care for diabetic kidney disease: study protocol for a randomized, double-blind, placebo-controlled pilot trial

**DOI:** 10.3389/fendo.2024.1334609

**Published:** 2024-02-08

**Authors:** Meifang Liu, Yuan Ming Di, Lei Zhang, Lihong Yang, La Zhang, Junhui Chen, Ruobing Wang, Xiaoning Xie, Fang Lan, Liping Xie, Juan Huang, Anthony Lin Zhang, Charlie Changli Xue, Xusheng Liu

**Affiliations:** ^1^ The China-Australia International Research Centre for Chinese Medicine, School of Health and Biomedical Sciences, RMIT University, Melbourne, VIC, Australia; ^2^ State Key Laboratory of Dampness Syndrome of Chinese Medicine, The Second Affiliated Hospital of Guangzhou University of Chinese Medicine, Guangzhou, China; ^3^ Department of Nephrology, Guangdong Provincial Hospital of Chinese Medicine, The Second Affiliated Hospital of Guangzhou University of Chinese Medicine, Guangzhou, China; ^4^ Evidence-Based Medicine and Clinical Research Service Group, Guangdong Provincial Hospital of Chinese Medicine, The Second Affiliated Hospital of Guangzhou University of Chinese Medicine, Guangzhou, China; ^5^ The Second Clinical College of Guangzhou University of Chinese Medicine, Guangzhou, China; ^6^ Department of Nephrology, The First Affiliated Hospital of Guangxi University of Chinese Medicine, Nanning, China; ^7^ Pharmaceutical Research Team for New Drug Development and Authentication of Chinese Medicines, Guangdong Provincial Hospital of Chinese Medicine, The Second Affiliated Hospital of Guangzhou University of Chinese Medicine, Guangzhou, China

**Keywords:** Chinese herbal medicine, *Tangshen Qushi Formula*, diabetic kidney disease, randomized controlled trial, semi-structured interview, feasibility, efficacy, safety

## Abstract

**Background:**

Diabetic kidney disease (DKD) has become the leading cause of kidney failure, causing a significant socioeconomic burden worldwide. The usual care for DKD fails to achieve satisfactory effects in delaying the persistent loss of renal function. A Chinese herbal medicine, *Tangshen Qushi Formula* (TQF), showed preliminary clinical benefits with a sound safety profile for people with stage 2-4 DKD. We present the protocol of an ongoing clinical trial investigating the feasibility, efficacy, and safety of TQF compared to placebo in delaying the progressive decline of renal function for people with stage 2-4 DKD.

**Methods:**

A mixed methods research design will be used in this study. A randomized, double-blind, placebo-controlled pilot trial will evaluate the feasibility, efficacy, and safety of TQF compared to placebo on kidney function for people with stage 2-4 DKD. An embedded semi-structured interview will explore the acceptability of TQF granules and trial procedures from the participant’s perspective. Sixty eligible participants with stage 2-4 DKD will be randomly allocated to the treatment group (TQF plus usual care) or the control group (TQF placebo plus usual care) at a 1:1 ratio for 48-week treatment and 12-week follow-up. Participants will be assessed every 12 weeks. The feasibility will be assessed as the primary outcome. The changes in the estimated glomerular filtration rate, urinary protein/albumin, renal function, glycemic and lipid markers, renal composite endpoint events, and dampness syndrome of Chinese medicine will be assessed as the efficacy outcomes. Safety outcomes such as liver function, serum potassium, and adverse events will also be evaluated. The data and safety monitoring board will be responsible for the participants’ benefits, the data’s credibility, and the results’ validity. The intent-to-treat and per-protocol analysis will be performed as the primary statistical strategy.

**Discussion:**

Conducting a rigorously designed pilot trial will be a significant step toward establishing the feasibility and acceptability of TQF and trial design. The study will also provide critical information for future full-scale trial design to further generate new evidence supporting clinical practice for people with stage 2-4 DKD.

**Trial registration number:**

https://www.chictr.org.cn/, identifier ChiCTR2200062786.

## 1 Introduction

Diabetic kidney disease (DKD) is progressive kidney damage caused by diabetes with changes in urinary protein and renal function (1). DKD has become the leading cause of end-stage kidney disease (ESKD) worldwide over the past three decades (2). Increased urinary albumin excretion and progressive reduced estimated glomerular filtration rate (eGFR) are the main clinical manifestations of DKD, both proven to be risk factors for ESKD, cardiovascular events, and death (3). Kidney Disease: Improving Global Outcomes (KDIGO) guideline recommends a five-stage category for DKD based on albuminuria and GFR (4). People with stage 2-4 DKD are considered the most representative populations, as stage one is normal, and stage 5 is at high risk of cardiovascular disease and death (5). Stage 2-4 DKD could progress fast and eventually develop into ESKD without proper interventions (6).

Clinical guidelines and expert consensus have been published regarding new drugs or strategies for the comprehensive management of DKD (4, 7–9). The first-line pharmacotherapy recommended for DKD in the clinical guidelines includes sodium-glucose cotransporter 2 (SGLT2) inhibitor, metformin, renin-angiotensin-system (RAS) blockade, and moderate- or high-intensity statin. These therapeutic agents can effectively reduce urinary albumin and the risk of composite renal outcomes for people with non-dialysis DKD (10–13). However, side effects of the recommended therapy were observed in clinical trials and practice, including hypoglycemia, diabetic ketoacidosis, urinary tract infection, and genital mycotic infections because of taking SGLT2 inhibitors (14). Further, there is a lack of evidence supporting the efficacy and safety in delaying the progress of eGFR decline, especially for people with stage 2-4 DKD. Given the lack of effective pharmacotherapy to slow the persistent loss of renal function, alternative and adjuvant management is urgently needed to address this clinical gap.

Chinese herbal medicine (CHM) plus usual care has demonstrated effects in protecting renal function and decreasing albuminuria, which is superior to that shown by usual care alone for DKD patients in previous clinical trials (15, 16). Fewer side effects are reported when using CHM with usual care (17). *Tangshen Qushi Formula* (TQF) is a CHM derived from the classical Chinese formula *Ping Wei San* recorded in *Welfare Pharmacy* (*Shiwen Chen*, 1151AD) and modified by specialists in modern clinical practice, showing the function of tonifying the Kidney and dispelling dampness in the Chinese medicine theory. TQF consists of seven herbs, namely *Astragalus mongholicus Bunge* [*Fabaceae*; *Astragali mongholici radix*], *Cuscuta australis R.Br.* [*Convolvulaceae*; *Cuscutae semen*], *Prunus davidiana (Carrière) Franch.* [*Rosaceae*; *Persicae semen*], *Atractylodes lancea (Thunb.) DC.* [*Asteraceae*; *Atractylodis lanceae rhizoma*], *Citrus × aurantium L.* [*Rutaceae*; *Aurantii amari epicarpium et mesocarpium*], *Centella asiatica (L.) Urb.* [*Apiaceae*; *Centellae asiaticae herba*], and *Isaria cicadae Miquel* [*Cordycipitaceae*; *Isaria cicadae Miquel*]. The dose of each herb in TQF is 20: 15: 10: 10: 10: 15: 8 (gram), respectively, aligning with the Chinese Pharmacopeial (2020 Edition) and clinical experience.

Preclinical evidence indicates that the phytochemicals from the herbs contained in TQF, such as astragaloside IV, calycosin, and hesperetin, could help attenuate pathology in DKD animals and cell lines. Astragaloside IV from *Astragali Radix* is found to have the reno-protective function of alleviating renal tubular epithelial-mesenchymal transition via CX3CL1-RAF/MEK/ERK signal pathway in DKD mice (18). Calycosin, another compound from *Astragali Radix*, shows a protective effect on DKD by regulating ferroptosis in cell lines and mice (19). Hesperetin, a flavanone glycoside compound of *Citrus × aurantium L.*, produced renal benefits by activating Nrf2/ARE/glyoxalase one and TGFβ1- ILK-Akt, thus ameliorating DKD in animal models (20, 21). Additionally, evidence from clinical research showed that *Astragalus mongholicus Bunge* plus conventional treatment could reduce urinary albumin or protein excretion and lower serum creatinine levels for DKD patients in the short term (22).

The safety and toxicity of TQF in animals were assessed on db/m mice before using it on DKD patients. We found no impairments by observing changes in serum biochemistry and kidney histopathology in db/m mice, indicating that TQF did not cause harm and was well-tolerated (unpublished data). Further, an open-label single-arm trial was conducted to explore the efficacy and safety of TQF for people with stage 2-4 DKD. We found that TQF plus usual care delayed the mean decline of eGFR by -2.61 ± 2.58 mL/min/1.73m^2^, decreased the level of HbA1c, and improved high-density lipoprotein-cholesterol (23). No adverse events were reported. These findings indicated that TQF could delay the progression of DKD with a sound safety profile. However, an appropriate control should be used to further validate the safety and efficacy of TQF in DKD patients. Therefore, we have designed a mixed methods study to evaluate the feasibility of a rigorously designed clinical trial to assess the efficacy and safety of TQF compared to placebo on kidney function for people with stage 2-4 DKD. This study also aims to seek an in-depth understanding of the acceptability of TQF granules and trial procedures by investigating participants’ expectations, motivations, experiences, and compliance with participating in this pilot trial.

## 2 Methods and analysis

### 2.1 Study design

A mixed methods design has been adopted in this study, encompassing qualitative and quantitative components. The quantitative part is a two-center, parallel, add-on, randomized, double-blind, placebo-controlled pilot trial, followed by semi-structured interviews with trial participants. The study protocol has been registered with the Chinese Clinical Trial Registry (No. ChiCTR2200062786), one of the International Clinical Trial Registration Platforms of the World Health Organization.

The design of the pilot trial is in line with the Standard Protocol Items: Recommendations for Interventional Trials (SPIRIT) 2013 Statement (24) (**Supplementary File 1**) and Consolidated Standards of Reporting Trials (CONSORT) 2010 statement: extension to randomized pilot and feasibility trials (25), while the interview part will comply with the Consolidated Criteria for Reporting Qualitative Research (COREQ): A 32-Item Checklist for Interviews and Focus Groups (26).

**Figure 1:**
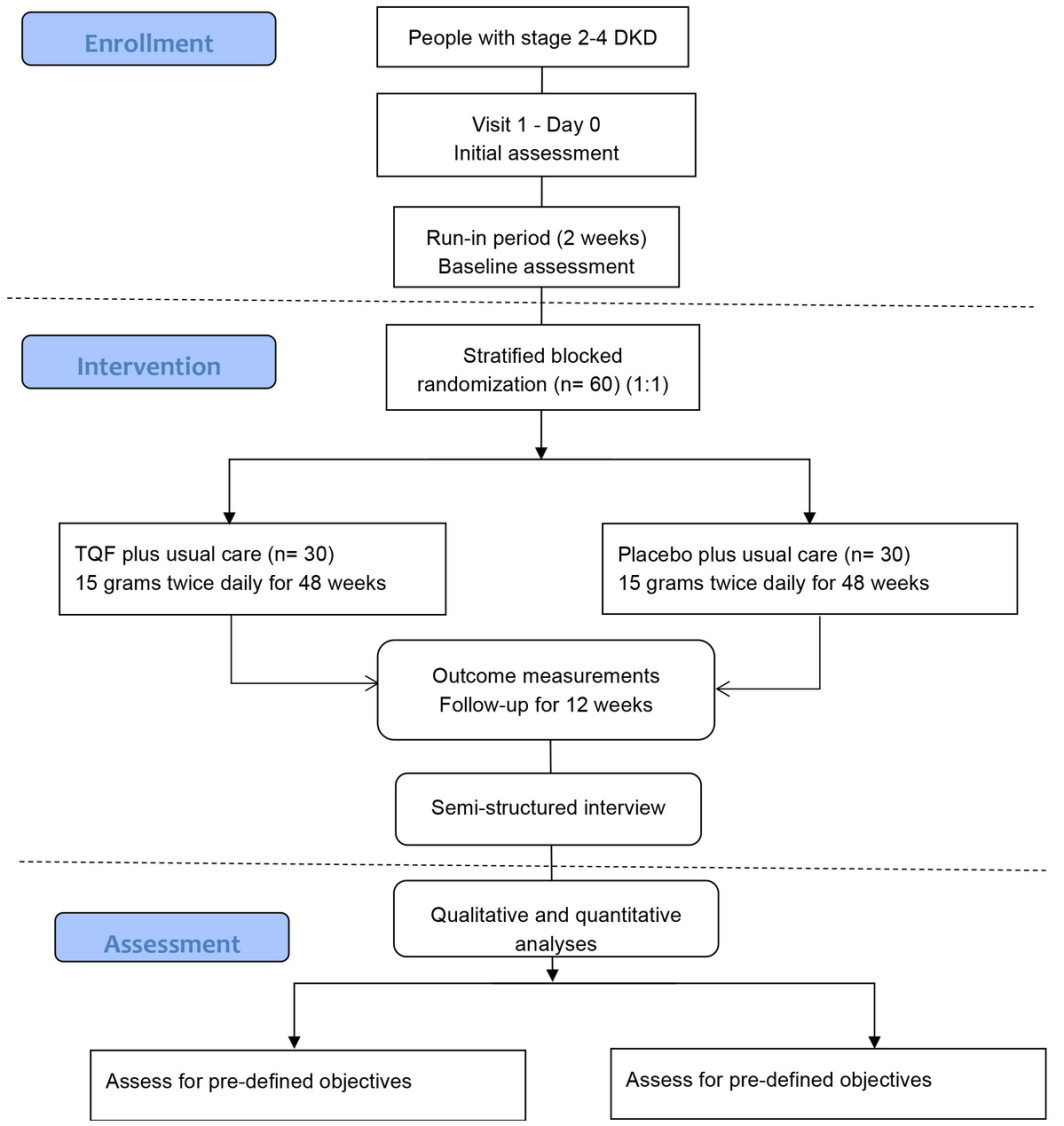
Study flow diagram. DKD, diabetic kidney disease; TQF, *Tangshen Qushi Formula*.

Sixty participants will be enrolled in the trial for 62 weeks, consisting of two weeks of run-in, 48 weeks of treatment, and 12 weeks of follow-up. The SPIRIT schedule of enrolment, interventions, and assessments and the trial flow diagram are shown in **Table 1** and **Figure 1**, respectively.

**Table 1:** SPIRIT schedule of enrolment, interventions, and assessments.

Study period		Screening	Enrolment	Allocation	Intervention				Follow-up
**Timepoint**		-t1	***0***	t1	t2	t3	t4	t5	t6
		-2 w	0 d	2 w	14 w	26 w	38 w	50 w	62 w
**Enrollment**	Initial screening	X							
	Informed consent		X						
	Eligibility screening		X						
	Run-in		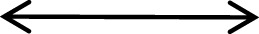						
	Randomization & allocation			X					
**Interventions**	TQF plus usual care			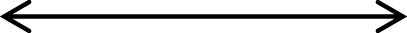					
	Placebo plus usual care			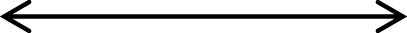					
**Assessments: Research activities**	General information questionnaire		X						
	Physical examination		X	X	X	X	X	X	X
	Case report form		X	X	X	X	X	X	X
	A 30-day medication diary				X	X	X	X	
	Medication compliance check				X	X	X	X	
	Credibility of blinding							X	
**Assessments: Efficacy outcomes**	eGFR slope			X	X	X	X	X	X
	Urine protein/urine creatinine			X	X	X	X	X	X
	Urine albumin/urine creatinine			X	X	X	X	X	X
	SCr, BUN, UA, TCO2			X	X	X	X	X	X
	Hemoglobin A1c			X	X	X	X	X	X
	*Fasting blood glucose*			X	X	X	X	X	X
	TG, TC, LDL-C, HDL-C			X	X	X	X	X	X
	Composite renal endpoint events			X	X	X	X	X	X
	A score of the dampness syndrome scale of chinese medicine								
**Assessments: Safety outcomes**	ALT, AST, ALB, GLB			X	X	X	X	X	X
	Serum potassium/phosphorus/calcium			X	X	X	X	X	X
	Blood/urine routine test			X	X	X	X	X	X
	Fecal routine and occult blood test			X	X	X	X	X	X
	Electrocardiograph			X				X	
	Urinary color doppler ultrasound			X				X	
	AEs/SAEs				X	X	X	X	
**Qualitative study**	Semi-structured interviews								X

TQF, Tangshen Qushi Formula; GFR, glomerular filtration rate; SCr, serum creatinine; BUN, blood urea nitrogen; UA, uric acid; TCO2, total carbon dioxide; TG, triglyceride; TC, total cholesterol; LDL-C, low-density lipoprotein cholesterol; HDL-C, high-density lipoprotein cholesterol; ALT, alanine transaminase; AST, aspartate transaminase; ALB, albumin; GLB, globulin; AE, adverse event; SAE, serious adverse event; -t_1_: time point before day 0, t_1_: time point 1, t_2_: time point 2, and so on.

### 2.2 Chemical analysis

A chemical profile following the standards established in the ConPhyMP statement is essential for a clinical trial using a polyherbal preparation (27). We have conducted a chemical analysis of TQF granules following the standards via ultra-high performance liquid chromatography-mass spectrometry/mass spectrometry (UHPLC-MS/MS). The chemicals contained in TQF were assessed by high-performance liquid chromatography spectrometry (HPLC). Detailed information is provided in the **Supplementary File** (**Supplementary File 2**).

**Figure 2:**
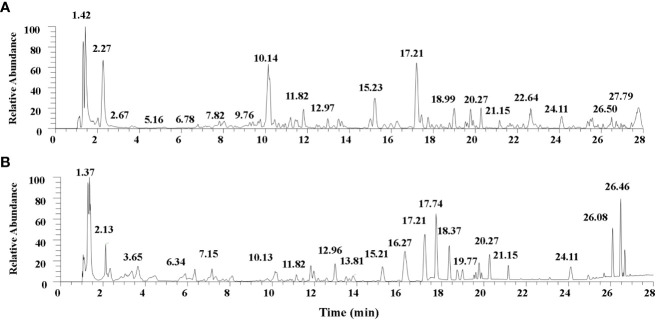
The base peak chromatogram of *Tangshen Qushi Formula* granules in negative mode **(A)**/positive mode **(B)**.

Thirty-eight compounds were identified from UHPLC-MS/MS analysis of TQF. The details of the compounds and their predominant bioactivities are listed in the **Supplementary Table S1**. The base peak chromatogram of TQF granule in negative mode and positive mode detail are shown in **Figure 2**.

**Figure 3:**
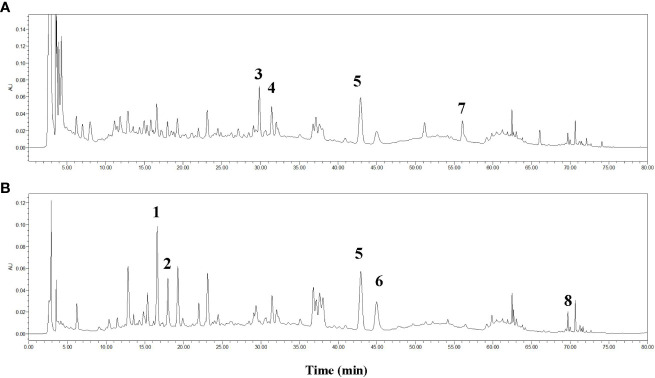
HPLC fingerprint for *Tangshen Qushi Formula* granules at 254 nm **(A)** and 340 nm **(B)**. 1: Chlorogenic acid, 2: Cryptochlorogenic acid, 3:Calycosin 7-O-glucoside, 4: Hyperoside, 5: Hesperidin, 6: Isochlorogenic acid C, 7: Calycosin, 8: Nobiletin.

The chemicals contained in TQF were assessed by HPLC. As compared with standard reference compounds, eight constituents of TQF granules were identified, including chlorogenic acid, cryptochlorogenic acid, calycosin 7-O-glucoside, hyperoside, hesperidin, chlorogenic acid C, calycosin, nobiletin (**Figure 3**).

### 2.3 Ethics and dissemination

Ethics approval has been obtained from the ethics committee of Guangdong Provincial Hospital of Chinese Medicine (GPHCM) (No. YF2022-152-01) and Guangxi Hospital of Chinese Medicine (GXHCM) (No. LS2022-021-02) (**Supplementary File 3**). The ethics approval is also filed with the RMIT ethics committee (No. 2022-24963-18150). Written informed consent will be obtained from the eligible participants before the baseline assessment and data collection. This study will follow the *Declaration of Helsinki, Guideline for Good Clinical Practice ICH E6 (R2) ICH Consensus Guideline* and *Measures for the Ethical Review of Biomedical Research Involving Humans (2016, in Chinese)*. Medical records and biological samples may be used again in future similar studies. In this case, ethics approval will be obtained before the release of the materials. The results from this study will be published in peer-reviewed medical journals, Ph.D. thesis, and/or scientific conferences. However, any information obtained from this study that can identify an individual will remain confidential and only be used for this study. A one-page summary of the results will be provided to study participants.

### 2.4 Participants and recruitment

The study is being conducted in the outpatient nephrology department of two hospitals in China, Guangdong Provincial Hospital of Chinese Medicine and Guangxi Hospital of Chinese Medicine.

#### 2.4.1 Inclusion criteria

Participants who are:

1) Aged 18 years and over;
2) Diagnosed with DKD using specified diagnostic criteria (see below);
3) 15 mL/min/1.73m^2^ ≤ eGFR < 90 mL/min/1.73m^2^ and the change of eGFR within the latest 12 weeks less than 30%;
4) Urine protein creatinine ratio (UPCR) ≤ 3500 mg/g;
5) Agree to be available for the period of the study;
6) Providing written informed consent.

American Diabetes Association recommends that DKD is diagnosed based on a clear history of diabetes (28) and a causal relationship with changes in urinary protein and renal function after excluding other primary glomerular diseases and systemic diseases. Besides, the diagnosis of DKD should meet at least one of the following criteria based on the diagnostic guideline (29):

1) Urine albumin creatinine ratio (UACR) ≥ 30 mg/g or urinary albumin excretion rate ≥ 30 mg/24h, repeated within 3 to 6 months, and 2 out of 3 times reached or exceeded the critical value after eliminating infection and other interfering factors;
2) eGFR < 60 mL/min/1.73 m^2^;
3) The renal biopsy is consistent with DKD pathological changes.

Diagnosis of kidney deficiency and dampness syndrome in Chinese medicine is based on the expert consensus of the syndrome differentiation at different stages and efficacy evaluation proposal for DKD (30). The objective physiological characteristics will be used to diagnose kidney deficiency and dampness syndromes.

#### 2.4.2 Exclusion criteria

Participants having one or more of the following conditions before enrolment will be excluded:

1) Received maintenance dialysis treatment or kidney transplantation;
2) Planning to accept maintenance dialysis treatment or kidney transplantation within two weeks;
3) Diagnosed with acute urinary tract infection in the last two weeks;
4) Diagnosed with other primary chronic kidney diseases (e.g., glomerulonephritis, chronic pyelonephritis, and ischemic kidney disease);
5) Diagnosed with advanced hepatic disease (e.g., hepatic cirrhosis, decompensated cirrhosis) or other known acute and chronic active hepatitis, cirrhosis;
6) Diagnosed with heart failure (New York Heart Association grade III-IV (31)), acute coronary syndrome, stroke, or any other cardiovascular or cerebrovascular accident requiring revascularization surgery in the last 12 weeks, or a revascularization operation is urgently needed;
7) Diagnosed with severe acute diabetic complications include but are not limited to ketoacidosis, lactic acidosis, hypertonic non-ketotic diabetic coma, hypoglycemic coma occurring within 24 weeks;
8) Diagnosed with active malignant tumor disease within 5 years;
9) Diagnosed with mental illness or language barrier and are unable or unwilling to cooperate;
10) Diagnosed with autoimmune diseases (for example, systemic lupus erythematosus) and are receiving hormone or immunosuppressive therapy;
11) Allergic to any medications or ingredients or intolerance to these medications or ingredients (e.g., lactose intolerance);
12) Participating in another clinical trial;
13) Female during pregnancy or lactation;
14) Other conditions that the investigator judged inappropriate for the study.

#### 2.4.3 Recruitment

The participants will be recruited from the outpatient nephrology department of GPHCM and GXHCM. This study will be advertised through e-posters, leaflets, and hospital bulletin boards. Contact with the participants will be at the trial clinic at GPHCM and GXHCM. Initial contact with potential participants will be face-to-face in the outpatient clinic. Investigators will also conduct an initial screening of the electronic medical records from the Hospital Information System to identify potential participants. Eligible participants will be provided with the Recruitment Advertisement, Plain Language Statement, Patient Information and Consent Form for this trial. Potential participants will then be contacted to schedule an initial assessment. Participants will attend the trial clinic at the participating hospital to assess their eligibility. A Screening Questionnaire will be used to assess the specific inclusion and exclusion criteria. Informed consent will be sought when the participants are eligible and willing to participate. Participants’ demographic information will be collected using a General Information Questionnaire. All the mentioned documents are provided in **Supplementary File 4** (Appendices 1 to 14).

### 2.5 Sample size estimation

Whitehead et al. suggested that the estimated sample size of randomized controlled pilot trials with 90% power and 2-tailed 5% significance should be 75, 25, 15, and 10 for each treatment arm with the standardized effect sizes of extra small (0.1), small (0.2), medium (0.5), or large (0.8), respectively (25, 32). The primary outcome eGFR slope is used to estimate the sample size in our pilot trial. The exact effect size is calculated by the minimum clinically important difference (MCID) of the eGFR slope and the mean value of the control group. The mean value of 3.79 of the placebo-control group in the DAPA-CKD trial (13) is selected for the estimation. The MCID of the eGFR slope is 0.75 mL/min/1.73m^2^ based on the evidence from randomized controlled trials and observational studies with large enough sample sizes (33, 34). Given that a change in 0.75 is the MCID from the mean value of 3.79, a 20% difference would be clinically significant. The 20% change (0.2) is used as our effect size. Thus, we will recruit 25 participants for each treatment arm accordingly. With an approximate 15% attrition rate based on our previous clinical study (23), we plan to recruit 60 participants, with 30 participants for each arm in this study.

### 2.6 Randomization

After a two-week run-in period, participants who meet the inclusion criteria will be randomly assigned to either the TQF group or the TQF placebo group at a 1:1 ratio. The personnel from the Key Unit of Methodology in Clinical Research (KUMCR) at GPHCM conducted the randomization. SAS 9.3 (SAS Institute, Cary, NC, USA) software will generate random allocation sequences by stratified block methods. To ensure allocation concealment, the randomization numbers are delivered to study sites via a validated web-based randomization system, Interactive Web Response System for Chinese Medicine Trials. Every participant will be assigned a random number, and a comprehensive record of all procedures will be documented and preserved.

### 2.7 Blinding

The group allocation and intervention will be concealed from all participants, investigators, personnel involved in drug delivery, outcome measurements, and statisticians from the time of allocation until the completion of data analysis. The research drugs will be delivered according to the randomization numbers. Besides, the randomization list and blinding codes will be kept confidential by KUMCR staff. Only authorized personnel have the right to access the information for the duration of the study.

The credibility of the blinding procedure will be assessed by asking the participants which treatment they believe they have received at the end of treatment.

### 2.8 Interventions

#### 2.8.1 Usual care

Participants in both groups will receive usual care according to the KDIGO and Chinese guidelines for DKD (4, 9), including lifestyle management, glycemic control, antihypertensive therapy, control of proteinuria, lipid-lowering therapy, and uric acid control. The usual care will be balanced between groups.

#### 2.8.2 TQF and placebo

The compositions of TQF herbs and their scientific name, species name, pharmaceutical name, Chinese Pin Yin name, proportion, and place of origin are provided in **Supplementary Table S2**. The TQF herbs were subject to hot water extraction, concentration, spray-drying, granule formation, and sealed packaging within opaque sachets. The production has been performed by Jiangyin Tianjiang Pharmaceutical Company (Jiangyin, Jiangsu, China) strictly adhering to good manufacturing practice (GMP) standards. In parallel, the TQF placebo underwent production by the same manufacturer using a blend of food-grade colorings of caramel, gardenia yellow and sunset yellow, sucrose octaacetate, and dextrin. The TQF placebo is similar in color, smell, taste, appearance, and packaging to the TQF granule. Qualities of the TQF placebo, such as appearance, determination of water, size of granule, solubility, hygroscopicity, heavy metals, toxic elements, pesticide residues, and microbial contamination, are also rigorously controlled by adhering to the stipulations outlined in the 2020 Chinese pharmacopeia.

#### 2.8.3 Intervention

TQF or placebo medications will be given to the participants by a registered pharmacist according to the randomized numbers. TQF or TQF placebo granules of 15 grams will be taken orally twice daily by dissolving in boiled water. The participants will take the granules for 48 weeks. A 30-day Medication Diary will be delivered to the participants every 4 weeks. The packets of taken granules and left-over medications will be collected and recorded in the Compliance Check Form. Participants allocated to the placebo group can take the TQF granule for 12 weeks at no cost after the follow-up period. A valid and reproducible Dampness Syndrome Scale of Chinese Medicine, which strictly follows the international standard for scale development, will be used to measure the changes in dampness syndrome (35).

Chinese herbal formulas or Chinese patent medicine that have the function of tonifying the Kidney and dispelling dampness are prohibited in this trial to avoid contamination of the intervention. Those formulas and patent medicine include but are not limited to *Huangkui* Capsule, *Yishen Huazhuo* Granule, *Yishen Huashi* Granule, *Niao Du Qing* Granule, and *Niao Du Kang* Granule. The participant who has taken these drugs for more than one week will be withdrawn from this study.

### 2.9 Outcomes

#### 2.9.1 Feasibility

The feasibility of TQF and trial design will be explored as the primary outcome. The key domains for the assessment include recruitment, intervention protocol, outcome assessments, and data collection methods. The eligibility, recruitment, retention, and completion rates will be investigated at the end of the pilot trial:

1) Eligibility rate: the percentage of eligible participants over the number of screened participants;
2) Recruitment rate: the percentage of recruited participants over eligible participants;
3) Retention rate: the percentages of participants in both groups at the end of treatment and end of follow-up over the number of participants randomized;
4) Completion rate: the percentage of participants who completed the treatment phase over the number of participants randomized.

Semi-structured interviews will be conducted to further explore the feasibility among the trial participants.

#### 2.9.2 Efficacy outcomes

The efficacy of TQF for people with stage 2-4 DKD will be assessed on renal-related outcomes every 12 weeks.

The eGFR slope will be measured at the end of the treatment. eGFR slope is a validated surrogate endpoint for clinical trials to indicate kidney disease progression (33, 34). The Chronic Kidney Disease Epidemiology Collaboration (CKD-EPI) equation will be used to calculate eGFR (36).

Other efficacy outcomes will be measured from baseline to the end of treatment: changes in UACR, UPCR, serum creatinine, blood urea nitrogen, uric acid, HbA1c, fasting blood glucose (FBG), and blood lipids (cholesterol, triglycerides, low-density lipoprotein cholesterol, and high-density lipoprotein cholesterol). The laboratory equipment for all the laboratory outcomes will be standardized and follow a consistent operating procedure. Additionally, the changes in the score of the Dampness Syndrome Scale of Chinese medicine and the survival time of renal composite endpoint events will also be evaluated (35). The survival time is defined as starting from baseline to the occurrence of any of the below composite endpoints (37): receipt of a kidney transplant, initiation of maintenance dialysis, death from kidney failure, a sustained low eGFR, and a sustained percent decline in eGFR.

#### 2.9.3 Safety outcomes

The blood samples will be collected and tested for safety every 12 weeks, including serum creatinine, potassium, phosphorus, calcium, alanine transaminase, aspartate transaminase, albumin, globulin, and full blood count (whole blood). Urine routine test, fecal routine, occult blood test, electrocardiograph, and urinary color Doppler ultrasound will be assessed at baseline and the end of treatment. Adverse events or severe adverse events will also be evaluated as safety outcomes.

### 2.10 Safety considerations

An adverse event (AE) is any unfavorable and unintended sign (including an abnormal laboratory finding), symptom, or disease temporally associated with TQF or TQF placebo that may or may not have a causal relationship with TQF or TQF placebo (38). Abnormal laboratory test results are considered adverse events only if they induce clinical signs or symptoms. Participants record any AE immediately in the 30-day medication diary or contact the researchers when they happen. The researchers will record the AE in the Adverse Event Form by referring to the 30-day Medication Diary.

A serious adverse event (SAE) or experience or reaction is any untoward medical occurrence that involves the potential to result in death, is life-threatening, permanently incapacitating, or results in hospitalization (39). If the SAE occurs, the principal investigator can apply for emergency unblinding when it is essential for further management of the participants. The details of SAE will be recorded in the Serious Adverse Event Form by researchers and submitted to the ethics committee at GPHCM within 7 days of the first knowledge of the SAE. Participants are suggested to seek medical support from the nearest hospital’s emergency department as necessary.

A Data and Safety Monitoring Board (DSMB) has been established by external experts in pharmaceutical research, nephrology, statistics, and ethics. The DSMB will hold regular meetings every three months during the trial to provide advice on the overall study progress and safety data. One research assistant will prepare a summary of interim, unblinded, comparative data only for the DSMB members at the regular meetings. The DSMB will evaluate and decide whether to continue, modify, or terminate the trial. The researchers will report SAEs immediately to DSMB and take appropriate treatment measures if this occurs. This way, participants’ benefits can be safeguarded, and credible and valid results can be produced.

### 2.11 Withdrawal and termination

Participants may voluntarily withdraw from the study for any reason at any time. Participants will be considered withdrawn if they state an intention to withdraw, reach composite renal endpoint events, or become lost to follow-up for any other reason. When withdrawal occurs, the researcher must determine the primary reason for premature withdrawal from the study and record this information on the Case Report Form (CRF). The research team and study doctor may remove a participant from further participation in this study if (1) staying in the study would be unsafe or harmful to the participant; (2) the participant needs treatment that is not allowed in this study; (3) participant fails to follow instructions; (4) participant becomes pregnant. This study may be stopped for several reasons, including unacceptable side effects, the intervention being shown to have no effect, or being effective with no need for further investigation.

### 2.12 Semi-structured interviews

Individual, semi-structured, in-depth interviews will be carried out with volunteer participants at the end of the trial. The nested semi-structured interview aims to address the following research questions: 1) what expectations, motivations, experiences, and compliance do participants associate with their involvement in the trial, and how do these factors influence their engagement? 2) what are the main challenges and barriers participants encountered while taking the TQF granules and adhering to the trial procedures, and how did they navigate them? 3) what are the feasibility and acceptability of trial procedures (including recruitment, intervention protocol, outcome assessments, and data collection methods) with participants?

The target population is participants from two groups of the pilot trial at Guangdong Provincial Hospital of Chinese Medicine. Eligible criteria: 1) participants who have completed the treatment and follow-up or dropped out but completed at least 12 weeks of treatment and one post-treatment assessment; 2) be able to read and speak Chinese (Mandarin); 3) agree to provide informed consent (including the radio recording). The participants will be selected by purposeful and convenience sampling strategies. The purposeful sampling is to select the participant who can provide the maximum amount of information regarding age, gender, disease stages, medical history, and the trial’s preliminary results. Convenience sampling means choosing the most straightforward examples to save participants and researchers money, time, and effort. It is anticipated that 16 to 24 participants from the trial will be recruited for the qualitative interview (40). Data saturation will be obtained when no new information is discovered in the additional interviews. A separate consent will be provided before the commencement of the qualitative interview.

Interviews will be conducted in a separate room of study sites. Each interview will take about 30-60 minutes. Interviews are semi-structured and guided by an interview guide. A pilot interview will be conducted before the formal interview to test the interview guide. The interview will be conducted in Chinese (Mandarin or Cantonese) and recorded by audio/video. The non-verbal communication, such as facial expressions and body movements, will be taken down by another trained researcher. The interviewer can change the order of questions and details in the interview guide according to the specific situation in the interview with flexibility. Participants are encouraged to speak freely about their experiences and compliance in taking part in the trial. The topic of interest will cover the experiences and expectations of TQF intervention, randomization, recruitment, informed consent process, issues with treatment compliance, and appropriateness of data collection forms/questionnaires and outcome measurements.

### 2.13 Data collection and management

Data will be collected from CRF and related files by the researchers via the data entry software EpiData version 3.1. The data will be double-entered by two researchers and checked by another researcher. De-identified data will be entered into a secure computer database that will be used for this study only.

Data storage and security align with China’s Centre for Drug Evaluation guidelines (38). During the study, the blood, urine, and feces specimens from participants will be collected and stored according to the standard operating procedures for 10 years after the completion of the study, and the samples will be destroyed when the storage life ends. CRF collected in this study will be stored securely for 15 years after the completion of the study. Data will be stored in locked cabinets, and all electronic files will be stored in a password-protected, non-networked computer in a locked room at GPHCM. Access will only be by authorized personnel involved in the research. Data will not be stored on laptop computers or removed from the clinical trial site.

### 2.14 Statistical analysis

The quantitative data will be processed and analyzed by an independent statistician at GPHCM using PASW 18.0 (IBM SPSS Inc., Armonk, New York, USA) and Stata (Version 15.0, Stata Corp. College Station, TX, USA) with two-tailed 5% significance.

We will perform the primary analysis on the full analysis population following the Intent-to-Treat (ITT) principle. The full analysis population refers to randomized participants who received at least one dose of treatment and had baseline data along with at least one post-baseline efficacy assessment. We will also conduct a per-protocol analysis as a secondary analysis, including participants who completed the study without significant protocol violations. Safety analysis will be performed on the randomized participants who have received at least one dose of TQF or placebo.

Descriptive analysis will be performed on demographic and baseline characteristics. Categorical variables will be summarized as frequencies and percentages. The difference between the two groups will be assessed with a *chi*-squared test. Continuous variables will be reported as means with standard deviations for or medians with interquartile ranges. The difference between the two groups will be detected by Student’s *t*-test for normally distributed data, or the Mann-Whitney U-test for non-normally distributed variables.

The primary efficacy outcome eGFR slope will be assessed through a linear mixed-effects model in which treatment and the interaction of treatment and time are treated as a fixed effect while the time effect is a random effect using an unstructured covariance matrix. A secondary analysis for the eGFR slope will be conducted through the superiority test with a 95% confidence interval (CI), to explore any clinical disparities associated with TQF.

The secondary outcome time to composite renal endpoint events will be analyzed by the Kaplan-Meier method. The log-rank test will be employed to compare time to composite outcomes between the two groups, followed by Cox proportional hazards regression to estimate hazard ratios with 95% CIs. Other continuous outcomes will be performed by Student’s *t*-test or Mann-Whitney U-test, while categorical variables will be examined using the *chi*-square test or Mann-Whitney U-test. The Cochran-Mantel-Haenszel test will be used for categorical variables, adjusting for center-related effects. Subgroup analysis will consider risk factors including but not limited to age, gender, hypertension, and proteinuria. Additionally, the Fisher exact test will be utilized to compare the incidence rates of adverse events and adverse drug reactions throughout the treatment duration.

An ITT analysis will be applied using the imputation method for missing data. We expect less than 20% of missing data points. The linear trend at point method will be used to replace missing values. The existing series will be regressed on an index variable scaled from 1 to n, and the missing values will be replaced with their predicted values. If the predicted values cannot be computed, the last observation carried forward (LOCF) method will be used. We will perform a sensitivity analysis without imputation to assess the robustness of our results.

The semi-structured interviews will be transcribed verbatim. NVivo 11 software will be used to manage and code the data. Grounded theory will be used as a theoretical framework guiding the analysis of interview data, providing a systematic and rigorous approach to understanding complex phenomena within the clinical trial context (41). We will identify and conceptualize relevant themes on the participants’ experiences and expectations of TQF intervention and trial process. A content analysis will be used to analyze the interviews following six phases developed by Kuckartz (42). Firstly, we will get familiar with the original transcripts through repeated reading. Secondly, we establish an initial thematic framework from the keywords or recurring words mentioned by the interviewees in the transcripts. Thirdly, codes will be created for all information after establishing the initial theme framework in NVivo. Further, the content will be coded correspondingly through constant comparison. Next, themes will be defined and interpreted. Finally, we will complete reporting and documenting.

## 3 Discussion

Chinese herbal medicine (CHM) is the most studied and widely used therapy for DKD in China on top of usual care (43). CHM typically employs diverse preparation types, including decoctions, liquid extractions, powder capsules, pills, and granules. The evidence from the *in vivo* and *in vitro* studies of CHM for DKD indicates the effect in regulating metabolism disorders, reducing inflammatory and oxidative stress, anti-fibrosis, and protecting podocytes (44). The efficacy and safety of CHM in managing DKD have been reviewed. Traditional multi-ingredient CHM formulas such as *Liuwei Dihuang Wan* and *Shenqi Dihuang Tang*, first recorded in classical literature, have been evaluated in clinical trials and recommended for DKD in clinical practice in China (43). Recently, three registered trials of oral CHM formulas plus RAS blockade for patients with DKD have published encouraging results in terms of reducing urinary protein excretion (15, 45, 46). As a multi-compound CHM preparation, TQF has the potential to achieve a multi-target therapeutic effect for people with stage 2-4 DKD by protecting renal function, improving glucolipid metabolism, and enhancing immunity (23). It is worth conducting a rigorously designed study to explore the feasibility, efficacy, and safety of TQF compared to placebo on kidney function for people with stage 2-4 DKD.

This trial adopts a mixed methods design comprising qualitative and quantitative components. The semi-structured interviews will be conducted to explore participants’ expectations, motivations, experiences, and compliance with participating in the trial. This is a significant step toward establishing the feasibility of TQF granules and trial design. The pilot trial helps to estimate the effect size and ensure that the future full-scale trial is adequately powered to detect meaningful treatment effects since the previous study could not provide the essential data to estimate the sample size for the full-scale trial. This pilot trial will help identify any methodological issues and refine study protocols, including recruitment challenges, randomization procedures, blinding strategies, intervention delivery, outcome measures, and data collection. This will ensure that the protocol is rigorous and well-defined for a future large-scale trial. In summary, this pilot trial holds significant value in the research process before embarking on a full-scale trial. It serves as a crucial preparatory stage, offering insights that will enhance the overall quality and success of a larger-scale trial.

In this trial, the eGFR slope will be explored as the primary efficacy outcome to indicate the treatment effect of TQF in DKD. eGFR slope has been chosen as the primary efficacy outcome for a few reasons. Firstly, the eGFR slope is a validated surrogate endpoint for the progression of DKD in clinical trials (33, 34). Secondly, the eGFR slope has been widely applied as the primary outcome in clinical trials of kidney disease, such as the AASK (47), MDRD (48), and HERBAAL study (49). Slowing down this decline or maintaining stable eGFR can indicate a positive therapeutic effect, potentially delaying dialysis or transplantation. Thirdly, the eGFR slope analyses are particularly useful in measuring kidney function in early stages and for interventions with acute effects on eGFR (50). Further, eGFR slope can often be detected sooner than other clinical endpoints, for example, receipt of a kidney transplant, initiation of maintenance dialysis, and death from kidney failure, allowing for the identification of treatment effects in a relatively shorter timeframe (37).

While the current pilot trial design offers a comprehensive approach to evaluating the feasibility, efficacy, and safety of TQF in individuals with stage 2-4 DKD, there are several inherent limitations.

Firstly, the small sample size of 60 participants may limit the generalizability of the findings to a broader population. The pilot nature of the study, although appropriate for its objectives, may not fully capture the diverse characteristics and responses that could be observed in a larger cohort. Secondly, the 48-week treatment and 12-week follow-up period may not be sufficient to capture long-term effects or assess the durability of the observed changes in renal function and other outcomes. A more extended follow-up duration, for example, two years, in future trials would provide valuable insights into the sustainability of the intervention’s effects over time. Addressing such challenges in future studies could enhance the robustness of blinding procedures.

Despite these limitations, the pilot trial is a crucial first step in informing the design and implementation of a larger, more definitive trial. The insights gained from the feasibility assessment, as well as participant experiences, will guide refinements in trial procedures and potentially influence the selection of outcomes for future investigations. As we move forward, addressing these limitations will be integral to the continual improvement and optimization of the study design.

## Author contributions

ML: Investigation, Methodology, Writing – original draft, Conceptualization, Writing – review & editing. YD: Methodology, Writing – original draft, Supervision. LeiZ: Conceptualization, Methodology, Supervision, Writing – review & editing, Funding acquisition. LY: Investigation, Methodology, Writing – original draft, Funding acquisition. LaZ: Investigation, Writing – original draft. JC: Investigation, Writing – original draft. RW: Investigation, Writing – original draft. XX: Investigation, Writing – original draft. FL: Investigation, Writing – original draft. LX: Investigation, Writing – review & editing. JH: Investigation, Writing – original draft. AZ: Methodology, Writing – review & editing, Supervision. CX: Methodology, Writing – review & editing, Supervision Resources. XL: Funding acquisition, Methodology, Writing – review & editing, Conceptualization, Supervision, Resources.

## Supplementary material

Supplementary File 1 SPIRIT 2013 Checklist.

Supplementary File 2 Chemical Analysis and Quality Control.

Supplementary File 3 Ethics Approval Letters.

Supplementary File 4 Trial Protocol Appendices.

Supplementary File 5 WHO Trial Registration Dataset.
