# Supplementary material for: Oral Chinese Herbal Medicine plus usual care for diabetic kidney disease: study protocol for a randomized, double-blind, placebo-controlled pilot trial
Source: Front Endocrinol (Lausanne). 2024 Feb 8;15:1334609. doi: 10.3389/fendo.2024.1334609 (PMC10881862; doi:10.3389/fendo.2024.1334609)
Supplement: Supplementary File 2 — Chemical Analysis and Quality Control. [file DataSheet_2.docx]

Preparation and quality control of interventions

Supplementary Information and Tables

# Chemical analysis and quality control

Ultra-high performance liquid chromatography-mass spectrometry/mass spectrometry (UHPLC-MS/MS) analysis was adopted for the TQF granules. The supernatant of granules was ultrasonically extracted with 50% methanol. UHPLC analysis was performed using a Thermo UltiMate 3000 UHPLC instrument (Waltham, MA, USA). The analysis employed an ACQUITY UPLC® HSS T3 C18 column (2.1 mm × 100 mm, 1.8 μm, Waters, USA). The mobile phase comprised acetonitrile (A) and water with 0.1% formic acid (B). The flow rate was maintained at 200 μL/min. A gradient elution system was employed as follows:

| Time (min) | % A |
| --- | --- |
| 0-2 | 3 |
| 2-3 | 3-10 |
| 3-5 | 10 |
| 5-8 | 10-20 |
| 8-13 | 20 |
| 13-18 | 20-40 |
| 18-22 | 40 |
| 22-24 | 40-80 |

An injection volume of 5 μL was used for each sample.

Mass Spectrometry:

Mass spectrum acquisition was carried out using a Q-Exactive hybrid quadrupole-orbitrap mass spectrometer (Thermo Scientific, San Jose, USA) equipped with heated electrospray ionization (HESI) operating in the negative ionization mode. The main parameters optimized for mass spectrometry were set as follows:

| Parameter | Setting |
| --- | --- |
| Spray voltage | -2.5 kV |
| Capillary temperature | 350 °C |
| Auxiliary gas heater temperature | 350 °C |
| Auxiliary gas flow rate | 15 |
| Sheath gas flow rate | 40 |
| Sweep gas flow rate | 0 |

The raw data was imported into Compound Discoverer 3.0 software, and the possible molecular formulas were fitted through the extracted molecular ion chromatographic peaks. The results were then matched with the mz Vault and mz Cloud databases. Xcalibur 2.1 software was used to further analyze and identify the compounds by comparing them with relevant literature and standard substances based on the accurate m/z values of precursor and fragment ions, retention time, and the information of cleavage pattern.

Thirty-eight compounds were identified from UHPLC-MS/MS analysis of TQF (Fig 2 in the manuscript). The details of the compounds and their bioactivities are listed in the **Supplementary Table S1**.

**Table S1 Compounds** **from UHPLC-MS/MS analysis of TQF and their bioactivities**

| No. | tR/min | [M+H]+ | [M-H]^-^/[M+FA-H]^-^ | Molecular  formula | Compounds | Predominant bioactivities |
| --- | --- | --- | --- | --- | --- | --- |
| 1 | 1.21 | 175.1188 |  | C_6_H_14_N_4_O_2_ | L (+)-Arginine ^*^ | Nitric oxide synthesis, vascular health, immune support |
| 2 | 2.03 |  | 133.0126 | C_4_H_6_O_5_ | Malic acid | Energy metabolism, Krebs cycle, cellular respiration |
| 3 | 2.87 | 132.1018 |  | C_6_H_13_NO_2_ | L-Norleucine ^*^ | Protein synthesis, enzyme activity |
| 4 | 3.13 | 132.1018 |  | C_6_H_13_NO_2_ | Isoleucine | Protein synthesis, immune function, energy metabolism |
| 5 | 3.36 | 268.1045 |  | C_10_H_13_N_5_O_4_ | Adenosine | Energy transfer, cellular signaling, neurotransmission |
| 6 | 3.80 | 284.0992 |  | C_10_H_13_N_5_O_5_ | Guanosine | RNA/DNA synthesis, neurotransmission |
| 7 | 5.88 | 166.0861 |  | C_9_H_11_NO_2_ | Alanine | Glucose metabolism, energy production |
| 8 | 6.59 |  | 315.0719 | C_13_H_16_O_9_ | Gentisoyl glucoside | Antioxidant, anti-inflammatory |
| 9 | 7.79 | 355.1015 | 353.0880 | C_16_H_18_O_9_ | Neochlorogenic acid | Antioxidant, anti-inflammatory |
| 10 | 8.13 | 205.0967 |  | C_11_H_12_N_2_O_2_ | L-Tryptophan | Serotonin precursor, mood, sleep |
| 11 | 12.97 |  | 609.1442 | C_27_H_30_O_16_ | Rutin ^*^ | Antioxidant, anti-inflammatory, cardiovascular |
| 12 | 9.77 | 355.1014 | 353.0882 | C_16_H_18_O_9_ | Chlorogenic acid ^*^ | Antioxidant, glucose metabolism |
| 13 | 10.01 | 355.1016 | 353.0874 | C_16_H_18_O_9_ | Cryptochlorogenic acid | Antioxidant |
| 14 | 10.14 |  | 502.1557 | C_20_H_27_NO_11_ | Amygdalin ^*^ | Cyanide metabolism, potential anti-cancer |
| 15 | 10.45 | 177.0545 |  | C_10_H_8_O_3_ | hymecromone | Gallstone treatment |
| 16 | 10.83 | 595.1650 | 593.1507 | C_27_H_30_O_15_ | Vicenin -2 | Antioxidant, potential anti-inflammatory |
| 17 | 11.08 |  | 623.1605 | C_28_H_32_O_16_ | stellarin-2 | Limited information |
| 18 | 11.64 |  | 367.1031 | C_17_H_20_O_9_ | Feruloylquinic acid | Antioxidant, potential anti-inflammatory |
| 19 | 12.70 | 565.1541 | 563.1395 | C_26_H_28_O_14_ | vitexin- xyloside | Limited information |
| 20 | 12.79 |  | 595.1664 | C_27_H_32_O_15_ | Eriocitrin ^*^ | Antioxidant, potential cardiovascular |
| 21 | 12.97 | 447.1275 |  | C_22_H_22_O_10_ | Calycosin 7-O-glucoside ^*^ | Estrogenic, cardiovascular |
| 22 | 13.09 | 595.1643 | 593.1508 | C_27_H_30_O_15_ | Lonicerin | Limited information |
| 23 | 13.51 | 465.1017 | 463.0878 | C_21_H_20_O_12_ | Hyperoside ^*^ | Antioxidant, anti-inflammatory, cardiovascular |
| 24 | 13.78 | 465.1021 | 463.0880 | C_21_H_20_O_12_ | Isoquercitrin | Antioxidant, potential anti-inflammatory |
| 25 | 14.99 | 517.1334 | 515.1189 | C_25_H_24_O_12_ | Isochlorogenic acid B | Antioxidant |
| 26 | 15.21 | 581.1849 | 579.1703 | C_27_H_32_O_14_ | Narirutin | Antioxidant, potential anti-inflammatory |
| 27 | 15.98 | 517.1331 | 515.1191 | C_25_H_24_O_12_ | Isochlorogenic acid A ^*^ | Antioxidant |
| 28 | 16.32 |  | 447.0919 | C_21_H_20_O_11_ | Astragaline ^*^ | Immunomodulatory |
| 29 | 17.20 | 611.1953 | 609.1805 | C_28_H_34_O_15_ | Hesperidin* | Antioxidant, potential cardiovascular |
| 30 | 17.43 | 517.1335 | 515.1188 | C_25_H_24_O_12_ | Isochlorogenic acid C ^*^ | Antioxidant |
| 31 | 19.78 |  | 1003.5084 | C_48_H_78_O_19_ | Asiaticoside ^*^ | Wound healing, anti-inflammatory |
| 32 | 19.89 | 595.2011 | 593.1859 | C_28_H_34_O_14_ | Poncirin | Limited information |
| 33 | 20.20 |  | 301.0356 | C_15_H_10_O_7_ | Quercetin ^*^ | Antioxidant, anti-inflammatory, cardiovascular |
| 34 | 20.27 | 285.0757 | 283.0610 | C_16_H_12_O_5_ | Calycosin^*^ | Estrogenic, cardiovascular |
| 35 | 24.11 | 269.0811 | 267.0657 | C_16_H_12_O_4_ | Formononetin ^*^ | Anti-inflammatory, estrogenic |
| 36 | 22.49 |  | 299.0555 | C_16_H_12_O_6_ | Rhamnocitrin | Limited information |
| 37 | 23.18 |  | 263.1281 | C_15_H_20_O_4_ | naphthol[1,2-b] furan-2-one | Limited information |
| 38 | 26.08 |  | 871.4670 | C_43_H_70_O_15_ | Astragaloside II | Immunomodulatory |

^*^ Compared with reference compounds

The chemicals contained in TQF were assessed by high-performance liquid chromatography (HPLC) spectrometry. HPLC analysis was performed using a Waters 2695 instrument (Waltham, MA, USA). The parameters were as follows: Column: Xbridge® C18, 4.6 mm×250 mm, 5 μm (Waters, USA); Mobile phase: Acetonitrile (A), water with 0.1% formic acid (B). A gradient elution system was employed:

| Time (min) | % A |
| --- | --- |
| 0-5 | 5-5 |
| 5-10 | 5-10 |
| 10-30 | 10-18 |
| 30-42 | 18 |
| 42-50 | 18-25 |
| 50-55 | 25 |
| 55-60 | 25-40 |
| 60-65 | 40 |
| 65-70 | 40-80 |
| 70-80 | 80 |
| Detector Settings | |
| Wavelength | 210 nm and 340 nm |

As compared with standard reference compounds, eight constituents of TQF, including chlorogenic acid, cryptochlorogenic acid, calycosin 7-O-glucoside, hyperoside, hesperidin, isochlorogenic acid C, calycosin, nobiletin, were analyzed by HPLC (Fig 3 in the manuscript).

# Preparation of TQF granules

TQF was prepared as an oral granulated product by the Jiangyin Tianjiang Pharmaceutical Company (Jiangyin, China), a Good Manufacturing Practice (GMP) certificated company. The amount of each botanical drug in the preparation of every kilogram of TQF granules was *Astragalus mongholicus Bunge*, *Cuscuta australis R.Br.*, *Prunus davidiana (Carrière) Franch.*, *Atractylodes lancea (Thunb.) DC.*, *Citrus × aurantium L*, *Centella asiatica (L.) Urb.*, *Isaria cicadae Miquel*. The raw plant materials were acquired from commercial sources. The scientific name, species name, pharmaceutical name, Chinese Pin Yin name, proportion, and place of origin were provided in the **Supplementary Table S2**. The purity/contaminant tests showed that the TQF granules were free of heavy metals or other undesirable contaminants, which complied with the pharmacopeial criteria (2020 Edition (34)). The stability/consistency tests are being performed to indicate whether the product is stable/consistent from the start till the end of the clinical study. No equivalence test was needed since a single batch of TQF granules would be used during the clinical study.

**Table S2** The components of TQF in scientific, species, pharmaceutical, and Chinese Pin Yin names, proportion, and place of origin

| **Scientific name** | **Species name** | **Pharmaceutical name** | **Chinese pin yin** | **Proportion** | **Place of origin (Commercial supplier/province)** |
| --- | --- | --- | --- | --- | --- |
| *Astragalus mongholicus Bunge* | *Fabaceae* | *Astragali mongholici radix* | *Huang qi* | 20 | Anhui Cunzhen Pharmaceutical Co., Ltd./Gansu |
| *Cuscuta australis R.Br.* | *Convolvulaceae* | *Cuscutae semen* | *Tu si zi* | 15 | Anhui Baiyutang Traditional Chinese Medicine Decoction Pipe Co., Ltd./Inner Mongolia |
| *Prunus davidiana (Carrière) Franch.* | *Rosaceae* | *Persicae semen* | *Tao ren* | 10 | Anhui Haixin Pharmaceutical Co., Ltd./Gansu |
| *Atractylodes lancea (Thunb.) DC.* | *Asteraceae* | *Atractylodis lanceae rhizoma* | *Cang zhu* | 10 | Anhui Fengliaoxing Pharmaceutical Co., Ltd./Inner Mongolia |
| *Citrus × aurantium L.* | *Rutaceae* | *Aurantii amari epicarpium et mesocarpium* | *Chen pi* | 10 | Anhui Haixin Pharmaceutical Co., Ltd./Zhejiang |
| *Centella asiatica (L.) Urb.* | *Apiaceae* | *Centellae asiaticae herba* | *Ji xue cao* | 15 | Anhui Haixin Pharmaceutical Co., Ltd./Anhui |
| *Isaria cicadae Miquel* | *Cordycipitaceae* | *Isaria cicadae Miquel* | *Chan hua* | 8 | Anhui Jinguoyuan Pharmaceutical Co., Ltd./Anhui |

The extraction procedure of the TQF product was: firstly, do two rounds of decocting for the mixture of seven raw botanical drugs with water. Secondly, the decoctions from the previous step are merged, then filtered and concentrated into a transparent paste. Next, maltodextrin was added to the paste till it was dissolved and then spray-dried. The mixture was made into 1000 grams of granules. In the end, the granules were packaged into packets of 7.5 grams. A single batch was used for this clinical study.

# Preparation of placebo

The TQF placebo was produced by the same manufacturer using a concentrate of caramel pigments, gardenia yellow pigment, sunset yellow pigment, and dextrin with the proportion of 1.5: 1: 1: 0.8. Other components were sucrose octaacetate (3%), suspending agent (dextrin and titanium dioxide, 0.8%), and lactose (10%). The placebo was made in granules and packaged into packets of 7.5 grams. According to the 2020 Chinese pharmacopeia, the components in the placebo are food-grade at this dosage and have no known side effects with oral consumption. The blinding test of the TQF granules and placebo showed the package and weight were identical, and the appearance, color, smell, and taste were similar. Critical information on chemicals, manufacturing, and control of TQF and TQF placebo is being conducted by the pharmaceutical team of GPHCM. The chemical composition of TQF is validated using high-performance liquid chromatography/mass spectrometry.
