# Supplementary material for: Oral Chinese Herbal Medicine plus usual care for diabetic kidney disease: study protocol for a randomized, double-blind, placebo-controlled pilot trial
Source: Front Endocrinol (Lausanne). 2024 Feb 8;15:1334609. doi: 10.3389/fendo.2024.1334609 (PMC10881862; doi:10.3389/fendo.2024.1334609)
Supplement: Supplementary File 3 — Ethics Approval Letters. [file DataSheet_3.pdf]

广东省中医院伦理委员会

Ethics Committee of Guangdong Provincial Hospital of Chinese Medicine

伦理审查批件

Approval Notice

批件号：广东省中医院伦理委员会YF2022-152-01

审查日期	初审：2022年07月15日；		
审查地点	广东省中医院研修楼20楼2005-2006（长形会议室）		
临床研究批文	无		
临床研究项目	糖肾祛湿方治疗2-4期糖尿病肾病患者的随机对照预试验与定性研究		
审查文件	1. 初始审查申请表包括研究经济利益声明<br>2. 研究方案(版本号/日期：V1.0/20220705)<br>3. 向受试者提供的知情同意书（包括研究简介和签字页）(版本号/日期：V1.0/20220705)<br>4. 招募受试者的材料（包括广告）(版本号/日期：V1.0/20220705)<br>5. 临床病例报告表(版本号/日期：V1.0/20220705)<br>6. 主要研究者简历<br>7. 主要研究者GCP培训证书<br>8. 专业组人员名单		
申办者/任务下达单位	无		
临床研究单位	广东省中医院(大院肾内科)		
主要研究者	刘旭生,刘枚芳		
伦理审查方式	会议审查		
审查委员	韩云,刘奕明,许丽梅,肖静,萧蕙,李晓彦,刘博,朱飞,冯昊禧		
审查意见	根据国家食品药品监督管理局《药物临床试验质量管理规范》、《医疗器械临床试验质量管理规范》、《药物临床试验伦理审查工作指导原则》，卫计委《涉及人的生物医学研究伦理审查办法》、《干细胞临床研究管理办法（试行）》，国家中医药管理局《中医药临床研究伦理审查平台建设规范》，以及世界医学会《赫尔辛基宣言》和国际医学科学组织委员会《涉及人的健康相关研究国际伦理准则》的伦理原则，经本伦理委员会审查，同意按照上述临床研究方案和上述已通过审查的文件进行临床研究。		
伦理委员会声明	本批件将在各中心机构及其伦理委员会备案。如果对方案在本机构的可行性（包括研究者的资格与经验、设备与条件等）有不同意见，请及时与本伦理委员会联系。<br>如项目暂停/提前终止/完成临床研究，或发生严重不良事件以及影响研究风险受益比的非预期不良事件，请及时报告伦理委员会。如临床研究方案、知情同意书的任何修改，主要研究者更换，应及时通知伦理委员会，经审查批准后执行。发现影响受试者参加研究意愿的违反方案情况应及时报告。**请在预计跟踪审查日期前1个月提交研究进度/结题报告。**<br>凡涉及中国人类遗传资源、需要报批的研究项目，须在获得中国人类遗传资源管理办公室批准后才能开始研究。非以产品注册为目的的、干预性临床研究，须在医学研究登记备案信息系统（http:114.255.48.20）注册后方可开展研究。		
批件有效期	自2022年07月15日起<br>至2024年07月15日止	跟踪审查频率<br>预计审查日期	12个月<br>2023年07月15日

联系电话	020-81887233转35943，联系人：李晓彦
主任/副主任委员签字	
广东省中医院伦理委员会（盖章）	
日期：2022年07月15日	

Ethics Committee of Guangdong Provincial Hospital of Chinese Medicine Approval Notice

Approval No. YF2022-152-01

**Date of Review**	15 July 2022
**Place of Review**	Room 2005-2006, Advanced Training Building, Guangdong Provincial Hospital of Chinese Medicine, No.111 Dade Road, Guangzhou, Guangdong Province, China
**Clinical Research Approval**	N/A
**Clinical Research Project**	Tangshen Qushi Formula for people with stage 2-4 diabetic kidney disease: a pilot randomised controlled trial and qualitative study
**Reviewed Documents**	1. Application Form of Initial Review including Statement for Conflict of Interest<br>2. Study Protocol (ver./date:V1.0/2022005)<br>3. Inform Consent Form (ver./date:V1.0/2022005)<br>4. Recruiting Advertisement (ver./date:V1.0/2022005)<br>5. Case Report Form (ver./date:V1.0/2022005)<br>6. Curriculum Vitae of Principal Investigator<br>7. Principal Investigator's Certificate of GCP Training<br>8. List of investigation group members
**Sponsor**	N/A
**Research Department**	Department of Nephrology, Guangdong Provincial Hospital of Chinese Medicine
**Principal Investigator**	Xusheng Liu, Meifang Liu
**Review Type**	Full Board Review
**Review Members**	Yun Han, Yiming Liu, Limei Xu, Jing Xiao, Hui Xiao, Xiaoyan Li, Bo Liu, Fei Zhu, Haoxi Feng
**Comments**	In compliance to the Good Clinical Practice for Clinical Trial of New Drugs, Quality Management Standards for Medical Device Clinical Trials, Guidelines for Ethics Review of Drugs Clinical trial (approved by State Food and Drug Administration), Ethics Review for Biomedical Research Involving Human Subjects, Administrative Regulation for Clinical Research of Stem Cells (Trial) (issued by the National Health and Family Planning Commission), Standards for Ethics Review Platform Construction of Chinese Medical Research (issued by National Administration of Traditional Chinese Medicine), Declaration of Helsinki, International Ethical Guideline for Biomedical Research involving Human Subjects, the Ethics Committee of Guangdong Provincial Hospital of Chinese Medicine agrees this clinical study design and perform the study with the reviewed documents.
**Institutional Ethics Committee Statement**	This approval document will be conducted archival filing in other institutions of their ethics committee. Please contact us if there is any different opinion about the feasibility of this research (qualification/ experience of investigators, research facilities, etc.).<br>Please notify us when this research is suspended/terminated/completed. Severe adverse event or any unexpected adverse event, which might influence the balance of risk and benefit of the study, should be reported to the ethics committee in time. Re-review is needed in case of protocol/inform consent form modification and principal investigator replacement, and they should be implemented after further approval. Any situation which violates the program and will affect the subject's willingness to participate should be promptly reported. Please submit the study progress/final report a month before approval document expire date.<br>If the research involving human genetic resources, it must be approved by Human Genetic Resources Management Office of China before it starts.

	Clinical intervention studies which are not for the purpose of product registration shall be registered in the Medical Research Registration Information System (http:114.255.48.20) before they are carried out.		
**Term of Validity**	15 July 2022 to<br>15 July 2024	**Periodicity of Follow-up**<br>**Date of Next Review**	12 months<br>15 July 2023
**Contact Information**	Tel: 8620-81887233 ext35943, Contact Person: Xiaoyan Li,		
**Signature of Chariman/ Vice Chairman**	Yun Han		
	Ethics Committee of Guangdong Provincial Hospital of Chinese Medicine (Stamp)		
	Date: 15 July 2022		

文件编号:AF/SC-08/04. 0

伦理审查意见

意见号：伦审 2022-021-02

项目名称	糖肾祛湿方治疗糖尿病肾病的随机对照临床研究		
项目来源	广东省中医院		
负责单位	广西中医药大学第一附属医院		
本中心主要研究者	蓝芳		
审查类别	复审	审查方式	快速审查
审查日期	2022 年 05 月 09 日	审查地点	NA
审查委员	初审会审：桂雄斌，韦艾凌，黄彬，张曼，罗继红， 俞渊，黎军宏，张荣臻，陈炜， 罗<br>陈德华，黄明政，杨增艳，武丽 复审快审 继红，张荣臻		
审查结果	同意		
同意文件	1. 科研部的《递交伦理审查申请》<br>2. 临床研究方案（版本号:002,版本日期:20211026）<br>3. 知情同意书（版本号：002,版本日期：20211026）<br>4. 招募材料（版本号：002,版本日期:20211026）<br>5. 其他-试验文档修正对照表<br>6. 病例报告表（版本号：001,版本日期：2021 年 10 月 07 0）<br>7. 研究者手册（版本号：001,版本日期：2022 年 02 月 23 0 ）<br>8. GCP 培训证书<br>9. 主要研究者专业履历<br>10. 研究人员及其研究分工<br>11. 研究者：研究经济利益声明<br>12. 组长单位伦理委员会批件		

审查意见

根据《药物临床试验质量管理规范（2020）》、《涉及人的生物医学研究伦理审查办法（2016）》、《医疗器械临床试验规定（2004）》、WMA《赫尔辛基宣言》和 CIOMS《人体生物医学研究国际道德指南》的伦理原 则，本伦理委员会按照上述文件履行伦理委员会职责，其组成及操作规程符合相关法律法规。经本伦理委 员会审查，同意按所批准的临床研究方案、知情同意书、招募材料等开展本项研究。

研究过程中若变更主要研究者，对临床研究方案、知情同意书、招募材料等的任何修改，请申请人提 交修正案审查申请。

发生可疑且非预期严重不良反应，请研究者及时提交可疑且非预期严重不良反应报告。

按照伦理委员会规定的年度/定期跟踪审查频率，申请人在截止日期前一个月提交研究进展报告。

当出现任何可能显著影响试验进行、或增加受试者危险的情况时，请申请人及时向伦理委员会提交书 面报告。

研究纳入了不符合标准或符合排除标准的受试者，符合中止试验规定而未让受试者退出研究，给予错 误治疗或剂量，给予方案禁止的合并用药等没有遵循从方案开展研究的情况；或可能对受试者的权益/健 康及研究的科学性造成不良影响等违背 GCP 原则的情况，请申办者/监查员/研究者提交违背方案报告。

申请人暂停或提前终止临床研究，请及时提交暂停/终止研究报告。

完成临床研究，请申请人提交研究完成报告。

跟踪审查频率	12 个月	下次跟踪审查截止日期	2023 年 05 月 09 日
有效期	2022-05-10 ~ 2023-05-09		
联系人/联系电话	黎老师/0771-5623553		
伦理委员会	广西中医药大学第一附属医院医学伦理委员会（盖章）		
主任委员签字		日期	2022 年 05 月 10 日

Document No.AF/SC-08/04. 0

Ethics Committee of Guangxi Hospital of Chinese Medicine Approval Notice

Approval No. LS2022-021-02

**Clinical Research Project**	Tangshen Qushi Formula for people with stage 2-4 diabetic kidney disease: a pilot randomised controlled trial and qualitative study		
**Sponsor**	Guangdong Provincial Hospital of Chinese Medicine		
**Research Site**	Guangxi Hospital of Chinese Medicine		
**Principal Investigator**	Fan Lan		
**Review Type**	Full Board Review/Expedited Review		
**Date of Review**	9 May 2022	**Place of Review**	NA
**Review Members**	Xiongbin Gui, Ailing Wei, Bin Huang, Man Zhang, Jihong Luo, Junhong Li, Rongzhen Zhang, Wei Chen, Yuan Yu, Dehua Chen, Mingzheng Huang, Zengyan Yang, Li Wu		
**Review Result**	Approved		
**Reviewed Documents**	1. Application Form of Initial Review from Research Management Office<br>2. Study Protocol (ver./date:002/20211026)<br>3. Inform Consent Form (ver./date:002/20211026)<br>4. Recruiting Advertisement (ver./date:002/20211026)<br>5. Other Document_Revised Comparison Table<br>6. Case Report Form (ver./date:001/20211007)<br>7. Investigator's Brochure (ver./date:001/20220223)<br>8. Principal Investigator’s Certificate of GCP Training<br>9. Resume of Principal Investigator<br>10. List of Investigation Group Members<br>11. Statement for Conflict of Interest<br>12. Approval Notice from Ethics Committee of Responsible Research Site		

Comments and statements:

In compliance to the Good Clinical Practice (GCP) for Clinical Trial of New Drugs (2020 edition), Ethics Review for Biomedical Research Involving Human Subjects (2016 edition), Quality Management Standards for Medical Device Clinical Trials (2004 edition), Declaration of Helsinki and International Ethical Guideline for Biomedical Research involving Human Subjects, the Ethics Committee of Guangxi Hospital of Chinese Medicine agrees this clinical study design and perform the study with the reviewed documents.

Re-review is needed in case of protocol/inform consent form/recruitment advertisement modification and principal investigator replacement, and they should be implemented after further approval.

Any unexpected serious adverse event should be reported to the ethics committee in time.

Please submit the study progress/final report a month before approval document expire date according the requirements of periodicity of follow-up.

Any situation which might significantly affect the trial procedure or might increase the risk of participants should be promptly reported in written documents.

Sponsor/monitor/investigator should submit protocol violation report to the ethics committee if any situation that violates the protocol or GCP principles happens: enrol ineligible participants, or fail to withdraw participants who meet the termination criteria, or give the wrong drug/dosage to participants, or give the forbidden concomitant medication to participants, or any situation that might affect the benefit/health of participant or impair the significance of this research study.

Please submit the study completion report when this trial is completed.

**Periodicity of Follow-up**	12 months	**Date of Next Review**	9 May 2023
**Term of Validity**	From 10 May 2022 to 9 May 2023		
**Contact Information**	Tel: 0771-5623553, Contact Person: Dr. Li		
**Ethics Committee**	Ethics Committee of Guangxi Hospital of Chinese Medicine (Stamp)		
**Signature of Chariman**	Xiongbin Gui	**Date**	10 May 2022
