# Supplementary material for: Oral Chinese Herbal Medicine plus usual care for diabetic kidney disease: study protocol for a randomized, double-blind, placebo-controlled pilot trial
Source: Front Endocrinol (Lausanne). 2024 Feb 8;15:1334609. doi: 10.3389/fendo.2024.1334609 (PMC10881862; doi:10.3389/fendo.2024.1334609)
Supplement: Supplementary File 4 — Trial Protocol Appendices. [file DataSheet_4.pdf]

Appendix 1: Advertisement Leaflet

Chinese herbal medicine Tangshen Qushi Formula for people with stage 2-4 DKD

If you are diagnosed with stage 2-4 diabetic kidney disease and considering Chinese herbal medicine to slow the progression of DKD, then we invite you to participate in a study using a promising Chinese herbal medicine Tangshen Qushi Formula (TQF) for preventing the progression of DKD.

Nephrology Department of Guangdong Provincial Hospital of Chinese Medicine is running a clinical study on TQF to benefit people with stage 2-4 DKD. We want to explore how well this formula works for protecting renal function and delaying the progression of DKD. We need your help.

You will receive this formula or placebo for treatment for 48 weeks. This way we can work out whether the real treatment helps you slow the progression of disease. If you are recruited in the study, we will offer you the herbal treatment throughout the study, at no cost.

For further details or get involved, please contact the Investigator:

Dr Meifang Liu on (+86) 13751728376 or via email on meifangliu@gzcum.edu.au & s3886552@student.rmit.edu.au

This study has been approved by the Human Research Ethics Committee of GPHCM/GXHCM and registered at RMIT University.

Appendix 2: Plain Language Statement

To Study Participants

Plain Language Statement

Date:
Full Study Title: TANGSHEN QUSHI FORMULA FOR PEOPLE WITH STAGE 2-4 DIABETIC KIDNEY DISEASE: A PILOT RANDOMISED CONTROLLED TRIAL AND QUALITATIVE STUDY

If you are aged 18 years or older and diagnosed with stage 2-4 diabetic kidney disease with the estimated glomerular filtration rate within the latest 12 weeks is between 15 (included) and 90 $mL/min/1.73m^2$ and the urinary protein to creatinine ratio is less than 3500 mg/g, you are invited to participate in this research study.

DKD has become the leading cause of kidney failure, which accounts for about 30% ~ 50% of ESKD worldwide, progressing very fast and requiring renal replacement therapy, causing the heavy socioeconomic and public health burden. However, the progress of DKD still cannot be delayed. The current western treatment options for DKD are limited, especially in terms of delaying the progression of DKD, calling an urgent need for alternative therapies. In a single-arm clinical trial, a novel Chinese herbal medicine (CHM) for DKD, Tangshen Qushi Formula (TQF), showed a potential effect on protecting renal protection function and slowing the progression of DKD.

If you take part in this study you will have a 50/50 chance of being randomly assigned to either the TQF group or the placebo group. This design will allow us to explore the therapeutic efficacy and safety of the TQF and the placebo and make sure changes are not just happening by chance.

The study will run over a period of 62 weeks and you are invited to visit the clinical study sites at Guangdong Provincial Hospital of Chinese Medicine (GPHCM). In total, you will visit the study site 17 times, once for initial assessment, once for randomisation and consent informed, 12 visits for treatment and 3 visits at the period the follow up. Each visit may take 30-45 minutes. During the 48-week treatment phase, you will either receive the TQF group or the placebo treatment. If you are in the placebo group, you will be offered 12 weeks of TQF after the end of the study period at no cost. If possible, you will be invited to join an individual interview at the end of trial.

Your support is much appreciated, and participation is voluntary; you may withdraw at any time, and request access to your information at any time. The information you provide will be treated as confidential. Data collected from this study will be stored in locked cabinets and all electronic files will be stored in a password-protected GPHCM network. Access will only be by authorised personnel involved in this research study. Data collected will be analysed by the investigators. Reports on the findings from this study will give group data only and your individual identity will remain anonymous.

If you have any queries regarding this study please feel free to contact Dr Meifang Liu on 13751728376 or email meifangliu@gzucm.edu.cn & s3886552@student.rmit.edu.au.

Yours Sincerely,

Meifang Liu

Appendix 3: Screening Questionnaire

Screening Questionnaire

PLEASE WRITE CLEARLY TO HELP US MAINTAIN ACCURATE RECORDS

Study site: Date:

Name: ________ Gender □ Female □ Male Age: Medical Card ID ________

Address ________ Phone number: ________

Medical Card ID		Phone number			
Primary Diagnosis					
Inclusion criteria	(1) Aged 18 years and over;			□Yes	□No
	(2) Confirm to the diagnostic criteria of diabetic kidney disease;			□Yes	□No
	(3) eGFR less than 90 (excluded) mL/min/1.73m^2 and no less than 15 (included) and (calculated by CKD-EPI equation) and the change of eGFR within the latest 12 weeks before inclusion less than 30%;			□Yes	□No
	(4) Urinary protein creatinine ratio (PCR) less than 3500 mg/g;			□Yes	□No
	(5) Agree to make themselves available for the period of the study; and				
	(6) Provide written informed consent.			□Yes	□No
Exclusion criteria	(1) History of maintenance dialysis treatment or kidney transplantation for at least 90 days;			□Yes	□No
	(2) Plan to accept maintenance dialysis treatment or kidney transplantation within 2 weeks;			□Yes	□No
	(3) Diagnosed with Acute urinary tract infection within 2 weeks before randomisation;			□Yes	□No
	(4) Diagnosed with other primary chronic kidney diseases (such as glomerulonephritis, chronic pyelonephritis, ischemic kidney disease, etc.) and the average eGFR decreased by more than 30% within 12 weeks before enrolment;			□Yes	□No
	(5) History of abnormal liver function (AST or ALT or alkaline phosphatase > 3 TIMES ULN), or other known acute and chronic active hepatitis, cirrhosis;			□Yes	□No
	(6) History of Acute coronary syndrome, heart failure (NYHA grade III-IV), or acute myocardial infarction, unstable angina, stroke, or any other cardiovascular or cerebrovascular accident requiring revascularisation surgery within 12 weeks prior to enrolment, or a revascularisation operation is urgently needed after recruitment;			□Yes	□No
	(7) History of severe acute diabetic complications include but not limit to ketoacidosis, lactic acidosis, hyperotonic non-ketotic diabetic coma, hypoglycemic coma occurred within 24 weeks before screening;			□Yes	□No
	(8) History of active malignant tumor disease within 5 years prior to screening;			□Yes	□No
	(9) History of mental illness or language barrier before screening and are unable or			□Yes	□No

	unwilling to cooperate;		
	(10) History of autoimmune diseases (for example, systemic lupus erythematosus) and are receiving hormone or immunosuppressive therapy before screening;	□Yes	□No
	(11) Be allergic to any medications or ingredients Astragalus membranaceus, Salt dodder, Blanched peach kernel, Atractylodes, Tangerine Peel, Centella asiatica, Cicada Flower; Or intolerance to these medications or ingredients (e.g. lactose intolerance);	□Yes	□No
	(12) Participate in other clinical trials or have participated in trials of other drugs within 12 weeks prior to screening (refer to those who are enrolled and receiving trial drugs);	□Yes	□No
	(13) Female during pregnancy or lactation;	□Yes	□No
	(14) Other conditions that the investigator judged inappropriate for the study.	□Yes	□No

Informed consent	□Yes □No	Recruit	□Yes □No
The reasons for not being included are as follows:			
Any note:			

Investigator's signature: Date: ___ ___ ___ ___ / ___ ___ / ___ ___

Appendix 4: General Information Questionnaire

General Information Questionnaire

STUDY TITLE: TANGSHEN QUSHI FORMULA FOR PEOPLE WITH STAGE 2-4 DIABETIC KIDNEY DISEASE: A PILOT RANDOMISED CONTROLLED TRIAL AND QUALITATIVE STUDY

Please write properly.

Date: ________________

General Information

1. Name: ______________________Gender: ___________Date of Birth: __________Age: __________
2. Address: ___________________________________City: _____________ Zip Code: ____________
3. Home phone: ________________________ Work phone: _______________ Cell: _____________
   Best time to call you? _____________________________
4. Marital status: ☐ Single ☐ Married ☐ Divorced ☐ Widowed
5. How many people live in your household? __________
6. Is there anyone who will help you with your diabetes care? ☐ Yes ☐ No
   If "yes," who? ____
7. Occupation: _______________________________________ Work hours: ______________________
8. Last grade of school completed: ____
9. Any religion preference? ____

Appendix 5: Composite Renal Endpoint Event Form

Composite Renal Endpoint Event Form

Is there an endpoint event? □Yes □No
If yes, please fill in the following table:

	Endpoint event	Date of occurrence	Measurements
**□ Endpoint event 1: Receipt of a kidney transplant**			
**□ Endpoint event 2: Initiation of maintenance dialysis (if "yes", please complete the following details, at least select one item)**			
	(Decrease of GFR: eGFR $\leq$ 10 mL/min/1.73m^2(EPI equation). No change after one week of conservative medical treatment or occur twice out of three times within one month.	Date	□ Hemodialysis<br>□ Peritoneal dialysis<br>□ Mixed Hemodialysis and Peritoneal dialysis
	(Hyperkalemia: serum potassium $\geq$ 6.5 mmol/l. No change after one week of conservative medical treatment or occur twice out of three times within one month.		
	Metabolic acidosis: the binding capacity of carbon dioxide was less than 13mmol / L, and no change after one week of conservative treatment in internal medicine or twice out of three times within one month		
	Uremic pericarditis or pleurisy		
	Uremic encephalopathy		
	Refractory hypertension		
	Congestive heart failure		
	Obvious bleeding tendency		
	Progressive malnutrition (including anorexia, weight loss, and decreasing serum albumin)		
	**□ End point event 3: a sustained low GFR and a sustained percent decline in GFR**		
	□ Sustained low GFR means GFR <15 mL/min per 1.73 m^2 sustained over at least 4 weeks.	Date	___ ___ ___ ___ / ___ ___ / ___ ___ **(year/month/day)**
	□ Sustained percent decline in GFR means percent decline in GFR of 40% from a baseline start point sustained over at least 4 weeks.		
**□ Endpoint 4: Death from kidney failure**<br>Date of death: ___ ___ ___ ___ / ___ ___ / ___ ___ **(year/month/day)**			

Signature of researcher: ___________ Date: ___ ___ ___ ___ / ___ ___ / ___ ___

Appendix 6: 30-Day Medication Diary

30-Day Medication Diary

Site Name: ___________ Your Name: ____________ Starting Date: ___ ___ ___ ___ / ___ ___ / ___ ___ (yyyy/mm/dd)

Items	Day 1	Day 2	Day 3	Day 4	Day 5	Day 6	Day 7	Day 8	Day 9	Day 10
Have you taken the medication? #1										
Is there any symptom after taking the medications? If yes, please write down the symptoms. #2										
How do you manage the symptoms? #3										
Items	Day 11	Day 12	Day 13	Day 14	Day 15	Day 16	Day 17	Day 18	Day 19	Day 20
Have you taken the medication? #1										
Is there any symptom after taking the medications? If yes, please write down the symptoms. #2										
How do you manage the symptoms? #3										
Items	Day 21	Day 22	Day 23	Day 24	Day 25	Day 26	Day 27	Day 28	Day 29	Day 30
Have you taken the medication? #1										
Is there any symptom after taking the medications? If yes, please write down the symptoms. #2										
How do you manage the symptoms? #3										

Notes:

#1 The medications means the prescribed granules of this project by investigators at each visit.

#2 Symptoms should be recorded in as much detail as possible like start time, stop time, severity (Mild, Moderate, Severe, Life-Threatening), relationship with the medications (Not related, Unlikely related, Possibly related, Probably related, Definitely related).

#3 Action taken to the symptoms (None, Dose modification, Medical Intervention, Hospitalisation, Intervention discontinued, Other).

Appendix 7: Adverse Event Form

Adverse Event Form

STUDY TITLE: TANGSHEN QUSHI FORMULA FOR PEOPLE WITH STAGE 2-4 DIABETIC KIDNEY DISEASE: A PILOT RANDOMISED CONTROLLED TRIAL AND QUALITATIVE STUDY

**Site Name:________________________**<br>**Participant ID:_________________________**	**This form is cumulative and captures adverse events of a single participant throughout the study.**

Severity	Study Intervention	Action Taken Regarding Study Intervention	Outcome of AE	Expected	Serious Adverse Event (SAE)
1 = Mild<br>2 = Moderate<br>3 = Severe<br>4 = Life-Threatening	0 = Not related<br>1 = Unlikely related<br>2 = Possibly related<br>3 = Probably related<br>4 = Definitely related	0 = None<br>1 = Dose modification<br>2 = Medical Intervention<br>3 = Hospitalisation<br>4 = Intervention discontinued<br>5 = Other	1 = Resolved<br>2 = Recovered with minor sequelae<br>3 = Recovered with major sequelae<br>4 = Ongoing/Continuing treatment<br>5 = Condition worsening<br>6 = Death<br>7 = Unknown	1 = Yes<br>2 = No	1 = Yes<br>2 = No (if yes, complete SAE form)

At end of study only: Check this box if participant had no adverse events ☐ **No**

Adverse Event	Start Date	Stop Date	Severity	Relationship	Action Taken	Outcome of AE	Expected?	SAE?

Signature of investigator: ____________________

Date: ___ ___ ___ ___ / ___ ___ / ___ ___ (year/month/day)

Appendix 8: Serious Adverse Event Form

Serious Adverse Event (SAE) Report Form

STUDY TITLE: TANGSHEN QUSHI FORMULA FOR PEOPLE WITH STAGE 2-4 DIABETIC KIDNEY DISEASE: A PILOT RANDOMISED CONTROLLED TRIAL AND QUALITATIVE STUDY

**Site Name: ______________________**<br>**Participant ID: ____________________**	**Date Participant Reported/Date of Site Awareness:**<br>__ ___ ___ ___ / ___ ___ / ___ ___ **(year/month/day)**

SAE Event Term (Diagnosis, ex: Stroke, Myocardial Infarction).
SAE onset date: ___ ___ ___ ___ / ___ ___ / ___ ___ (year/month/day)
SAE stop date: ___ ___ ___ ___ / ___ ___ / ___ ___ (year/month/day)
Location of SAE:____
Was this an unexpected adverse event? ☐ Yes ☐ No
Diagnosis for study participation:__________
Brief description of the nature of the SAE (attach description if more space is needed): _________
Category of the SAE:

☐ Date of death ___/_____/_______
☐ Life threatening
☐ Hospitalisation – initial or prolonged
☐ Disability/incapacity
☐ Congenital anomaly/birth defect
☐ Required intervention to prevent permanent impairment
☐ Other:_

Intervention type:

☐ Medication or nutritional supplement (specify):____
☐ Device (specify):____
☐ Surgery (specify):____
☐ Behavioural/lifestyle (specify):_____

Relationship of event to intervention:

☐ Unrelated (clearly not related to the intervention)
☐ Possible (may be related to the intervention)
☐ Definite (clearly related to the intervention)

Was study intervention discontinued due to event? ☐ Yes ☐ No
What medications or other steps were taken to treat the SAE?_____
List any relevant tests, laboratory data, and history which include pre-existing medical conditions: ______
Was this event of related endpoint?
Type of report:

☐ Initial
☐ Follow-up
☐ Final

Signature of principal investigator:_______________
Date: ___ ___ ___ ___ / ___ ___ / ___ ___ (year/month/day)

Appendix 9: Record Sheet of the Compliance Check

Participant's ID	Visit Date & Week	Sequential Number of the Container	Weight of Returned Medications	Investigator's Signature

Appendix 10: Credibility of Blinding Questionnaire

STUDY TITLE: TANGSHEN QUSHI FORMULA FOR PEOPLE WITH STAGE 2-4 DIABETIC KIDNEY DISEASE: A PILOT RANDOMISED CONTROLLED TRIAL AND QUALITATIVE STUDY

Credibility of Blinding Questionnaire

For assessing the effectiveness of the blinding procedure employed in this study, please indicate which treatment you believe you have received (please √):

☐ Tangshen Qushi Formula granule treatment

☐ Placebo treatment

☐ Not sure

Appendix 11: Dampness Syndrome Scale of Chinese Medicine

Dampness Syndrome Scale of Chinese Medicine

Instructions: Please tick "√" at the corresponding numbers for each item based on your experience and feelings over the past 2 weeks. If you're not sure how to answer a question, choose the answer that most closely matches your reality or feeling.

1. Obesity	①Not at all	②Light	③Mild	④ Severe	⑤ Extremely Severe
2. Heavy body	①Not at all	②Light	③Mild	④ Severe	⑤ Extremely Severe
3. Hiding fever [1]	①Not at all	②Light	③Mild	④ Severe	⑤ Extremely Severe
4. Reluctant sweating	①Not at all	②Light	③Mild	④ Severe	⑤ Extremely Severe
5. Idleness and laziness	①Not at all	②Light	③Mild	④ Severe	⑤ Extremely Severe
6. Somnolence	①Not at all	②Light	③Mild	④ Severe	⑤ Extremely Severe
7. Head heaviness as if swathed	①Not at all	②Light	③Mild	④ Severe	⑤ Extremely Severe
8. Greasy face or hair	①Not at all	②Light	③Mild	④ Severe	⑤ Extremely Severe
9. Dirty face	①Not at all	②Light	③Mild	④ Severe	⑤ Extremely Severe
10. Sticky eyes	①Not at all	②Light	③Mild	④ Severe	⑤ Extremely Severe
11. Sticky slimy sensation in the mouth	①Not at all	②Light	③Mild	④ Severe	⑤ Extremely Severe
12. Halitosis	①Not at all	②Light	③Mild	④ Severe	⑤ Extremely Severe
13.Thirsty but not wanting to drink water	①Not at all	②Light	③Mild	④ Severe	⑤ Extremely Severe
14. Excessive phlegm	①Not at all	②Light	③Mild	④ Severe	⑤ Extremely Severe
15. Distention and fullness in the stomach	①Not at all	②Light	③Mild	④ Severe	⑤ Extremely Severe
16.Poor appetite	①Not at all	②Light	③Mild	④ Severe	⑤ Extremely Severe
17.Nausea or vomiting	①Not at all	②Light	③Mild	④ Severe	⑤ Extremely Severe

18. Soreness and weakness of waist	①Not at all	②Light	③Mild	④ Severe	⑤ Extremely Severe	
19. Soreness of joints and muscle	①Not at all	②Light	③Mild	④ Severe	⑤ Extremely Severe	
20. Heavy limbs	①Not at all	②Light	③Mild	④ Severe	⑤ Extremely Severe	
21. Sloppy stool	①Not at all	②Light	③Mild	④ Severe	⑤ Extremely Severe	
22.Frequent bowel movements	①Not at all	②Light	③Mild	④ Severe	⑤ Extremely Severe	
23. Sticky and non-smooth bowel movements	①Not at all	②Light	③Mild	④ Severe	⑤ Extremely Severe	
24. Excessive vaginal discharge/ moist scrotum	①Not at all	②Light	③Mild	④ Severe	⑤ Extremely Severe	
25. Ulcer and sore in skin	①Not at all	②Light	③Mild	④ Severe	⑤ Extremely Severe	
26. Thick tongue coating *	①Not at all	②Light	③Mild	④ Severe	⑤ Extremely Severe	舌苔厚
27. Slimy tongue coating *	①Not at all	②Light	③Mild	④ Severe	⑤ Extremely Severe	舌苔腻
28. Slippery tongue coating *	①Not at all	②Light	③Mild	④ Severe	⑤ Extremely Severe	舌水滑多津
29. White line on the tongue*2	①Not at all	②Light	③Mild	④ Severe	⑤ Extremely Severe	舌有缨线
30. Enlarged tongue*	①Not at all	②Light	③Mild	④ Severe	⑤ Extremely Severe	舌体胖大

Signature of participant: _______________

Date: ___ ___ ___ ___ / ___ ___ / ___ ___(year/month/day)

* Scan the QR code on the right to view the tongue map for reference.

[1] Hiding fever: Feeling like having fever, but it is not hot when touching the skin.
[2] The white line on the tongue: The sides of tongue (0.5-1cm from the centre) are deposited by saliva foam.

Appendix 12: Interview Guide

Version: Dated: ___ ___ ___ ___ / ___ ___ / ___ ___ (year/month/day)
Participant number: ________

Qualitative study: Participants' experiences and acceptability of TQF for stage 2-4 DKD

INTERVIEW GUIDE FOR PARTICIPANTS
** The questions below are examples of questions that may be used to guide interviews with participants. Questions do not have to be asked in this order, and not all questions have to be covered**
Introduction
Hi, my name is Meifang Liu. Thank you for taking part in this interview. Researchers and health professionals at Guangdong Provincial Hospital of Chinese medicine want to find out whether TQF assists individuals with stage 2-4 DKD to slow the progression of DKD.
We would like to ask you questions about the treatment you received in the pilot trial. If at any time you would like to stop the interview, please let us know and we will stop. You can change your mind about talking to me at any time before or during the interview and stop the interview at any time. You can choose not to answer a question.
Are you happy to continue? [If no, thank them for their time and end the interview; if yes, continue].

Thank you [name] for agreeing to take part. We will use your feedback and the feedback of others to write a summary of what people have told us. There will be absolutely no identification of any real names or identification of where you live or which hospitals or health professionals you have seen.

Are you happy for me to record the interview? Do you have any questions before we start?

CONTEXT: TO UNDERSTAND WHAT WORKED, WHAT DIDN'T WORK, AND WHY/ WHY NOT.

I am interested in exploring your experiences with the intervention and trail design you received in greater detail. Please feel free to be honest about what it was like for you.

General experience
1. Tell me about how the program is going for you so far.
   a. Tell me more.
   b. Probe, if needed: what do you like about the program?
      i. Tell me more.
   c. Probe, if needed: what, if anything, has been hard or challenging about the program?
   d. Probe, if needed: any side effects?
   e. Tell me more.

TQF and experience
2. Tell me specifically about how the TQF is going for you.
   a. Tell me more.
   b. Probe, if needed: has anything surprised you about the intervention?
   c. Probe, if needed: what, if anything, has been challenging about sticking to TQF?
3. Tell me about how your management of disease has changed with this program, if at all.
   a. Tell me more.
4. Tell me how your experience of taking the medications.
   a. Tell me more.
   b. Probe, if needed: how do you think about the color, smell, taste, appearance of medications?

Motivation and expectations
5. Tell me about your motivation for joining the program?
   a. Tell me more.
   b. Probe, if needed: What made you decide to sign up for this program?
6. Before participating in this program, what other ways had you tried to manage DKD or prevent progression?

a. If participant previously took part in other trials of managing DKD:
i. Tell me about how your experience with TQF compares to your prior experience with the traditional treatment.

7. In what ways has this program met or not met your expectations?
a. Tell me more.
b. Probe, if needed: has your health improved in the ways that you hoped or expected?
i. Tell me more.
c. Probe, if needed: has your renal function changed in the way you hoped or expected?
i. Tell me more.

Outcomes and sustainability

8. Tell me about your plans, if any, to stick to TQF after the program ends.
a. Tell me more.
b. If plans to stick with it, ask: why might you stick with the program?
c. If plans not to stick with it, as: why not?
d. Probe, if needed: what might be some challenges, if any, of sticking to TQF plan after the program ends?

Changes to improve the program

We would like to improve this program to help more people to prevent progression of DKD.

9. What, if anything, could have made the TQF treatment easier for you to follow?
a. Tell me more.

10. What suggestions do you have to improve this program so far?
a. Tell me more.
b. Probe, if needed: what changes would you make to help participants to better understand and follow the TQF treatment?

11. Are there particular topics that you would like to cover during the treatment period or follow-up period of the program?
a. Tell me more.

Support

We would like to understand how this program supports participants so that we can develop new ways, if needed, to help more people achieve their health and slow progression.

1. Tell me about the support you received from investigators.
a. Tell me more.
b. Probe, if needed: were they available, responsive, able to answer questions?

2. Tell me about the support you received from other staff at hospital.
a. Tell me more.

3. Was there anyone else such as a friend or family member that provided you with support during this program?
a. Tell me more.

4. Did you speak with your doctor about your participation in this program?
a. If yes: Tell me about what he or she said or advised.
i. Probe, if needed: Did you feel supported by your healthcare provider?

5. Are there ways that this program could better support you in achieving your health goals?
a. Tell me more.

Conclusion

Are there any other thoughts or experiences that you would like to share?

I want to thank you again for taking the time to discuss your thoughts and experiences. This concludes today's interview. Thank you and goodbye.

Turn off recorder

Appendix 13

Version 1.0, dated 05/07/2022

PARTICIPANT INFORMATION AND CONSENT FORM (PICF)

TANGSHEN QUSHI FORMULA FOR PEOPLE WITH STAGE 2-4 DIABETIC KIDNEY DISEASE: A PILOT RANDOMISED CONTROLLED TRIAL

RESEARCHERS:
Prof. Charlie Xue PhD, RMIT University
Prof. Tony Zhang PhD, RMIT University
Dr Yuan Ming Di PhD, RMIT University
Prof. Xusheng Liu, MD, Guangdong Provincial Hospital of Chinese Medicine (GPHCM)
Dr Lei Zhang, PhD, GPHCM
Dr Fuhua Lu, PhD, GPHCM
Dr Meifang Liu, PhD Candidate, RMIT University, GPHCM
Dr Lihong Yang, PhD, GPHCM
Dr La Zhang, PhD, GPHCM
Dr Junhui Chen, MD, GPHCM
Ms Xiaoning Xie, Nurse, GPHCM
Prof. Liping Xie, MD, Guangxi Hospital of Chinese Medicine (GXHCM)
Dr. Fang Lan, MD, GXHCM

CONTACT: Dr Meifang Liu
Phone: +86 13751728376
Email: meifangliu@gzucm.edu.cn & s3886552@student.rmit.edu.au

This Participant Information and Consent Form is 12-pages long. Please make sure you have all the pages.

PARTICIPANT INFORMATION STATEMENT

1. What is this study about?

You are invited to take part in this research study. This is because you are diagnosed with stage 2-4 diabetic kidney disease (DKD). The study is to evaluate and establish feasibility of a rigorous designed clinical trial to assess the efficacy and safety of TQF compared with placebo on kidney function for people with stage 2-4 DKD.

This Participant Information and Consent Form (PICF) tells you about the research study. It explains the tests and treatments involved. Please read this information carefully. Knowing the procedure, duration, potential risk and benefit will help you decide if you want to take part in this research. Before deciding whether or not to take part, you might want to talk about it with a relative or friend. If you have questions about anything that you don't understand or want to know more about, please ask the researchers. Participation in this research is voluntary. If you don't wish to take part, you don't have to. You are also free to leave the study at any time. If you decide you want to take part in the research study, you will be asked to sign the consent section. By signing it you are telling us that you:

- ✓ Understand what you have read;
- ✓ Consent to take part in the research study;
- ✓ Consent to have the tests and treatments that are described; and
- ✓ Consent to the use of your personal and health information as described.

You will be given a copy of this Participant Information and Consent Form to keep.

2. What is the purpose of this research?

The purpose of this research is to explore the feasibility of a rigorous designed clinical trial to assess the efficacy and safety of TQF compared with placebo on kidney function for people with stage 2-4 DKD, and explore the acceptability (and factors influencing this) of intervention and trial design.

DKD has become the leading cause of kidney failure, which accounts for about 30%~50% of kidney failure worldwide, progressing very fast and requiring renal replacement therapy, causing heavy socioeconomic and public health burden. However, the progress of DKD still cannot be delayed with the current available pharmacological therapy. There are also side effects including hypoglycaemia due to intensive treatment of hyperglycaemia are indicated in clinical trials and clinical practice.

The current western treatment options for DKD are limited, especially for delaying the progression of DKD. There is an urgent need for novel therapies. In recent years, there have been a growing number of clinical trials of Chinese herbal medicine (CHM) for DKD, indicating that CHM was associated with greater reduction of albuminuria than placebo, regardless of whether ACEi or ARBs were concurrently administered. However few studies use placebo control to evaluate the efficacy of integrated Chinese and Western medicine. Thus, further well-conducted, adequately powered trials with representative DKD populations are warranted to confirm the long-term effect of CHM, particularly on renal outcomes.

If you take part in this research, you will have a 50/50 chance of being randomly assigned to either the TQF group or the placebo group. This design allows us to compare the therapeutic effects of the TQF toe placebo and make sure changes are not just happening by chance.

This study is being conducted by the Nephrology Department at Guangdong Provincial Hospital of Chinese Medicine (GPHCM) and RMIT University. There will be approximately 60 people will take part in this study. The results of this study will be used to inform any future studies.

3. Who is running this study?

This is part of Meifang Liu's PhD project, enrolled at RMIT University. This study is being conducted at the Nephrology Department at Guangdong Provincial Hospital of Chinese Medicine (GPHCM). The research team has successively undertaken several national projects, such as special projects for public welfare industry in 2010 and 2014, National Science and Technology Support Program during the 12th Five-Year Plan period, and has accumulated rich experience in the design and implementation of multi-center clinical studies.

This study received funding from the State Key Laboratory of Dampness Syndrome of Chinese Medicine, The Second Affiliated Hospital of Guangzhou University of Chinese Medicine. Neither funder will benefit commercially from this study.

4. Who can participate in this study?

(1) If you meet with the following criteria, you will be invited to participate in this study:

1) Aged 18 years and over;
2) Confirm to the diagnostic criteria of diabetic kidney disease (see following);
3) eGFR less than 90 (excluded) mL/min/1.73m^2 and no less than 15 (included) and (calculated by CKD-EPI equation) and the change of eGFR within the latest 12 weeks before inclusion less than 30%;
4) Urinary protein creatinine ratio (PCR) less than 3500 mg/g;
5) Agree to make themselves available for the period of the study;
6) Provide written informed consent.

The diagnostic criteria of DKD is in line with American Diabetes Association (ADA)'s recommends that DKD is diagnosed based on a clear history of diabetes and a causal relationship with changes in urinary protein and renal function after excluding other primary and secondary glomerular diseases and systemic diseases [6]. Besides, diagnosis of DKD should meet at least one of the following criteria in KDIGO guideline [2]:

1) Urinary albumin-to-creatinine ratio ≥ 30 mg/g or urinary albumin excretion rate ≥ 30 mg/24h, and UACR or UAER are repeated within 3 to 6 months, and 2 out of 3 times reached or exceeded the critical value after eliminating infection and other interfering factors;
2) Estimated glomerular filtration rate (eGFR) < 60 mL/min/1.73m^2;
3) Renal biopsy was consistent with DKD pathological changes.

(2) If you have any of the following situations, you will not suitable to participate in this study since it can be dangerous for your health:

1) History of maintenance dialysis treatment or kidney transplantation for at least 90 days;
2) Plan to accept maintenance dialysis treatment or kidney transplantation within 2 weeks;
3) Diagnosed with Acute urinary tract infection within 2 weeks before randomisation;
4) Diagnosed with other primary chronic kidney diseases (such as glomerulonephritis, chronic pyelonephritis, ischemic kidney disease, etc.) and the average eGFR decreased by more than 30% within 12 weeks before enrolment;
5) History of abnormal liver function (AST or ALT or alkaline phosphatase > 3 TIMES ULN), or other known acute and chronic active hepatitis, cirrhosis;
6) History of Acute coronary syndrome, heart failure (NYHA grade III-IV [26]), or acute myocardial infarction, unstable angina, stroke, or any other cardiovascular or

cerebrovascular accident requiring revascularisation surgery within 12 weeks prior to enrolment, or a revascularisation operation is urgently needed after recruitment;

7) History of severe acute diabetic complications include but not limit to ketoacidosis, lactic acidosis, hyperotonic non-ketotic diabetic coma, hypoglycemic coma occurred within 24 weeks before screening;
8) History of active malignant tumor disease within 5 years prior to screening;
9) History of mental illness or language barrier before screening and are unable or unwilling to cooperate;
10) History of autoimmune diseases (for example, systemic lupus erythematosus) and are receiving hormone or immunosuppressive therapy before screening;
11) Be allergic to any medications or ingredients Astragalus membranaceus, Salt dodder, Blanched peach kernel, Atractylodes, Tangerine Peel, Centella asiatica, Cicada Flower; Or intolerance to these medications or ingredients (e.g. lactose intolerance);
12) Participate in other clinical trials or have participated in trials of other drugs within 12 weeks prior to screening (refer to those who are enrolled and receiving trial drugs);
13) Female during pregnancy or lactation;
14) Other conditions that the investigator judged inappropriate for the study.

5. What does participation in this research involve?

If the study is suitable for you, you will be asked to attend the study site 17 times in total, over 62 weeks. During the 48-week treatment phase of your participation in the study, you will receive either the TQF or the placebo. This is followed by 12 weeks of follow-up, without any study treatment with a final visit at the end of the study.

Visit 1: Initial Assessment: This will take approximately 45 minutes in total. It will involve 15 minutes of discussion about the study and signing of the consent form. If participant consents to the trial, they will be asked to complete 3 printed documents (this will take approximately 30 minutes). These are:

- Screening questionnaire, to check that the eligible for the study;
- Dampness Syndrome Scale of Chinese medicine; and
- Case report form (CRF).

Visit 1-2: 2-week run-in period: It is run-in period of 2 weeks prior to the treatment to collect baseline data and adjust the current treatments. Participants will be required to take medication as planned by the study design. They need to bring all the medications they are currently taking, including those for co-morbidities. During the study period, participants are not allowed to use any other oral or topical Chinese herbal medicine for the treatment of DKD. If other treatments are necessary, they will need to contact the investigators in advance. If participants plan to take other Chinese herbal medicine and not certain whether the herbs have impact on kidney function, they can consult researchers during each visit or by calling 020-81887233-30805.

Visit 2: Randomisation and Commencement of Treatment: This visit will be scheduled after the run-in period (2 weeks). The visit will take approximately 45 minutes. Participants will complete the CRF and dampness syndrome scale of Chinese medicine. The researcher will collect the blood, urine and stool specimens to measure laboratory parameters from participants. Urinary colour Doppler ultrasound and electrocardiograph are also required at this visit. Any use of Chinese medicine or Western medicine during the study is to be noted down by investigators in the CRFs. Participants will then have a 50/50 chance of being randomised to receive 48 weeks of treatment with either TQF or placebo. At this visit, participants will receive the first 4-week treatment of TQF or placebo. A 30-day take-home medication diary will be delivered to each participant along with

the prescribed medications following this visit until Visit 14. The medication should be taken twice a day, two packets for one time. Participants will be instructed on how to fill in this diary every day and take it back at the next visit. The information of left-over medications will be collected every visit during treatment period with Record Sheet of the Compliance Check.

Visits 3-13: 48-week Treatment Period: During the 48-week treatment phase, participants will attend 4-week appointments from Week 3 through to Week 50. These will last 30 minutes and are composed of: drug distribution of TQF or placebo, dampness syndrome scale of Chinese medicine completion, CRF completion, return of the previous medications together with the 30-day diary and collection of the new ones. Blood and urine samples are collected every 12 weeks (Week 2, 14, 26, 38, 50) for laboratory outcome measurement. Extra 15 minutes would be required for sample collection. If participants cannot attend hospital, the medications will be delivered to them and any left-over medication will be collected by express post.

Visit 14: End of Treatment: At the end of the 48-week treatment phase, participants will stop the TQF or placebo intervention and complete all the planned treatment. They will complete two questionnaires: 1. Credibility of Blinding Questionnaire, 2. Dampness Syndrome Scale of Chinese Medicine. Blood, urine and stool samples will be collected for outcome measures as well. The last 30-day medication diary will be collected and no more new diaries to deliver. This visit will take around 60 minutes.

Visit 15-16: 12-week Follow-Up Period: During the follow-up period, participants will be asked to paid visits every 4 weeks without TQF or placebo intervention. Investigators/researchers will follow up participants face-to-face each time to ensure participants are completing the CRFs. Each visit during this period will take 30 minutes. No laboratory outcome measures or scales for Visit 15 and Visit 16. Semi-structured interviews will be conducted with voluntary participants during this period.

Visit 17: End of Follow-Up: At the end of the 12-week follow-up phase, participants will attend one appointment (i.e. at week 62). This will last about 60 minutes and involve: CRF completion; Dampness Syndrome Scale of Chinese Medicine completion; outcome measures. Visit 17 will mark the completion of the study.

6. Payment

You will not have to pay for any aspect of this research including consultation, tests, and treatment. The test includes eGFR, urinary protein creatinine ratio, urinary albuminuria creatinine ratio, HbA1c, fasting blood-glucose, serum creatinine, blood urea nitrogen, blood uric acid, ALT, AST, AST/ALT, plasma albumin and globulin, triglyceride, cholesterol, LDL-C, HDL-C, calcium, phosphate, potassium, blood test (WBC, lymphocyte, neutrophil, monocyte, RBC, hemoglobin), Fecal routine and occult blood test, electrocardiogram and urinary colour Doppler ultrasound. Other health care not related to this study (e.g. renal biopsy) will be your own responsibility and/or that of your insurance company.

If there is any injury related to the test, the research team will pay your medical expenses and make corresponding financial compensation according to laws and policies. If you are in the placebo group, you can choose to take the TQF granule for 12 weeks at no cost after the follow-up period. You will not be paid for your participation in this research. If you have other diseases requiring other treatment or management, you will not be free of charge. If the treatment fails, we will provide other treatments such as integrative therapy of Chinese and Western medicine with your consent but not free of charge.

7. What are the possible benefits?

There is no guarantee that the TQF will assist you in delaying the progression of DKD. However, the results of this study will add to scientific knowledge and may help other people who try to prevent the progression of DKD in the future. The results may help us refine the trial protocol before testing it in large research study.

8. What are the possible risks?

During the treatment period, you need to attend the clinic and pay visits on time and do some physical and laboratory examinations, which may cause trouble or inconvenience for you. For example, there may be some discomfort when collecting blood samples. Potential symptoms may include dizziness, venous inflammation, pain, bruising, or bleeding at the puncture site. There is also a small chance of infection. Specimens will be collected for 6 times over a 62-week period.

In addition, any treatment is likely to be ineffective. DKD may progress because of ineffective treatment or other complications. This is the potential risk that every patient will face, even if they do not participate in this clinical study. During the study, if your doctor finds that the intervention adopted in this study is ineffective, your participation will be terminated. Other treatments that may be effective will be used with your consent.

9. What are the known possible side effects?

Commonly, TQF has not shown adverse reactions in clinical use and exploratory human research. Placebo is tested by some judges without adverse events. Some people can have gastrointestinal discomfort after taking these medications, such as nausea, vomiting or diarrhea. Some participants may be allergic or intolerant to the ingredients of medications. Since lactose is one of the compounds of placebo, you will be excluded from this study if you are intolerant to lactose. Mild reactions will not need to treat while the severe participants will be given appropriate treatment and stopping the intervention at once.

If you experience any adverse events or unexpected conditions or accidents, please ask your doctors for help in time. Researchers will do their best to prevent and treat any harm that may result from this study.

10. What other possible risks are there?

If these events happen, you are suggested to DKD itself may cause hyperkalemia, ketoacidosis, hypoglycemia and acute cardiovascular or cerebrovascular events (e.g. myocardial infarction, cerebral infarction), which have nothing to do with the trial interventions. If any of the above conditions occur, please report to the researcher as soon as practically possible and take corresponding measures to protect your health and safety. Researchers will record and report these events in a timely manner in accordance with the provisions.

In a medical emergency, please call 120, or go to the emergency department of the nearest hospital.

11. Conception, Pregnancy and Breast-feeding

TQF or placebo is not known to affect conception, pregnancy or breast-feeding. However, any medical intervention should be used carefully during pregnancy. Therefore, pregnant women will be excluded from the study. You are not expected to participate in this study if you trying to get pregnant. If you are confirmed to be pregnant, please inform the researchers and you will be withdrawn from the study.

12. What if new information arises during this research project?

During the research project, new information about the risks and benefits of TQF or placebo may become known to the researchers. If this occurs, you will be told about this new information and the researcher will discuss whether this new information affects you.

13. Can I have other treatments for DKD during this research study?

During the trial period, you are not allowed to receive other Chinese medicine therapies including acupuncture and moxibustion. Any other drugs that could affect urinary protein or renal function will be prohibited. This includes other oral or topical Chinese medicines that have the function of tonifying Kidney and dispelling dampness. Those formulas and patent medicine include but not limited to: Huangkui Capsule, Yishen Huazhuo Granule, Yishen Huashi Granule, Niao Du Qing Granule, Niao Du Kang Granule. If participants use any of the above-mentioned drugs, they will be withdrawn from the study.

It is important to tell the research staff about any treatments or medications you may be receiving for DKD, including over-the-counter medications, complementary or herbal remedies, acupuncture or other alternative treatments. You should also tell the research staff about any changes to these during your participation in the research. If you need additional treatment, please contact your researcher in advance. If you are not sure whether the Chinese medicine you plan to take have the function of tonifying the kidney and dispelling the dampness, you can consult the researcher during each visit or call the researcher at 020-81887233-30805. About diet or other lifestyle, we suggest you to keep a light diet, Keep warm, avoid catching cold, and exercise properly.

14. Are there alternatives to participation?

Participation in this research is not your only option. Your other options may include speaking with your doctor about new and/or different treatments or seeking out other clinical studies for participants with DKD. Discuss these options with family, friends and your doctor before deciding whether or not to take part in this study.

15. Do I have to take part in this research study?

Participation in any research study is voluntary. If you do not wish to take part, you don’t have to. If you decide to take part and later change your mind, you are free to withdraw from the study at any stage. Your decision whether to take part or not, or to take part and then withdraw, will not affect your routine treatment, your relationship with those treating you or your relationship with GPHCM. Your doctor or researcher may terminate your participation in this study at any time in your best interest.

If you decide not to participate in this research or withdraw later, you can accept other treatments like the integrative therapies. You do not need to take part in the study for the sake of DKD management.

16. What if I withdraw from this research study?

If you decide to withdraw from the research study, please notify a member of the research team before your withdrawal. This notice will allow the research team to further discuss any health risks or special requirements linked to withdrawing. You may be asked about your participation in the trial. If necessary, you may be asked to undergo laboratory tests and a physical examination. This is very beneficial for protecting your health.

If you withdraw from the study, the researchers would like to keep the personal and health information about you that have been collected. This is to help them make sure that the results of the research can be measured properly. If you do not want them to do this, you must tell them before you join the research study.

17. Could this research study be stopped unexpectedly?

This research study may be stopped for a variety of reasons. These may include reasons such as:

- Unacceptable side effects;
- The study treatment being shown not to be effective;
- The study treatment being shown to work and not need further testing.

The research team and study doctor may remove you from further participation in this study if:

- Staying in the study would be unsafe or harmful;
- You need treatment not allowed in this study;
- You fail to follow instructions;
- You become pregnant.

18. What will happen when my participation in this research study ends?

After both the treatment and follow-up period (a total 62-weeks) are completed as well as the data analysis, you could ask the researchers about results of this study if you want to know. Those who received placebo treatment may choose to take the TQF granules for a period of 12 weeks at no cost.

19. How will I be informed of the results of the research study?

After completion of the study, the data will be analysed and a detailed report will then be prepared. These results will be published in China and/or international peer-reviewed medical journals. A summary report of the study can be obtained by contacting the researchers. Researchers will send it to your email address or send as a printed document.

20. What will happen to information about me?

Any information obtained in connection with this research study that can identify you will remain confidential and will only be used for the purpose of this research study. It will only be disclosed with your permission, except as required by law. Your health records and any information obtained during the study will be stored in locked cabinets and all electronic files will be stored in a password-protected non-networked computer in a locked room at GPHCM. Access will only be by authorised personnel involved in the research.

During the study, your blood, urine and feces samples will be collected and stored in accordance with the GPHCM standard operating procedures for 10 years after the completion of the study, and the samples will be destroyed when the storage life ends. Case record forms collected at this study will be stored securely for 15 years after the completion of the study. It is possible that your medical records and biological samples may be used again in future similar studies. In which case, ethical approval will be obtained prior to the release of the materials. You have the right to request to permanently destroy the biological samples that have not been utilised/proceeded.

In any publication and/or presentation, information will be provided in such a way that you cannot be identified. Data will be presented as group data and aggregates, not data relating to specific individuals.

21. How can I access my information?

In accordance with relevant Chinese privacy and other relevant laws, you have the right to access the information collected and stored by the researchers about you. You also have the right to request that any information with which you disagree be corrected. Please contact one of the researchers named at the end of this document if you would like to access your information.

22. What happens if I am injured as a result of participating in this research study?

If you suffer an injury as a result of participation in this research study, the researchers will promptly take appropriate treatments to ensure your safety. If you claim for compensation for injuries, the research team will arrange hospital representatives to deal with the case timely and provide financial and legal support. Your claim will be handled properly together with the representative of hospital, the researcher, and the ethics committee. If all parties agree that the subject's damage is indeed related to the study, the hospital will not excuse the delay of payment for any reason.

23. Is this research study approved?

The ethical aspects of this research study has been approved by the Human Research Ethics Committee of GPHCM and filed at RMIT University. This study will be carried out according to the *Declaration of Helsinki, Guideline for Good Clinical Practice ICH E6 (R2) ICH Consensus Guideline* and *Measures for the Ethical Review of Biomedical Research Involving Humans (2016, in Chinese)*. This statement has been developed to protect the interests of people who agree to participate in human research studies.

24. Is this treatment the same or similar to the conventional treatment for DKD?

The Chinese herbal medicine used in this study is part of the combination therapy for people with stage 2-4 DKD. Usual care would be combined with TQF or placebo for all participants to delaying the progression of DKD hopefully.

25. Who can I contact?

The person you may need to contact will depend on the nature of your query. Therefore, please note the following:

For further information and appointments:
If you would like to know more about the study at any stage and ask questions related to your involvement in the study (for example, any side effects), please feel free to contact Dr Meifang Liu at +86 020-81887233-35837.
If you need urgent medical treatment, please contact your doctor or dial 120 as appropriate.

For complaints:
If you wish to contact someone, independent of the study, about ethical issues or your rights or to make a complaint, you may contact Ethics Officer, Human Research Ethics Committee, GPHCM on +86 020-81887233-35943.

26. What do I do next?

Before you decide to participate in the study, please feel free to ask any questions until you fully understand the study. It is voluntary to participate in the study. You can discuss it with your family or friends before you make a decision.

If you understand what you have read and would like to participant, please sign and return the consent form. The researcher will make all the arrangements for your study.

You will be given a signed copy of this document to keep. Please make sure you have all the pages.

Thank you, we appreciate you taking the time to read this information.

CONSENT FORM

TANGSHEN QUSHI FORMULA FOR PEOPLE WITH STAGE 2-4 DIABETIC KIDNEY DISEASE: A PILOT RANDOMISED CONTROLLED TRIAL

I have read, or have had read to me in a language that I understand, the Participant Information (version 1.0, dated dd/mm/yyyy). I have had an opportunity to ask questions and I am satisfied with the answers I have received.

I understand the potential risk and benefit of participating in this study. I am voluntary to decide whether to participate and I get sufficient time to consider it.

I understand:
- I can ask questions related to the study at any stage;
- I am free to withdraw from the study at any stage and will not be discriminated or retaliate. My withdrawal will not affect my routine treatment or relationship with those treating me or my relationship with hospital.

I understand if I withdraw from this research due to the unexpected conditions of intervention, I will inform the researcher and complete laboratory tests and physical examination. This is very beneficial for protecting my health and the whole research.

I understand that if I need additional treatment, I will contact the researcher in advance. If I have accepted additional treatment, I will tell the fact honestly.

I consent to the future use of any data/information I provide for research purposes. I understand that before they can use any data I provide, they must seek additional ethics approval. YES/ NO

I consent for other research collaborators to use any data/information I provide for future research purposes. I understand that before they can use my data, they must seek additional ethics approval. YES/NO

I also understand that the research study is strictly confidential.

I give permission for responsible individuals from this study, from regulatory authorities or from the approving Ethics Committees to have access to my records.

I understand that I will be given a signed and dated copy of this document to keep.

I freely agree to participate in this research study as described.

Name of participant/guardian/authorized trustee:

Signature: ____________________________ Date__________/_______/_______ (year/month/day)
Contact number: ______________________________

(If I am unable to sign informed consent due to lack of/limited capacity, my guardian/authorized trustee will sign for me. My relationship with him/her is ____________________.)

Name of researcher:

Signature: ____________________________ Date__________/_______/_______ (year/month/day)
Contact number: ______________________________

Contact number of GPHCM Human Research Ethics Committee: +86 020-81887233-35943.

Declaration by researcher*: I have given a verbal explanation of the research project, its procedures and risks and I believe that the participant has understood the explanation.
*A member of the research team must provide the explanation and provision of information concerning the research project.
Note: All parties signing the Consent Form must date their own signature.

Version 1.0, dated dd/mm/yyyy

PARTICIPANT INFORMATION AND CONSENT FORM (PICF)

TANGSHEN QUSHI FORMULA FOR PEOPLE WITH STAGE 2-4 DIABETIC KIDNEY DISEASE: A QUALITATIVE STUDY

RESEARCHERS:
Prof. Charlie Xue PhD, RMIT University
Prof. Tony Zhang PhD, RMIT University
Dr Yuan Ming Di PhD, RMIT University
Prof. Xusheng Liu, Chief Director, Nephrology Department, Guangdong Provincial Hospital of Chinese Medicine (GPHCM)
Dr Lei Zhang, PhD, Associate Director, GPHCM
Dr Fuhua Lu, PhD, Director, GPHCM
Dr Meifang Liu, PhD Candidate, RMIT University
Dr Lihong Yang, PhD, GPHCM
Dr La Zhang, PhD, GPHCM
Dr Junhui Chen, MD, GPHCM
Ms Xiaoning Xie, Nurse, GPHCM
Prof. Liping Xie, MD, Guangxi Hospital of Chinese Medicine (GXHCM)
Dr. Fang Lan, MD, GXHCM

CONTACT: Dr Meifang Liu
Phone: +86 13751728376
Email: meifangliu@gzucm.edu.cn & s3886552@student.rmit.edu.au

This Participant Information and Consent Form is 6-page long. Please make sure you have all the pages.

PARTICIPANT INFORMATION STATEMENT

1. What is this study about?

You are invited to take part in a research study that will explore people's opinion on the care they received as part of the study you recently participated in testing the feasibility of Tangshen Qushi Formula (TQF) for people with stage 2-4 DKD.

This Participant Information and Consent Form (PICF) tells you about the research study. It explains what will be involved. Please read this information carefully. Knowing the procedure, duration, potential risk and benefit will help you decide if you want to take part in this research. Before deciding whether or not to take part, you might want to talk about it with a relative or friend. If you have questions about anything that you don't understand or want to know more about, please ask the researchers. Participation in this research is voluntary. If you don't wish to take part, you don't have to. You are also free to leave the study at any time. If you decide you want to take part in the research study, you will be asked to sign the consent section. By signing it you are telling us that you:

- ✓ Understand what you have read;
- ✓ Consent to take part in the interview of research study; and
- ✓ Consent to the use of your personal and health information as described.

You will be given a copy of this Participant Information and Consent Form to keep.

2. What is the purpose of this research?

This research is a qualitative interview to explore participants' acceptability of the pilot trial you recently took part in. Topics of interest covered in the interview questions will be (1) perceptions of the overall trial experience; (2) acceptability of intervention components and (3) acceptability of the trial design. There will be approximately 20 people will take part in this study.

3. Who is running this study?

This is part of Meifang Liu's PhD project, enrolled at RMIT University. This study is being conducted at the Nephrology Department at Guangdong Provincial Hospital of Chinese Medicine (GPHCM). The research team has successively undertaken several national projects, such as special projects for public welfare industry in 2010 and 2014, National Science and Technology Support Program during the 12th Five-Year Plan period, and has accumulated rich experience in the design and implementation of multi-center clinical studies.
This study receives funding from the State Key Laboratory of Dampness Syndrome of Chinese Medicine, The Second Affiliated Hospital of Guangzhou University of Chinese Medicine. Neither funder will benefit commercially from this study.

4. Who can take part in the study?

You will be invited to participate in this study if you participate in the pilot trial testing the feasibility of TQF for people with stage 2-4 DKD.

5. What does participation in this research involve?

If you agree to participate in our study, we will arrange a time for you to participate in a one-on-one interview. This interview may be conducted via WeChat or videoconference (e.g. Tencent Meeting) if you cannot present in person at the trial clinic of Nephrology Department of GPHCM. The

interview will explore your opinion and acceptability on the care and design you received as part of the recent trial.

6. How much of my time will the study take?

If you decide to participate, you will need to participate in a 30-60 minute one-on-one interview. If you would like the interview to be face-to-face, there may be travel time to get to the clinic of GPHCM.

7. Do I have to take part in this research study?

Participation in any research study is voluntary. If you do not wish to take part, you don't have to. If you decide to take part and later change your mind, you are free to withdraw from the study at any stage. Your decision whether to take part or not take part, or to take part and then withdraw, will not affect your routine treatment, your relationship with those treating you or your relationship with GPHCM.

8. What if I withdraw from this research study?

If you decide to withdraw from the study, please notify a member of the research team before your withdrawal. This notice will allow the research team to further discuss any health risks or special requirements linked to withdrawing.

If you decide to leave the study, the researchers would like to keep the personal and health information about you that have been collected. This is to help them make sure that the results of the research can be measured properly. If you do not want them to do this, you must tell them before you join the research study.

9. Are there any risks or costs associated with being in the study?

Aside from giving up your time to participate in an interview, we do not expect that there will be any risks or costs associated with taking part in this study.

10. Are there any benefits associated with being in the study?

If you participate in the interview, you will be contributing to important research that helps us understand whether the new Chinese medicine we are testing is acceptable to people with stage 2-4 DKD The results may help us refine the trial protocol before testing it in definitive full-scale research study.

11. Will I be told the results of the study?

You have a right to receive feedback about the overall results of this study. You can tell us that you wish to receive feedback by leaving your email when you complete the interview. This feedback will be in the form of a one-page lay summary of the results. You will receive this feedback after the study is finished.

12. How will I be informed of the results of the research study?

After completion of the study by all participants, the results will be compiled and a detailed research report will then be prepared. These results will be published in China and/or international

peer-reviewed medical journals. A summary report of the study can also be sent to you via post or can be obtained by contacting the researchers.

13. What will happen to information about me?

Any information obtained in connection with this research study that can identify you will remain confidential and will only be used for the purpose of this research study. It will only be disclosed with your permission, except as required by law.

Your health records and any information obtained during the study will be stored in locked cabinets and all electronic files will be stored in a password-protected non-networked computer in a locked room at GPHCM. Access will only be by authorised personnel involved in the research.

After completion of the data collection and data entry processes, all files will be stored in a secure premise at GPHCM for at least 5 years and stored at RMIT for 7 years. It is possible that your medical records may be used again in future similar studies. In which case, ethical approval will be obtained prior to the release of the materials.

In any publication and/or presentation, information will be provided in such a way that you cannot be identified. Data will be presented as group data and aggregates, not data relating to specific individuals.

14. How can I access my information?

In accordance with relevant Chinese privacy and other relevant laws, you have the right to access the information collected and stored by the researchers about you. You also have the right to correct any information with which you disagree with. Please contact one of the researchers named at the end of this document if you would like to access your information.

15. Is this research study approved?

The ethical aspects of this research study has been approved by the Human Research Ethics Committee (HREC) of GPHCM and filed with HREC of RMIT University. This study will be carried out according to the *Declaration of Helsinki, Guideline for Good Clinical Practice ICH E6 (R2) ICH Consensus Guideline* and *Measures for the Ethical Review of Biomedical Research Involving Humans (2016, in Chinese)*. This statement has been developed to protect the interests of people who agree to participate in human research studies.

16. Who can I contact?

The person you may need to contact will depend on the nature of your query. Therefore, please note the following:

For further information and appointments:
If you want any further information concerning this study or have any medical problems which may be related to your involvement in the study, please feel free to contact Dr Meifang Liu at +86 020-81887233-35837.

For complaints:
If you wish to contact someone, independent of the study, about ethical issues or your rights or to make a complaint, you may contact Dr Xiaoyan Li, Ethics Officer, Human Research Ethics Committee, GPHCM on +86 020-81887233-35943.

17. What do I do next?

Before you decide to participate in the study, please feel free to ask any questions until you fully understand the study. It is voluntary to participate in the study. You can discuss it with your family or friends before you make a decision.

If you understand what you have read and would like to participant, please sign and return the consent form. The researcher will make all the arrangements for your study.

You will be given a signed copy of this document to keep. Please make sure you have all the pages.

Thank you, we appreciate you taking the time to read this information.

CONSENT FORM

TANGSHEN QUSHI FORMULA FOR PEOPLE WITH STAGE 2-4 DIABETIC KIDNEY DISEASE: A QUALITATIVE STUDY

I have read, or have had read to me in a language that I understand, the Participant Information (version 1.0, dated dd/mm/yyyy). I have had an opportunity to ask questions and I am satisfied with the answers I have received.

I understand the potential risk and benefit of participating in this study. I am voluntary to decide whether to participate and I get sufficient time to consider it.

I understand:
- I can ask questions related to the study at any stage;
- I am free to withdraw from the study at any stage and will not be discriminated or retaliated. My withdrawal will not affect my routine treatment or relationship with those treating me or my relationship with hospital.
- I understand that the interview discussion will be audio-recorded and will then be transcribed and be kept in a manner in which I cannot be identified for analysis and I agree to this.

I consent to the future use of any data/information I provide for research purposes. I understand that before they can use any data I provide, they must seek additional ethics approval. YES/ NO

I consent for other research collaborators to use any data/information I provide for future research purposes. I understand that before they can use my data, they must seek additional ethics approval. YES/NO

I also understand that the research study is strictly confidential.

I understand that I will be given a signed and dated copy of this document to keep.

I give permission for responsible individuals from this study, from regulatory authorities or from the approving Ethics Committees to have access to my records.

I hereby agree to participate in this research study.

Name of participant/guardian/authorized trustee:
Signature: ______________________________ Date___________/________/________ (year/month/day)
Contact number: ________________________________
Declaration by researcher*: If I am unable to sign informed consent due to lack of/limited capacity, my guardian/authorized trustee will sign for me. My relationship with him/her is ____________________.)

Name of researcher:
Signature: ______________________________ Date___________/________/________ (year/month/day)
Contact number: ________________________________

Contact number of GPHCM Human Research Ethics Committee: +86 020-81887233-35943.

Declaration by researcher*: I have given a verbal explanation of the research project, its procedures and risks and I believe that the participant has understood the explanation.
*A member of the research team must provide the explanation and provision of information concerning the research project.
Note: All parties signing the Consent Form must date their own signature.

Appendix 14

Version 1.0, dated 05/07/2022

CASE REPORT FORM

STUDY TITLE:

TANGSHEN QUSHI FORMULA FOR PEOPLE WITH STAGE 2-4 DIABETIC KIDNEY DISEASE: A PILOT RANDOMISED CONTROLLED TRIAL AND QUALITATIVE STUDY

Short Title (Acronym): TQF for DKD: a pilot RCT and qualitative study

**Study Site ID**	□□
**Participant ID**	□□□
**Pinyin Abbreviation of Participant**	□□□□
**Name and Signature of Investigator**	________________
**Date of Enrolment**	______/____/____
**Date of Completion**	______/____/____

Guangdong Provincial Hospital of Chinese Medicine

Case Report Form Completion Guide

1. All selected cases should fill out this form. Please read the clinical trial protocol carefully before filling out the form.

2. Please use black, blue-black pen or ballpoint pen to fill in this form.

3. The filling must be accurate and clear, and it is not allowed to be altered at will. When correcting errors, double horizontal lines should be drawn in the centre, and the initials of the modifier and the modification time should be signed. Example: ~~99.6~~ 90.6 LMF 22.01.01.

4. The initials of the patient's name should fill in four spaces. The first two letters of the two-word pinyin should be filled in for two-word names; Three-character names are filled with the first letter of the three characters and the second letter of the third character; four-character name need to fill in the first letter of each word. Example: 张红 ZHHO; 陈文燕 CWYA; 欧阳晓惠 OYXH; 李娥 LIEE.

5. During the trial period, the adverse event record form should be filled in truthfully. Time of occurrence, severity, duration, measures taken, and outcome of adverse events should be recorded. In case of serious adverse events, please report it in strict accordance with research requirements.

6. Please pay attention to the time window for the follow-up date.

7. If there is any question, please contact the Nephrology Department of Guangdong Provincial Hospital of Chinese Medicine at 020-81887233 to 35956.

8. Below are the two research sites:

Centre number	Name of organization	Centre number	Name of organization
01	Guangdong Provincial Hospital of Chinese Medicine	02	The First Affiliated Hospital of Guangxi University of Chinese Medicine (Guangxi Hospital of Chinese Medicine

 Signature of investigator:

Schedule of Enrolment, Interventions, and Assessments

Items	Time point[1] / Visit Date[2]	D0	W2	W6	W10	W14	W18	W22	W26	W30	W34	W38	W42	W46	W50	W54	W58	W62
		V1	V2	V3	V4	V5	V6	V7	V8	V9	V10	V11	V12	V13	V14	V15	V16	V17
		Date	Date	Date	Date	Date	Date	Date	Date	Date	Date	Date	Date	Date	Date	Date	Date	Date
**General items**	Participants Information and Informed Consent	√																
	Screening questionnaire	√																
	General information questionnaire	√																
	Physical examination	√	√	√	√	√	√	√	√	√	√	√	√	√	√	√	√	√
	Case report form	√	√	√	√	√	√	√	√	√	√	√	√	√	√	√	√	√
	A 30-day medication diary		√	√	√	√	√	√	√	√	√	√	√	√	√			
**Primary outcome**	eGFR slope (EPI equation)[3]		√			√			√			√			√			√
**Secondary outcomes**	Urine protein/urine creatinine		√			√			√			√			√			√
	Urine albumin/urine creatinine		√			√			√			√			√			√
	Hemoglobin A1c		√			√			√			√			√			√
	Fasting blood-glucose		√			√			√			√			√			√
	Blood lipids (triglyceride, cholesterol, low-density lipoprotein cholesterol, high-density lipoprotein cholesterol)		√			√			√			√			√			√
	Dampness Syndrome Score of Chinese Medicine		√			√			√			√			√			√
	Survival time of composite renal endpoint events[4]		√	√	√	√	√	√	√	√	√	√	√	√	√	√	√	√
**Safety outcomes**	Kidney function (serum creatinine, blood urea nitrogen, serum uric acid, total carbon dioxide)		√			√			√			√			√			√
	Liver function (alanine transaminase and aspartate		√			√			√			√			√			√

1. Participants will be asked to attend the study site 17 times over a total of 62 weeks: a run-in period of 2 weeks, 48 weeks of treatment and 12 weeks of follow-up post-treatment. "D0" means the initial day when participants enter baseline assessment; "W2" means the second week, "V1" means the first visit and so on.
2. In this table, the estimated time of each follow-up time point is provided for patients at the third row, and the date should be recorded as "yyyy-mm-dd". The actual visit time should be strictly in accordance with the schedule, if not, it should not postpone or advance more than 7 days.
3. eGFR is calculated by the Chronic Kidney Disease Epidemiology Collaboration equation, CKD-EPI.
4. If the composite renal endpoint event occurs, the Endpoint Event Laboratory Record and the Composite Renal Endpoint Event Form should be completed.

Signature of investigator:

	aminotransferase, albumin, globulin)																	
	Serum potassium/ phosphorus/calcium		√			√			√			√			√			√
	Blood/urine/stool routine test		√			√			√			√			√			√
	Electrocardiograph		√												√			
	Urinary colour Doppler ultrasound		√												√			
**Adverse event**	Adverse event/Serious adverse event	√	√	√	√	√	√	√	√	√	√	√	√	√	√	√	√	√

Signature of investigator:

Screening Questionnaire Check

Items	Complete
Screening Questionnaire	□ Yes □ No

Informed Consent Check

Items	Complete
Participant Information and Consent Form	□ Yes □ No

Random Allocation Number

(Please stick here)

Signature of investigator:

Visit 1 (Day 0)

Initial assessment (Day 0,) **Date: ___/___/_____**

Physical examination:

Heart Rate	Respiratory Rate	Blood pressure	Weight	kg	Height

Please fill in the general information separately.

Medical History

1. What is your type of diabetes?
   ☐ Type 1 diabetes ☐ Type 2 diabetes ☐ Other ____________________
2. How long have you been diagnosed with diabetes? ______________________
3. Have you performed renal biopsy? If yes, what is the result? ______________________
4. Have you ever been diagnosed, ever been told, or have you had problems with the following?
   ☐ High Blood pressure ☐ High Cholesterol/Triglycerides ☐ Kidney/Bladder problems
   ☐ Eye or vision problems ☐ Frequent nausea, vomiting, constipation, diarrhea
   ☐ Surgery in the last 5 years ☐ Heart disease/Chest pain ☐ Thyroid disease
   ☐ Asthma ☐ Numbness/pain/tingling of hands/feet
   ☐ Depression or anxiety ☐ Stroke ☐ Circulation problems
   ☐ Obesity ☐ Shortness of Breath
   ☐ Other health problems: ____
5. Do you have any allergies? ☐ No ☐ Yes: Medication/foods: ________________________
6. Do you smoke? ☐ No ☐ Have you ever smoked in the past?
   ☐ Yes: How long did you smoke for? ______________ ☐ Yes: How much? ____________
   For how long? ________________ When did you quit? ____________________________
   Have you ever tried to quit? ☐ No ☐ Yes: How long ago? _______________________

Would you like information on how to quit? ____

7. Do you drink alcohol? ☐ Yes ☐ No If "yes," amount and type? ____________________

Family History

1. List any family members with diabetes: ____
   With high blood pressure: ____
   With heart attacks or other heart problems: _______________________________________
   With stroke: _______________________ With cancer: _______________________________

Health Care Used in Past 12 months

1. When was your last physical examination? _____________________________________
2. How often do you see your regular doctor? _____________________________________
3. Have you been hospitalized within the last 12 months? ☐ Yes ☐ No
   If "yes," describe reason(s) and where: ____
4. Have you been to the emergency room within the last 12 months? ☐ Yes ☐ No
   If "yes," describe reason(s) where: ____
5. What is your eGFR within the latest 12 weeks: ______________ (CKD stage:____________)
6. What is your urinary proteinuria creatinine ratio within the latest 12 weeks: ______________
7. Have you been diagnosed with autoimmune diseases (for example, systemic lupus erythematosus) and are receiving hormone or immunosuppressive therapy before screening?

☐ Yes ☐ No

 Signature of investigator:

Have you completed the Screening Questionnaire?

□ **Yes** □ **No** If "No" is selected, please complete the questionnaire.

Are you enrolled in this trial?

□ **Yes** □ **No** If "Yes" is selected, please complete the following form.

The current Chinese medicine □ None □ Yes

If "Yes", please continue to fill in the medication name (multiple choices are allowed).

Note: Chinese patent medicines (such as Yishen Huashi Granule and Yishen Huazhuo Granule) with effect of tonifying Kidney and dispelling dampness should be avoided.

□ Huangkui capsule □ Cordyceps preparation □ Sanqi oral liquid

□ Shenyan Kangfu tablet □ Niaoduqing Granule □ Shenshuaining Tablet

□ Haikun Shenxi Capsule □ Niaodukang Granule □ Niaoduling Granule

□ Others: ________________

Western medicine treatment (please select the medicine)

1. Anti-hyperglycemic drugs: □ **None** □ **Yes**

□ Metformin

□ Acarbose

□ Non-sulfonylurea insulin secretagogue (Repaglinide/Nateglinide)

□ Sulfonylurea (Gliquidone/Glibenclamide/Glimepiride)

□ Thiazolidinedione (Pioglitazone/Rosiglitazone)

□ DPP-4 inhibitor (Sitagliptin phosphate/Repagliptin/Vigliptin)

□ SGLT-2 inhibitor (Empagliflozin/Dapagliflozin/Canagliflozin)

□ GLP-1 receptor agonist (Liraglutide)

□ Other oral hypoglycemic agents:

____

□ Insulin

2. Antihypertensive drugs: □ **None** □ **Yes**

□ ACEI/ARB drugs □ Calcium antagonist □ β receptor blockers

□ α receptor blockers □ Others: ___________

3. Diuretics: □ **None** □ **Yes**

□ Furosemide □ Spironolactone □ Torasemide □ Hydrochlorothiazide

□ Others: ___________

4. Lipid-lowering drugs: □ **None** □ **Yes**
□ Statins □ Fibrates □ Others: ____________

5. Uric acid lowering drugs: □ **None** □ **Yes**
□ Febuxostat □ Benzbromarone □ Allopurinol □ Sodium bicarbonate tablets
□ Others: ____________

6. Drugs for renal anemia: □ **None** □ **Yes**
□ Erythropoietin □ Iron □ Folic acid tablets
□ Others: _____________________________________

7. Correct the disorder of calcium and phosphorus metabolism: □ **None** □ **Yes**
□ Calcium carbonate □ Calcitriol □ Silam □ Lanthanum carbonate
□ Others: _____________________________________

8. Anticoagulant and anti-polymer drugs: □ **None** □ **Yes**
□ Aspirin □ Plavix □ Clopidogrel bisulfate
□ Others: ____________________________

9. Cardiovascular drugs: □ **None** □ **Yes**
□ Trimetazidine Hydrochloride Tablets (Wan Shuang Li)
□ Bisoprolol Fumarate (Kang Xin)
□ Others: _______________________________________

10. Other drugs: □ **None** □ **Yes**
If yes, there are: _____________________________________

Name and signature of the investigator: ____________
Date: ___/___/_____

Visit 2 (Week 2)

Note: 15 ml of venous blood sample, 10 ml of urine sample, and a stool sample will be collected from participants at this visit. Chinese medicine syndrome of dampness assessment forms are required to complete by the guidance of investigators.

Participant profile

Questions	Status	Notes
Did the participant lost to follow-up?	□ Yes □ No	If yes, please fill in the Research Completion Form.
Did any adverse event or serious adverse event occur?	□ Yes □ No	If yes, please fill in the Adverse Event Form or Serious Adverse Event Form.
Did any composite renal endpoint events (receipt of a kidney transplant, initiation of maintenance dialysis, death from kidney failure, or a sustained low GFR and a sustained percent decline in GFR) occur?	□ Yes □ No	If yes, please complete the Endpoint Event Laboratory Record and the Composite Renal Endpoint Event Form.

Physical examination data

Heart Rate	Respiratory Rate	Blood pressure	mmHg	Weight	kg

Laboratory parameters: please paste the results of following outcomes on the "Laboratory Outcome Report (stick here) (Visit 2)"

Outcomes:

eGFR (by CKD-EPI equation)
Urinary protein creatinine ratio
Urinary albuminuria creatinine ratio
HbA1c, fasting blood-glucose
Serum creatinine, blood urea nitrogen, serum uric acid, total carbon dioxide)
ALT, AST, AST/ALT, plasma albumin and globulin)
Triglyceride, cholesterol, LDL-C, HDL-C
Serum potassium/phosphorus/calcium
White blood cell, lymphocyte, neutrophil, monocyte, red blood cell, hemoglobin)
Fecal routine and occult blood test
Electrocardiogram and urinary colour ultrasound

Diagnosis of Chinese medicine syndrome (required)

1. Primary syndrome:

Please select specific syndrome with “ √ ” (single choice):

- ☐ *qi* deficiency of Spleen and Kidney
- ☐ *yang* deficiency of Spleen and Kidney
- ☐ *qi* and *yin* deficiency
- ☐ *yin* deficiency of Liver and Kidney

 Signature of investigator:

- ☐ *yin* and *yang* deficiency
- ☐ Others ___________________

2. Secondary syndrome:

(1) Dampness syndrome: □ No □ Yes

If "Yes", please select specific syndrome with " √ " (single choice):

☐ Damp-turbidity syndrome can be diagnosed by meeting any two items of ①②③④ (please select with " √ " before the numbers, multiple choices are allowed).

① Loss of appetite, or accompanied by nausea and vomiting, or accompanied by abdominal fullness, or indifferent expression, or restlessness, or itchy skin;

② Sticky mouth and smelly urine;

③ Difficult defecation, or even constipation, accompanied by frequent urination at night, or less urination;

④ Greasy tongue coating.

☐ Damp-heat syndrome can be diagnosed if any 2 items of ①②③ are met, or if any item of ①②③ plus any item of ④⑤ (please select with " √ " before the numbers, multiple choices are allowed).

① Dizziness, or soreness and weakness of waist and legs, heavy limbs, or stomach distension, or abdominal fullness, or nausea, or loss of appetite;

② Sticky mouth or sweet mouth;

③ Sticky stool, or deep-yellow urine, astringent pain in urination, or more leucorrhea, or smelly leucorrhea;

④ Red tongue with yellow greasy tongue coating;

⑤ Slippery pulse, or rolling and rapid pulse.

☐ Water-dampness syndrome can be diagnosed if any item is met (please select with " √ " before the numbers, multiple choices are allowed).

① Edema of eyelids, ankles, face, limbs and even body, or pleural effusion and ascites;

② Oliguria and anuria;

③ Slippery tongue coating;

④ Deep pulse.

- ☐ Phlegm-dampness syndrome can be diagnosed by meeting both items of ①②, or any item of them plus any one of ③④⑤ (please select with " √ " before the numbers, multiple choices are allowed).

① Chest distress, accompanied by abdominal distension, or tight throat, or unfavorable expectoration, or nausea and vomiting with much sputum;

② Obesity;

③ Dizziness, heavy head, or heavy limbs;

④ White greasy tongue coating;

⑤ Slippery pulse.

(2) Blood stasis syndrome: □ No □ Yes

Any item of ①②③④ is met can be diagnosed (please select with " √ "before the numbers, multiple choices are allowed)

① Sharp pain on fixed position, aggravated at night;

② Numbness and pain of limbs or even hemiplegia;

③ Scaly dry skin, or purple lips;

④ Purple and dark tongue, or with ecchymosis, or purple and expanded sublingual veins.

The current Chinese medicine □ None □ Yes

If "Yes", please continue to fill in the medication name (multiple choices are allowed).

Note: Chinese patent medicines (such as Yishen Huashi Granule and Yishen Huazhuo Granule) with effect of tonifying Kidney and dispelling dampness should be avoided.

□ Huangkui capsule □ Cordyceps preparation □ Sanqi oral liquid

□ Shenyan Kangfu tablet □ Niaoduqing Granule □ Shenshuaining Tablet

□ Haikun Shenxi Capsule □ Niaodukang Granule □ Niaoduling Granule

□ Others: ________________

Western medicine treatment (please select the medicine)

1. Anti-hyperglycemic drugs: □ None □ Yes

□ Metformin

□ Acarbose

□ Non-sulfonylurea insulin secretagogue (Repaglinide/Nateglinide)

□ Sulfonylurea (Gliquidone/Glibenclamide/Glimepiride)

□ Thiazolidinedione (Pioglitazone/Rosiglitazone)

□ DPP-4 inhibitor (Sitagliptin phosphate/Repagliptin/Vigliptin)

□ SGLT-2 inhibitor (Empagliflozin/Dapagliflozin/Canagliflozin)

□ GLP-1 receptor agonist (Liraglutide)

□ Other oral hypoglycemic agents:

____

□ Insulin

2. Antihypertensive drugs: □ None □ Yes

□ ACEI/ARB drugs □ Calcium antagonist □ β receptor blockers

□ α receptor blockers □ Others: ___________

3. Diuretics: □ None □ Yes

□ Furosemide □ Spironolactone □ Torasemide □ Hydrochlorothiazide

□ Others: ___________

4. Lipid-lowering drugs: □ None □ Yes

□ Statins □ Fibrates □ Others: ___________

5. Uric acid lowering drugs: □ None □ Yes

□ Febuxostat □ Benzbromarone □ Allopurinol □ Sodium bicarbonate tablets

□ Others: ___________

6. Drugs for renal anemia: □ None □ Yes

□ Erythropoietin □ Iron □ Folic acid tablets

□ Others: _________________________________

7. Correct the disorder of calcium and phosphorus metabolism: □ None □ Yes

□ Calcium carbonate □ Calcitriol □ Silam □ Lanthanum carbonate

□ Others: _________________________________

8. Anticoagulant and anti-polymer drugs: □ None □ Yes

□ Aspirin □ Plavix □ Clopidogrel bisulfate

□ Others: __________________________

9. Cardiovascular drugs: □ None □ Yes

□ Trimetazidine Hydrochloride Tablets (Wan Shuang Li)

□ Bisoprolol Fumarate (Kang Xin)

□ Others: ____

10. Other drugs: □ **None** □ **Yes**
If yes, there are:______________________________________

Have you filled in the assessment forms (Week 2) of dampness syndrome of Chinese medicine? □ **Yes** □ **No** If "No" is selected, please fill in.

Whether biological specimens have been obtained?
Blood □ Obtained □ Not Obtained
Urine □ Obtained □ Not Obtained
Feces □ Obtained □ Not Obtained

Name and signature of the investigator: ____________
Date: ___/___/____

Randomisation Allocation
(Stick here)

Signature of investigator:

RMIT Classification: Trusted

Laboratory Outcome Report (stick here)
(Visit 2)

Signature of investigator:

Visit 3 (Week 6)

Participant profile

Questions	Status	Notes
Did the participant lost to follow-up?	□ Yes □ No	If yes, please fill in the Research Completion Form.
Did any adverse event or serious adverse event occur?	□ Yes □ No	If yes, please fill in the Adverse Event Form or Serious Adverse Event Form.
Did any composite renal endpoint events (receipt of a kidney transplant, initiation of maintenance dialysis, death from kidney failure, or a sustained low GFR and a sustained percent decline in GFR) occur?	□ Yes □ No	If yes, please complete the Endpoint Event Laboratory Record and the Composite Renal Endpoint Event Form.

Physical examination data

Heart Rate		Respiratory Rate		Blood pressure	mmHg	Weight	kg

Diagnosis of Chinese medicine syndrome (required)

1. Primary syndrome:

Please select specific syndrome with “ √ ” (single choice):

- ☐ *qi* deficiency of Spleen and Kidney
- ☐ *yang* deficiency of Spleen and Kidney
- ☐ *qi* and *yin* deficiency
- ☐ *yin* deficiency of Liver and Kidney
- ☐ *yin* and *yang* deficiency
- ☐ Others ____________________

2. Secondary syndrome:

(1) Dampness syndrome: □ No □ Yes

If "Yes", please select specific syndrome with “ √ ” (single choice):

☐ Damp-turbidity syndrome can be diagnosed by meeting any two items of ①②③④ (please select with “ √ ” before the numbers, multiple choices are allowed).

① Loss of appetite, or accompanied by nausea and vomiting, or accompanied by abdominal fullness, or indifferent expression, or restlessness, or itchy skin;

② Sticky mouth and smelly urine;

③ Difficult defecation, or even constipation, accompanied by frequent urination at night, or less urination;

④ Greasy tongue coating.

 Signature of investigator:

□ Damp-heat syndrome can be diagnosed if any 2 items of ①②③ are met, or if any item of ①②③ plus any item of ④⑤ (please select with " √ " before the numbers, multiple choices are allowed).

① Dizziness, or soreness and weakness of waist and legs, heavy limbs, or stomach distension, or abdominal fullness, or nausea, or loss of appetite;

② Sticky mouth or sweet mouth;

③ Sticky stool, or deep-yellow urine, astringent pain in urination, or more leucorrhea, or smelly leucorrhea;

④ Red tongue with yellow greasy tongue coating;

⑤ Slippery pulse, or rolling and rapid pulse.

□ Water-dampness syndrome can be diagnosed if any item is met (please select with " √ " before the numbers, multiple choices are allowed).

① Edema of eyelids, ankles, face, limbs and even body, or pleural effusion and ascites;

② Oliguria and anuria;

③ Slippery tongue coating;

④ Deep pulse.

□ Phlegm-dampness syndrome can be diagnosed by meeting both items of ①②, or any item of them plus any one of ③④⑤ (please select with " √ " before the numbers, multiple choices are allowed).

① Chest distress, accompanied by abdominal distension, or tight throat, or unfavorable expectoration, or nausea and vomiting with much sputum;

② Obesity;

③ Dizziness, heavy head, or heavy limbs;

④ White greasy tongue coating;

⑤ Slippery pulse.

(2) Blood stasis syndrome: □ No □ Yes

Any item of ①②③④ is met can be diagnosed (please select with " √ "before the numbers, multiple choices are allowed)

① Sharp pain on fixed position, aggravated at night;

② Numbness and pain of limbs or even hemiplegia;

RMIT Classification: Trusted

③ Scaly dry skin, or purple lips;

④ Purple and dark tongue, or with ecchymosis, or purple and expanded sublingual veins.

The current Chinese medicine □ None □ Yes

If "Yes", please continue to fill in the medication name (multiple choices are allowed).

Note: Chinese patent medicines (such as Yishen Huashi Granule and Yishen Huazhuo Granule) with effect of tonifying Kidney and dispelling dampness should be avoided.

□ Huangkui capsule □ Cordyceps preparation □ Sanqi oral liquid

□ Shenyan Kangfu tablet □ Niaoduqing Granule □ Shenshuaining Tablet

□ Haikun Shenxi Capsule □ Niaodukang Granule □ Niaoduling Granule

□ Others: ________________

Western medicine treatment (please select the medicine)

1. Anti-hyperglycemic drugs: □ None □ Yes

□ Metformin

□ Acarbose

□ Non-sulfonylurea insulin secretagogue (Repaglinide/Nateglinide)

□ Sulfonylurea (Gliquidone/Glibenclamide/Glimepiride)

□ Thiazolidinedione (Pioglitazone/Rosiglitazone)

□ DPP-4 inhibitor (Sitagliptin phosphate/Repagliptin/Vigliptin)

□ SGLT-2 inhibitor (Empagliflozin/Dapagliflozin/Canagliflozin)

□ GLP-1 receptor agonist (Liraglutide)

□ Other oral hypoglycemic agents:

____

□ Insulin

2. Antihypertensive drugs: □ None □ Yes

□ ACEI/ARB drugs □ Calcium antagonist □ β receptor blockers

□ α receptor blockers □ Others: ___________

3. Diuretics: □ None □ Yes

□ Furosemide □ Spironolactone □ Torasemide □ Hydrochlorothiazide

□ Others: ___________

4. Lipid-lowering drugs: □ None □ Yes

□ Statins □ Fibrates □ Others: ____________

5. Uric acid lowering drugs: □ None □ Yes

□ Febuxostat □ Benzbromarone □ Allopurinol □ Sodium bicarbonate tablets

□ Others: ____________

6. Drugs for renal anemia: □ None □ Yes

□ Erythropoietin □ Iron □ Folic acid tablets

□ Others: _____________________________________

7. Correct the disorder of calcium and phosphorus metabolism: □ None □ Yes

□ Calcium carbonate □ Calcitriol □ Silam □ Lanthanum carbonate

□ Others: _____________________________________

8. Anticoagulant and anti-polymer drugs: □ None □ Yes

□ Aspirin □ Plavix □ Clopidogrel bisulfate

□ Others: ____________________________

9. Cardiovascular drugs: □ None □ Yes

□ Trimetazidine Hydrochloride Tablets (Wan Shuang Li)

□ Bisoprolol Fumarate (Kang Xin)

□ Others: _______________________________________

10. Other drugs: □ None □ Yes

If yes, there are:_____________________________________

Name and signature of the investigator: ____________

Date: ___/___/_____

Visit 4 (Week 10)

Participant profile

Questions	Status	Notes
Did the participant lost to follow-up?	□ Yes □ No	If yes, please fill in the Research Completion Form.
Did any adverse event or serious adverse event occur?	□ Yes □ No	If yes, please fill in the Adverse Event Form or Serious Adverse Event Form.
Did any composite renal endpoint events (receipt of a kidney transplant, initiation of maintenance dialysis, death from kidney failure, or a sustained low GFR and a sustained percent decline in GFR) occur?	□ Yes □ No	If yes, please complete the Endpoint Event Laboratory Record and the Composite Renal Endpoint Event Form.

Physical examination data

Heart Rate	Respiratory Rate	Blood pressure	mmHg	Weight	kg

Diagnosis of Chinese medicine syndrome (required)

1. Primary syndrome:

Please select specific syndrome with " √ " (single choice):

- ☐ *qi* deficiency of Spleen and Kidney
- ☐ *yang* deficiency of Spleen and Kidney
- ☐ *qi* and *yin* deficiency
- ☐ *yin* deficiency of Liver and Kidney
- ☐ *yin* and *yang* deficiency
- ☐ Others ___________________

2. Secondary syndrome:

(1) Dampness syndrome: □ No □ Yes

If "Yes", please select specific syndrome with " √ " (single choice):

☐ Damp-turbidity syndrome can be diagnosed by meeting any two items of ①②③④ (please select with " √ " before the numbers, multiple choices are allowed).

① Loss of appetite, or accompanied by nausea and vomiting, or accompanied by abdominal fullness, or indifferent expression, or restlessness, or itchy skin;

② Sticky mouth and smelly urine;

③ Difficult defecation, or even constipation, accompanied by frequent urination at night, or less urination;

④ Greasy tongue coating.

 Signature of investigator:

☐ Damp-heat syndrome can be diagnosed if any 2 items of ①②③ are met, or if any item of ①②③ plus any item of ④⑤ (please select with " √ " before the numbers, multiple choices are allowed).

① Dizziness, or soreness and weakness of waist and legs, heavy limbs, or stomach distension, or abdominal fullness, or nausea, or loss of appetite;

② Sticky mouth or sweet mouth;

③ Sticky stool, or deep-yellow urine, astringent pain in urination, or more leucorrhea, or smelly leucorrhea;

④ Red tongue with yellow greasy tongue coating;

⑤ Slippery pulse, or rolling and rapid pulse.

☐ Water-dampness syndrome can be diagnosed if any item is met (please select with " √ " before the numbers, multiple choices are allowed).

① Edema of eyelids, ankles, face, limbs and even body, or pleural effusion and ascites;

② Oliguria and anuria;

③ Slippery tongue coating;

④ Deep pulse.

☐ Phlegm-dampness syndrome can be diagnosed by meeting both items of ①②, or any item of them plus any one of ③④⑤ (please select with " √ " before the numbers, multiple choices are allowed).

① Chest distress, accompanied by abdominal distension, or tight throat, or unfavorable expectoration, or nausea and vomiting with much sputum;

② Obesity;

③ Dizziness, heavy head, or heavy limbs;

④ White greasy tongue coating;

⑤ Slippery pulse.

(2) Blood stasis syndrome: □ No □ Yes

Any item of ①②③④ is met can be diagnosed (please select with " √ "before the numbers, multiple choices are allowed)

① Sharp pain on fixed position, aggravated at night;

② Numbness and pain of limbs or even hemiplegia;

③ Scaly dry skin, or purple lips;

④ Purple and dark tongue, or with ecchymosis, or purple and expanded sublingual veins.

The current Chinese medicine □ None □ Yes

If "Yes", please continue to fill in the medication name (multiple choices are allowed).
Note: Chinese patent medicines (such as Yishen Huashi Granule and Yishen Huazhuo Granule) with effect of tonifying Kidney and dispelling dampness should be avoided.

□ Huangkui capsule □ Cordyceps preparation □ Sanqi oral liquid

□ Shenyan Kangfu tablet □ Niaoduqing Granule □ Shenshuaining Tablet

□ Haikun Shenxi Capsule □ Niaodukang Granule □ Niaoduling Granule

□ Others: ________________

Western medicine treatment (please select the medicine)

1. Anti-hyperglycemic drugs: □ None □ Yes

□ Metformin

□ Acarbose

□ Non-sulfonylurea insulin secretagogue (Repaglinide/Nateglinide)

□ Sulfonylurea (Gliquidone/Glibenclamide/Glimepiride)

□ Thiazolidinedione (Pioglitazone/Rosiglitazone)

□ DPP-4 inhibitor (Sitagliptin phosphate/Repagliptin/Vigliptin)

□ SGLT-2 inhibitor (Empagliflozin/Dapagliflozin/Canagliflozin)

□ GLP-1 receptor agonist (Liraglutide)

□ Other oral hypoglycemic agents:

____

□ Insulin

2. Antihypertensive drugs: □ None □ Yes

□ ACEI/ARB drugs □ Calcium antagonist □ β receptor blockers

□ α receptor blockers □ Others: ___________

3. Diuretics: □ None □ Yes

□ Furosemide □ Spironolactone □ Torasemide □ Hydrochlorothiazide

□ Others: ___________

4. Lipid-lowering drugs: □ **None** □ **Yes**

□ Statins □ Fibrates □ Others: ___________

5. Uric acid lowering drugs: □ **None** □ **Yes**

□ Febuxostat □ Benzbromarone □ Allopurinol □ Sodium bicarbonate tablets

□ Others: ___________

6. Drugs for renal anemia: □ **None** □ **Yes**

□ Erythropoietin □ Iron □ Folic acid tablets

□ Others: ________________________________

7. Correct the disorder of calcium and phosphorus metabolism: □ **None** □ **Yes**

□ Calcium carbonate □ Calcitriol □ Silam □ Lanthanum carbonate

□ Others: ________________________________

8. Anticoagulant and anti-polymer drugs: □ **None** □ **Yes**

□ Aspirin □ Plavix □ Clopidogrel bisulfate

□ Others: _________________________

9. Cardiovascular drugs: □ **None** □ **Yes**

□ Trimetazidine Hydrochloride Tablets (Wan Shuang Li)

□ Bisoprolol Fumarate (Kang Xin)

□ Others: __________________________________

10. Other drugs: □ **None** □ **Yes**

If yes, there are:________________________________

Name and signature of the investigator: ___________

Date: ___/___/_____

Visit 5 (Week 14)

Note: 15 ml of venous blood sample, 10 ml of urine sample will be collected from participants at this visit. Chinese medicine syndrome of dampness assessment forms are required to complete by the guidance of investigators.

Participant profile

Questions	Status	Notes
Did the participant lost to follow-up?	□ Yes □ No	If yes, please fill in the Research Completion Form.
Did any adverse event or serious adverse event occur?	□ Yes □ No	If yes, please fill in the Adverse Event Form or Serious Adverse Event Form.
Did any composite renal endpoint events (receipt of a kidney transplant, initiation of maintenance dialysis, death from kidney failure, or a sustained low GFR and a sustained percent decline in GFR) occur?	□ Yes □ No	If yes, please complete the Endpoint Event Laboratory Record and the Composite Renal Endpoint Event Form.

Physical examination data

Heart Rate		Respiratory Rate		Blood pressure	mmHg	Weight	kg

Laboratory parameters: please paste the results of following outcomes on the "Laboratory Outcome Report (stick here) (Visit 5)"

Outcomes:

eGFR (by CKD-EPI equation)
Urinary protein creatinine ratio
Urinary albuminuria creatinine ratio
HbA1c, fasting blood-glucose
Serum creatinine, blood urea nitrogen, serum uric acid, total carbon dioxide)
ALT, AST, AST/ALT, plasma albumin and globulin)
Triglyceride, cholesterol, LDL-C, HDL-C
Serum potassium/phosphorus/calcium
White blood cell, lymphocyte, neutrophil, monocyte, red blood cell, hemoglobin)
Fecal routine and occult blood test
Electrocardiogram and urinary colour ultrasound

Diagnosis of Chinese medicine syndrome (required)

1. Primary syndrome:

Please select specific syndrome with " √ " (single choice):

- ☐ *qi* deficiency of Spleen and Kidney
- ☐ *yang* deficiency of Spleen and Kidney
- ☐ *qi* and *yin* deficiency

Signature of investigator:

- ☐ *yin* deficiency of Liver and Kidney
- ☐ *yin* and *yang* deficiency
- ☐ Others ___________________

2. Secondary syndrome:

(1) Dampness syndrome: □ No □ Yes

If "Yes", please select specific syndrome with " √ " (single choice):

☐ Damp-turbidity syndrome can be diagnosed by meeting any two items of ①②③④ (please select with " √ " before the numbers, multiple choices are allowed).

① Loss of appetite, or accompanied by nausea and vomiting, or accompanied by abdominal fullness, or indifferent expression, or restlessness, or itchy skin;

② Sticky mouth and smelly urine;

③ Difficult defecation, or even constipation, accompanied by frequent urination at night, or less urination;

④ Greasy tongue coating.

☐ Damp-heat syndrome can be diagnosed if any 2 items of ①②③ are met, or if any item of ①②③ plus any item of ④⑤ (please select with " √ " before the numbers, multiple choices are allowed).

① Dizziness, or soreness and weakness of waist and legs, heavy limbs, or stomach distension, or abdominal fullness, or nausea, or loss of appetite;

② Sticky mouth or sweet mouth;

③ Sticky stool, or deep-yellow urine, astringent pain in urination, or more leucorrhea, or smelly leucorrhea;

④ Red tongue with yellow greasy tongue coating;

⑤ Slippery pulse, or rolling and rapid pulse.

☐ Water-dampness syndrome can be diagnosed if any item is met (please select with " √ " before the numbers, multiple choices are allowed).

① Edema of eyelids, ankles, face, limbs and even body, or pleural effusion and ascites;

② Oliguria and anuria;

③ Slippery tongue coating;

④ Deep pulse.

□ Phlegm-dampness syndrome can be diagnosed by meeting both items of ①②, or any item of them plus any one of ③④⑤ (please select with " √ " before the numbers, multiple choices are allowed).

① Chest distress, accompanied by abdominal distension, or tight throat, or unfavorable expectoration, or nausea and vomiting with much sputum;

② Obesity;

③ Dizziness, heavy head, or heavy limbs;

④ White greasy tongue coating;

⑤ Slippery pulse.

(2) Blood stasis syndrome: □ No □ Yes

Any item of ①②③④ is met can be diagnosed (please select with " √ "before the numbers, multiple choices are allowed)

① Sharp pain on fixed position, aggravated at night;

② Numbness and pain of limbs or even hemiplegia;

③ Scaly dry skin, or purple lips;

④ Purple and dark tongue, or with ecchymosis, or purple and expanded sublingual veins.

The current Chinese medicine □ None □ Yes

If "Yes", please continue to fill in the medication name (multiple choices are allowed).

Note: Chinese patent medicines (such as Yishen Huashi Granule and Yishen Huazhuo Granule) with effect of tonifying Kidney and dispelling dampness should be avoided.

□ Huangkui capsule □ Cordyceps preparation □ Sanqi oral liquid

□ Shenyan Kangfu tablet □ Niaoduqing Granule □ Shenshuaining Tablet

□ Haikun Shenxi Capsule □ Niaodukang Granule □ Niaoduling Granule

□ Others: ________________

Western medicine treatment (please select the medicine)

1. Anti-hyperglycemic drugs: □ None □ Yes

□ Metformin

□ Acarbose

□ Non-sulfonylurea insulin secretagogue (Repaglinide/Nateglinide)

□ Sulfonylurea (Gliquidone/Glibenclamide/Glimepiride)

□ Thiazolidinedione (Pioglitazone/Rosiglitazone)

□ DPP-4 inhibitor (Sitagliptin phosphate/Repagliptin/Vigliptin)

□ SGLT-2 inhibitor (Empagliflozin/Dapagliflozin/Canagliflozin)

□ GLP-1 receptor agonist (Liraglutide)

□ Other oral hypoglycemic agents:

____

□ Insulin

2. Antihypertensive drugs: □ None □ Yes

□ ACEI/ARB drugs □ Calcium antagonist □ β receptor blockers

□ α receptor blockers □ Others: ___________

3. Diuretics: □ None □ Yes

□ Furosemide □ Spironolactone □ Torasemide □ Hydrochlorothiazide

□ Others: ___________

4. Lipid-lowering drugs: □ None □ Yes

□ Statins □ Fibrates □ Others: ___________

5. Uric acid lowering drugs: □ None □ Yes

□ Febuxostat □ Benzbromarone □ Allopurinol □ Sodium bicarbonate tablets

□ Others: ___________

6. Drugs for renal anemia: □ None □ Yes

□ Erythropoietin □ Iron □ Folic acid tablets

□ Others: ________________________________

7. Correct the disorder of calcium and phosphorus metabolism: □ None □ Yes

□ Calcium carbonate □ Calcitriol □ Silam □ Lanthanum carbonate

□ Others: ________________________________

8. Anticoagulant and anti-polymer drugs: □ None □ Yes

□ Aspirin □ Plavix □ Clopidogrel bisulfate

□ Others: _________________________

9. Cardiovascular drugs: □ None □ Yes

□ Trimetazidine Hydrochloride Tablets (Wan Shuang Li)

□ Bisoprolol Fumarate (Kang Xin)
□ Others: ____

10. Other drugs: □ **None** □ **Yes**
If yes, there are:______________________________________

Have you filled in the assessment forms (Week 14) of dampness syndrome of Chinese medicine? □ **Yes** □ **No** If "No" is selected, please fill in.

Whether biological specimens have been obtained?
Blood □ Obtained □ Not Obtained
Urine □ Obtained □ Not Obtained

Name and signature of the investigator: ____________
Date: ___/___/____

Laboratory Outcome Report (stick here)
(Visit 5)

Signature of investigator:

Visit 6 (Week 18)

Participant profile

Questions	Status	Notes
Did the participant lost to follow-up?	□ Yes □ No	If yes, please fill in the Research Completion Form.
Did any adverse event or serious adverse event occur?	□ Yes □ No	If yes, please fill in the Adverse Event Form or Serious Adverse Event Form.
Did any composite renal endpoint events (receipt of a kidney transplant, initiation of maintenance dialysis, death from kidney failure, or a sustained low GFR and a sustained percent decline in GFR) occur?	□ Yes □ No	If yes, please complete the Endpoint Event Laboratory Record and the Composite Renal Endpoint Event Form.

Physical examination data

Heart Rate	Respiratory Rate	Blood pressure	mmHg	Weight	kg

Diagnosis of Chinese medicine syndrome (required)

1. Primary syndrome:

Please select specific syndrome with " √ " (single choice):

- ☐ *qi* deficiency of Spleen and Kidney
- ☐ *yang* deficiency of Spleen and Kidney
- ☐ *qi* and *yin* deficiency
- ☐ *yin* deficiency of Liver and Kidney
- ☐ *yin* and *yang* deficiency
- ☐ Others ____________________

2. Secondary syndrome:

(1) Dampness syndrome: □ No □ Yes

If "Yes", please select specific syndrome with " √ " (single choice):

☐ Damp-turbidity syndrome can be diagnosed by meeting any two items of ①②③④ (please select with " √ " before the numbers, multiple choices are allowed).

① Loss of appetite, or accompanied by nausea and vomiting, or accompanied by abdominal fullness, or indifferent expression, or restlessness, or itchy skin;

② Sticky mouth and smelly urine;

③ Difficult defecation, or even constipation, accompanied by frequent urination at night, or less urination;

④ Greasy tongue coating.

 Signature of investigator:

☐ Damp-heat syndrome can be diagnosed if any 2 items of ①②③ are met, or if any item of ①②③ plus any item of ④⑤ (please select with " √ " before the numbers, multiple choices are allowed).

① Dizziness, or soreness and weakness of waist and legs, heavy limbs, or stomach distension, or abdominal fullness, or nausea, or loss of appetite;

② Sticky mouth or sweet mouth;

③ Sticky stool, or deep-yellow urine, astringent pain in urination, or more leucorrhea, or smelly leucorrhea;

④ Red tongue with yellow greasy tongue coating;

⑤ Slippery pulse, or rolling and rapid pulse.

☐ Water-dampness syndrome can be diagnosed if any item is met (please select with " √ " before the numbers, multiple choices are allowed).

① Edema of eyelids, ankles, face, limbs and even body, or pleural effusion and ascites;

② Oliguria and anuria;

③ Slippery tongue coating;

④ Deep pulse.

☐ Phlegm-dampness syndrome can be diagnosed by meeting both items of ①②, or any item of them plus any one of ③④⑤ (please select with " √ " before the numbers, multiple choices are allowed).

① Chest distress, accompanied by abdominal distension, or tight throat, or unfavorable expectoration, or nausea and vomiting with much sputum;

② Obesity;

③ Dizziness, heavy head, or heavy limbs;

④ White greasy tongue coating;

⑤ Slippery pulse.

(2) Blood stasis syndrome: □ No □ Yes

Any item of ①②③④ is met can be diagnosed (please select with " √ "before the numbers, multiple choices are allowed)

① Sharp pain on fixed position, aggravated at night;

② Numbness and pain of limbs or even hemiplegia;

③ Scaly dry skin, or purple lips;

④ Purple and dark tongue, or with ecchymosis, or purple and expanded sublingual veins.

The current Chinese medicine □ None □ Yes

If "Yes", please continue to fill in the medication name (multiple choices are allowed).

Note: Chinese patent medicines (such as Yishen Huashi Granule and Yishen Huazhuo Granule) with effect of tonifying Kidney and dispelling dampness should be avoided.

□ Huangkui capsule □ Cordyceps preparation □ Sanqi oral liquid

□ Shenyan Kangfu tablet □ Niaoduqing Granule □ Shenshuaining Tablet

□ Haikun Shenxi Capsule □ Niaodukang Granule □ Niaoduling Granule

□ Others: ________________

Western medicine treatment (please select the medicine)

1. Anti-hyperglycemic drugs: □ None □ Yes

□ Metformin

□ Acarbose

□ Non-sulfonylurea insulin secretagogue (Repaglinide/Nateglinide)

□ Sulfonylurea (Gliquidone/Glibenclamide/Glimepiride)

□ Thiazolidinedione (Pioglitazone/Rosiglitazone)

□ DPP-4 inhibitor (Sitagliptin phosphate/Repagliptin/Vigliptin)

□ SGLT-2 inhibitor (Empagliflozin/Dapagliflozin/Canagliflozin)

□ GLP-1 receptor agonist (Liraglutide)

□ Other oral hypoglycemic agents:

____

□ Insulin

2. Antihypertensive drugs: □ None □ Yes

□ ACEI/ARB drugs □ Calcium antagonist □ β receptor blockers

□ α receptor blockers □ Others: ___________

3. Diuretics: □ None □ Yes

□ Furosemide □ Spironolactone □ Torasemide □ Hydrochlorothiazide

□ Others: ___________

4. **Lipid-lowering drugs:** □ **None** □ **Yes**

□ Statins □ Fibrates □ Others: ___________

5. Uric acid lowering drugs: □ **None** □ **Yes**

□ Febuxostat □ Benzbromarone □ Allopurinol □ Sodium bicarbonate tablets

□ Others: ___________

6. Drugs for renal anemia: □ **None** □ **Yes**

□ Erythropoietin □ Iron □ Folic acid tablets

□ Others: ________________________________

7. Correct the disorder of calcium and phosphorus metabolism: □ **None** □ **Yes**

□ Calcium carbonate □ Calcitriol □ Silam □ Lanthanum carbonate

□ Others: ________________________________

8. Anticoagulant and anti-polymer drugs: □ **None** □ **Yes**

□ Aspirin □ Plavix □ Clopidogrel bisulfate

□ Others: _________________________

9. Cardiovascular drugs: □ **None** □ **Yes**

□ Trimetazidine Hydrochloride Tablets (Wan Shuang Li)

□ Bisoprolol Fumarate (Kang Xin)

□ Others: __________________________________

10. Other drugs: □ **None** □ **Yes**

If yes, there are:________________________________

Name and signature of the investigator: ___________

Date: ___/___/____

Visit 7 (Week 22)

Participant profile

Questions	Status	Notes
Did the participant lost to follow-up?	□ Yes □ No	If yes, please fill in the Research Completion Form.
Did any adverse event or serious adverse event occur?	□ Yes □ No	If yes, please fill in the Adverse Event Form or Serious Adverse Event Form.
Did any composite renal endpoint events (receipt of a kidney transplant, initiation of maintenance dialysis, death from kidney failure, or a sustained low GFR and a sustained percent decline in GFR) occur?	□ Yes □ No	If yes, please complete the Endpoint Event Laboratory Record and the Composite Renal Endpoint Event Form.

Physical examination data

Heart Rate	Respiratory Rate	Blood pressure	mmHg	Weight	kg

Diagnosis of Chinese medicine syndrome (required)

1. Primary syndrome:

Please select specific syndrome with " √ " (single choice):

- ☐ *qi* deficiency of Spleen and Kidney
- ☐ *yang* deficiency of Spleen and Kidney
- ☐ *qi* and *yin* deficiency
- ☐ *yin* deficiency of Liver and Kidney
- ☐ *yin* and *yang* deficiency
- ☐ Others ____________________

2. Secondary syndrome:

(1) Dampness syndrome: □ No □ Yes

If "Yes", please select specific syndrome with " √ " (single choice):

☐ Damp-turbidity syndrome can be diagnosed by meeting any two items of ①②③④ (please select with " √ " before the numbers, multiple choices are allowed).

① Loss of appetite, or accompanied by nausea and vomiting, or accompanied by abdominal fullness, or indifferent expression, or restlessness, or itchy skin;

② Sticky mouth and smelly urine;

③ Difficult defecation, or even constipation, accompanied by frequent urination at night, or less urination;

④ Greasy tongue coating.

 Signature of investigator:

- ☐ Damp-heat syndrome can be diagnosed if any 2 items of ①②③ are met, or if any item of ①②③ plus any item of ④⑤ (please select with " √ " before the numbers, multiple choices are allowed).

① Dizziness, or soreness and weakness of waist and legs, heavy limbs, or stomach distension, or abdominal fullness, or nausea, or loss of appetite;

② Sticky mouth or sweet mouth;

③ Sticky stool, or deep-yellow urine, astringent pain in urination, or more leucorrhea, or smelly leucorrhea;

④ Red tongue with yellow greasy tongue coating;

⑤ Slippery pulse, or rolling and rapid pulse.

- ☐ Water-dampness syndrome can be diagnosed if any item is met (please select with " √ " before the numbers, multiple choices are allowed).

① Edema of eyelids, ankles, face, limbs and even body, or pleural effusion and ascites;

② Oliguria and anuria;

③ Slippery tongue coating;

④ Deep pulse.

- ☐ Phlegm-dampness syndrome can be diagnosed by meeting both items of ①②, or any item of them plus any one of ③④⑤ (please select with " √ " before the numbers, multiple choices are allowed).

① Chest distress, accompanied by abdominal distension, or tight throat, or unfavorable expectoration, or nausea and vomiting with much sputum;

② Obesity;

③ Dizziness, heavy head, or heavy limbs;

④ White greasy tongue coating;

⑤ Slippery pulse.

(2) Blood stasis syndrome: □ No □ Yes

Any item of ①②③④ is met can be diagnosed (please select with " √ "before the numbers, multiple choices are allowed)

① Sharp pain on fixed position, aggravated at night;

② Numbness and pain of limbs or even hemiplegia;

 Signature of investigator:

③ Scaly dry skin, or purple lips;

④ Purple and dark tongue, or with ecchymosis, or purple and expanded sublingual veins.

The current Chinese medicine □ None □ Yes

If "Yes", please continue to fill in the medication name (multiple choices are allowed).

Note: Chinese patent medicines (such as Yishen Huashi Granule and Yishen Huazhuo Granule) with effect of tonifying Kidney and dispelling dampness should be avoided.

□ Huangkui capsule □ Cordyceps preparation □ Sanqi oral liquid

□ Shenyan Kangfu tablet □ Niaoduqing Granule □ Shenshuaining Tablet

□ Haikun Shenxi Capsule □ Niaodukang Granule □ Niaoduling Granule

□ Others: ________________

Western medicine treatment (please select the medicine)

1. Anti-hyperglycemic drugs: □ None □ Yes

□ Metformin

□ Acarbose

□ Non-sulfonylurea insulin secretagogue (Repaglinide/Nateglinide)

□ Sulfonylurea (Gliquidone/Glibenclamide/Glimepiride)

□ Thiazolidinedione (Pioglitazone/Rosiglitazone)

□ DPP-4 inhibitor (Sitagliptin phosphate/Repagliptin/Vigliptin)

□ SGLT-2 inhibitor (Empagliflozin/Dapagliflozin/Canagliflozin)

□ GLP-1 receptor agonist (Liraglutide)

□ Other oral hypoglycemic agents:

____

□ Insulin

2. Antihypertensive drugs: □ None □ Yes

□ ACEI/ARB drugs □ Calcium antagonist □ β receptor blockers

□ α receptor blockers □ Others: ___________

3. Diuretics: □ None □ Yes

□ Furosemide □ Spironolactone □ Torasemide □ Hydrochlorothiazide

□ Others: ___________

 Signature of investigator:

4. Lipid-lowering drugs: □ **None** □ **Yes**

□ Statins □ Fibrates □ Others: ___________

5. Uric acid lowering drugs: □ **None** □ **Yes**

□ Febuxostat □ Benzbromarone □ Allopurinol □ Sodium bicarbonate tablets

□ Others: ___________

6. Drugs for renal anemia: □ **None** □ **Yes**

□ Erythropoietin □ Iron □ Folic acid tablets

□ Others: ________________________________

7. Correct the disorder of calcium and phosphorus metabolism: □ **None** □ **Yes**

□ Calcium carbonate □ Calcitriol □ Silam □ Lanthanum carbonate

□ Others: ________________________________

8. Anticoagulant and anti-polymer drugs: □ **None** □ **Yes**

□ Aspirin □ Plavix □ Clopidogrel bisulfate

□ Others: _________________________

9. Cardiovascular drugs: □ **None** □ **Yes**

□ Trimetazidine Hydrochloride Tablets (Wan Shuang Li)

□ Bisoprolol Fumarate (Kang Xin)

□ Others: __________________________________

10. Other drugs: □ **None** □ **Yes**

If yes, there are:________________________________

Name and signature of the investigator: ___________

Date: ___/___/____

Visit 8 (Week 26)

Note: 15 ml of venous blood sample, 10 ml of urine sample will be collected from participants at this visit. Chinese medicine syndrome of dampness assessment forms are required to complete by the guidance of investigators.

Participant profile

Questions	Status	Notes
Did the participant lost to follow-up?	□ Yes □ No	If yes, please fill in the Research Completion Form.
Did any adverse event or serious adverse event occur?	□ Yes □ No	If yes, please fill in the Adverse Event Form or Serious Adverse Event Form.
Did any composite renal endpoint events (receipt of a kidney transplant, initiation of maintenance dialysis, death from kidney failure, or a sustained low GFR and a sustained percent decline in GFR) occur?	□ Yes □ No	If yes, please complete the Endpoint Event Laboratory Record and the Composite Renal Endpoint Event Form.

Physical examination data

Heart Rate		Respiratory Rate		Blood pressure	mmHg	Weight	kg

Laboratory parameters: please paste the results of following outcomes on the "Laboratory Outcome Report (stick here) (Visit 8)"

Outcomes:

eGFR (by CKD-EPI equation)
Urinary protein creatinine ratio
Urinary albuminuria creatinine ratio
HbA1c, fasting blood-glucose
Serum creatinine, blood urea nitrogen, serum uric acid, total carbon dioxide)
ALT, AST, AST/ALT, plasma albumin and globulin)
Triglyceride, cholesterol, LDL-C, HDL-C
Serum potassium/phosphorus/calcium
White blood cell, lymphocyte, neutrophil, monocyte, red blood cell, hemoglobin)
Fecal routine and occult blood test
Electrocardiogram and urinary colour ultrasound

Diagnosis of Chinese medicine syndrome (required)

1. Primary syndrome:

Please select specific syndrome with " √ " (single choice):

- ☐ *qi* deficiency of Spleen and Kidney
- ☐ *yang* deficiency of Spleen and Kidney
- ☐ *qi* and *yin* deficiency

 Signature of investigator:

- ☐ *yin* deficiency of Liver and Kidney
- ☐ *yin* and *yang* deficiency
- ☐ Others ___________________

2. Secondary syndrome:

(1) Dampness syndrome: □ No □ Yes

If "Yes", please select specific syndrome with " √ " (single choice):

☐ Damp-turbidity syndrome can be diagnosed by meeting any two items of ①②③④ (please select with " √ " before the numbers, multiple choices are allowed).

① Loss of appetite, or accompanied by nausea and vomiting, or accompanied by abdominal fullness, or indifferent expression, or restlessness, or itchy skin;

② Sticky mouth and smelly urine;

③ Difficult defecation, or even constipation, accompanied by frequent urination at night, or less urination;

④ Greasy tongue coating.

☐ Damp-heat syndrome can be diagnosed if any 2 items of ①②③ are met, or if any item of ①②③ plus any item of ④⑤ (please select with " √ " before the numbers, multiple choices are allowed).

① Dizziness, or soreness and weakness of waist and legs, heavy limbs, or stomach distension, or abdominal fullness, or nausea, or loss of appetite;

② Sticky mouth or sweet mouth;

③ Sticky stool, or deep-yellow urine, astringent pain in urination, or more leucorrhea, or smelly leucorrhea;

④ Red tongue with yellow greasy tongue coating;

⑤ Slippery pulse, or rolling and rapid pulse.

☐ Water-dampness syndrome can be diagnosed if any item is met (please select with " √ " before the numbers, multiple choices are allowed).

① Edema of eyelids, ankles, face, limbs and even body, or pleural effusion and ascites;

② Oliguria and anuria;

③ Slippery tongue coating;

④ Deep pulse.

☐ Phlegm-dampness syndrome can be diagnosed by meeting both items of ①②, or any item of them plus any one of ③④⑤ (please select with “ √ ” before the numbers, multiple choices are allowed).

① Chest distress, accompanied by abdominal distension, or tight throat, or unfavorable expectoration, or nausea and vomiting with much sputum;

② Obesity;

③ Dizziness, heavy head, or heavy limbs;

④ White greasy tongue coating;

⑤ Slippery pulse.

(2) Blood stasis syndrome: □ No □ Yes

Any item of ①②③④ is met can be diagnosed (please select with “ √ ”before the numbers, multiple choices are allowed)

① Sharp pain on fixed position, aggravated at night;

② Numbness and pain of limbs or even hemiplegia;

③ Scaly dry skin, or purple lips;

④ Purple and dark tongue, or with ecchymosis, or purple and expanded sublingual veins.

The current Chinese medicine □ None □ Yes

If "Yes", please continue to fill in the medication name (multiple choices are allowed).

Note: Chinese patent medicines (such as Yishen Huashi Granule and Yishen Huazhuo Granule) with effect of tonifying Kidney and dispelling dampness should be avoided.

□ Huangkui capsule □ Cordyceps preparation □ Sanqi oral liquid

□ Shenyan Kangfu tablet □ Niaoduqing Granule □ Shenshuaining Tablet

□ Haikun Shenxi Capsule □ Niaodukang Granule □ Niaoduling Granule

□ Others: ________________

Western medicine treatment (please select the medicine)

1. Anti-hyperglycemic drugs: □ None □ Yes

□ Metformin

□ Acarbose

□ Non-sulfonylurea insulin secretagogue (Repaglinide/Nateglinide)

□ Sulfonylurea (Gliquidone/Glibenclamide/Glimepiride)

□ Thiazolidinedione (Pioglitazone/Rosiglitazone)

□ DPP-4 inhibitor (Sitagliptin phosphate/Repagliptin/Vigliptin)

□ SGLT-2 inhibitor (Empagliflozin/Dapagliflozin/Canagliflozin)

□ GLP-1 receptor agonist (Liraglutide)

□ Other oral hypoglycemic agents:

____

□ Insulin

2. Antihypertensive drugs: □ **None** □ **Yes**

□ ACEI/ARB drugs □ Calcium antagonist □ β receptor blockers

□ α receptor blockers □ Others: ___________

3. Diuretics: □ **None** □ **Yes**

□ Furosemide □ Spironolactone □ Torasemide □ Hydrochlorothiazide

□ Others: ___________

4. Lipid-lowering drugs: □ **None** □ **Yes**

□ Statins □ Fibrates □ Others: ___________

5. Uric acid lowering drugs: □ **None** □ **Yes**

□ Febuxostat □ Benzbromarone □ Allopurinol □ Sodium bicarbonate tablets

□ Others: ___________

6. Drugs for renal anemia: □ **None** □ **Yes**

□ Erythropoietin □ Iron □ Folic acid tablets

□ Others: ________________________________

7. Correct the disorder of calcium and phosphorus metabolism: □ **None** □ **Yes**

□ Calcium carbonate □ Calcitriol □ Silam □ Lanthanum carbonate

□ Others: ________________________________

8. Anticoagulant and anti-polymer drugs: □ **None** □ **Yes**

□ Aspirin □ Plavix □ Clopidogrel bisulfate

□ Others: _________________________

9. Cardiovascular drugs: □ **None** □ **Yes**

□ Trimetazidine Hydrochloride Tablets (Wan Shuang Li)

□ Bisoprolol Fumarate (Kang Xin)
□ Others: ___________________________________

10. Other drugs: □ **None** □ **Yes**
If yes, there are:_________________________________

Have you filled in the assessment forms (Week 26) of dampness syndrome of Chinese medicine? □ **Yes** □ **No** If "No" is selected, please fill in.

Whether biological specimens have been obtained?
Blood □ Obtained □ Not Obtained
Urine □ Obtained □ Not Obtained

Name and signature of the investigator: ___________
Date: ___/___/____

Laboratory Outcome Report (stick here)
(Visit 8)

Signature of investigator:

Visit 9 (Week 30)

Participant profile

Questions	Status	Notes
Did the participant lost to follow-up?	□ Yes □ No	If yes, please fill in the Research Completion Form.
Did any adverse event or serious adverse event occur?	□ Yes □ No	If yes, please fill in the Adverse Event Form or Serious Adverse Event Form.
Did any composite renal endpoint events (receipt of a kidney transplant, initiation of maintenance dialysis, death from kidney failure, or a sustained low GFR and a sustained percent decline in GFR) occur?	□ Yes □ No	If yes, please complete the Endpoint Event Laboratory Record and the Composite Renal Endpoint Event Form.

Physical examination data

Heart Rate	Respiratory Rate	Blood pressure	mmHg	Weight	kg

Diagnosis of Chinese medicine syndrome (required)

1. Primary syndrome:

Please select specific syndrome with " √ " (single choice):

- ☐ *qi* deficiency of Spleen and Kidney
- ☐ *yang* deficiency of Spleen and Kidney
- ☐ *qi* and *yin* deficiency
- ☐ *yin* deficiency of Liver and Kidney
- ☐ *yin* and *yang* deficiency
- ☐ Others ____________________

2. Secondary syndrome:

(1) Dampness syndrome: □ No □ Yes

If "Yes", please select specific syndrome with " √ " (single choice):

☐ Damp-turbidity syndrome can be diagnosed by meeting any two items of ①②③④ (please select with " √ " before the numbers, multiple choices are allowed).

① Loss of appetite, or accompanied by nausea and vomiting, or accompanied by abdominal fullness, or indifferent expression, or restlessness, or itchy skin;

② Sticky mouth and smelly urine;

③ Difficult defecation, or even constipation, accompanied by frequent urination at night, or less urination;

④ Greasy tongue coating.

 Signature of investigator:

☐ Damp-heat syndrome can be diagnosed if any 2 items of ①②③ are met, or if any item of ①②③ plus any item of ④⑤ (please select with " √ " before the numbers, multiple choices are allowed).

① Dizziness, or soreness and weakness of waist and legs, heavy limbs, or stomach distension, or abdominal fullness, or nausea, or loss of appetite;

② Sticky mouth or sweet mouth;

③ Sticky stool, or deep-yellow urine, astringent pain in urination, or more leucorrhea, or smelly leucorrhea;

④ Red tongue with yellow greasy tongue coating;

⑤ Slippery pulse, or rolling and rapid pulse.

☐ Water-dampness syndrome can be diagnosed if any item is met (please select with " √ " before the numbers, multiple choices are allowed).

① Edema of eyelids, ankles, face, limbs and even body, or pleural effusion and ascites;

② Oliguria and anuria;

③ Slippery tongue coating;

④ Deep pulse.

☐ Phlegm-dampness syndrome can be diagnosed by meeting both items of ①②, or any item of them plus any one of ③④⑤ (please select with " √ " before the numbers, multiple choices are allowed).

① Chest distress, accompanied by abdominal distension, or tight throat, or unfavorable expectoration, or nausea and vomiting with much sputum;

② Obesity;

③ Dizziness, heavy head, or heavy limbs;

④ White greasy tongue coating;

⑤ Slippery pulse.

(2) Blood stasis syndrome: □ No □ Yes

Any item of ①②③④ is met can be diagnosed (please select with " √ "before the numbers, multiple choices are allowed)

① Sharp pain on fixed position, aggravated at night;

② Numbness and pain of limbs or even hemiplegia;

③ Scaly dry skin, or purple lips;

④ Purple and dark tongue, or with ecchymosis, or purple and expanded sublingual veins.

The current Chinese medicine □ None □ Yes

If "Yes", please continue to fill in the medication name (multiple choices are allowed).

Note: Chinese patent medicines (such as Yishen Huashi Granule and Yishen Huazhuo Granule) with effect of tonifying Kidney and dispelling dampness should be avoided.

□ Huangkui capsule □ Cordyceps preparation □ Sanqi oral liquid

□ Shenyan Kangfu tablet □ Niaoduqing Granule □ Shenshuaining Tablet

□ Haikun Shenxi Capsule □ Niaodukang Granule □ Niaoduling Granule

□ Others: ________________

Western medicine treatment (please select the medicine)

1. Anti-hyperglycemic drugs: □ None □ Yes

□ Metformin

□ Acarbose

□ Non-sulfonylurea insulin secretagogue (Repaglinide/Nateglinide)

□ Sulfonylurea (Gliquidone/Glibenclamide/Glimepiride)

□ Thiazolidinedione (Pioglitazone/Rosiglitazone)

□ DPP-4 inhibitor (Sitagliptin phosphate/Repagliptin/Vigliptin)

□ SGLT-2 inhibitor (Empagliflozin/Dapagliflozin/Canagliflozin)

□ GLP-1 receptor agonist (Liraglutide)

□ Other oral hypoglycemic agents:

____

□ Insulin

2. Antihypertensive drugs: □ None □ Yes

□ ACEI/ARB drugs □ Calcium antagonist □ β receptor blockers

□ α receptor blockers □ Others: ___________

3. Diuretics: □ None □ Yes

□ Furosemide □ Spironolactone □ Torasemide □ Hydrochlorothiazide

□ Others: ___________

 Signature of investigator:

4. Lipid-lowering drugs: □ **None** □ **Yes**

□ Statins □ Fibrates □ Others: ___________

5. Uric acid lowering drugs: □ **None** □ **Yes**

□ Febuxostat □ Benzbromarone □ Allopurinol □ Sodium bicarbonate tablets

□ Others: ___________

6. Drugs for renal anemia: □ **None** □ **Yes**

□ Erythropoietin □ Iron □ Folic acid tablets

□ Others: ________________________________

7. Correct the disorder of calcium and phosphorus metabolism: □ **None** □ **Yes**

□ Calcium carbonate □ Calcitriol □ Silam □ Lanthanum carbonate

□ Others: ________________________________

8. Anticoagulant and anti-polymer drugs: □ **None** □ **Yes**

□ Aspirin □ Plavix □ Clopidogrel bisulfate

□ Others: _________________________

9. Cardiovascular drugs: □ **None** □ **Yes**

□ Trimetazidine Hydrochloride Tablets (Wan Shuang Li)

□ Bisoprolol Fumarate (Kang Xin)

□ Others: __________________________________

10. Other drugs: □ **None** □ **Yes**

If yes, there are:________________________________

Name and signature of the investigator: ___________

Date: ___/___/____

Visit 10 (Week 34)

Participant profile

Questions	Status	Notes
Did the participant lost to follow-up?	□ Yes □ No	If yes, please fill in the Research Completion Form.
Did any adverse event or serious adverse event occur?	□ Yes □ No	If yes, please fill in the Adverse Event Form or Serious Adverse Event Form.
Did any composite renal endpoint events (receipt of a kidney transplant, initiation of maintenance dialysis, death from kidney failure, or a sustained low GFR and a sustained percent decline in GFR) occur?	□ Yes □ No	If yes, please complete the Endpoint Event Laboratory Record and the Composite Renal Endpoint Event Form.

Physical examination data

Heart Rate	Respiratory Rate	Blood pressure	mmHg	Weight	kg

Diagnosis of Chinese medicine syndrome (required)

1. Primary syndrome:

Please select specific syndrome with " √ " (single choice):

- ☐ *qi* deficiency of Spleen and Kidney
- ☐ *yang* deficiency of Spleen and Kidney
- ☐ *qi* and *yin* deficiency
- ☐ *yin* deficiency of Liver and Kidney
- ☐ *yin* and *yang* deficiency
- ☐ Others ____________________

2. Secondary syndrome:

(1) Dampness syndrome: □ No □ Yes

If "Yes", please select specific syndrome with " √ " (single choice):

☐ Damp-turbidity syndrome can be diagnosed by meeting any two items of ①②③④ (please select with " √ " before the numbers, multiple choices are allowed).

① Loss of appetite, or accompanied by nausea and vomiting, or accompanied by abdominal fullness, or indifferent expression, or restlessness, or itchy skin;

② Sticky mouth and smelly urine;

③ Difficult defecation, or even constipation, accompanied by frequent urination at night, or less urination;

④ Greasy tongue coating.

 Signature of investigator:

☐ Damp-heat syndrome can be diagnosed if any 2 items of ①②③ are met, or if any item of ①②③ plus any item of ④⑤ (please select with " √ " before the numbers, multiple choices are allowed).

① Dizziness, or soreness and weakness of waist and legs, heavy limbs, or stomach distension, or abdominal fullness, or nausea, or loss of appetite;

② Sticky mouth or sweet mouth;

③ Sticky stool, or deep-yellow urine, astringent pain in urination, or more leucorrhea, or smelly leucorrhea;

④ Red tongue with yellow greasy tongue coating;

⑤ Slippery pulse, or rolling and rapid pulse.

☐ Water-dampness syndrome can be diagnosed if any item is met (please select with " √ " before the numbers, multiple choices are allowed).

① Edema of eyelids, ankles, face, limbs and even body, or pleural effusion and ascites;

② Oliguria and anuria;

③ Slippery tongue coating;

④ Deep pulse.

☐ Phlegm-dampness syndrome can be diagnosed by meeting both items of ①②, or any item of them plus any one of ③④⑤ (please select with " √ " before the numbers, multiple choices are allowed).

① Chest distress, accompanied by abdominal distension, or tight throat, or unfavorable expectoration, or nausea and vomiting with much sputum;

② Obesity;

③ Dizziness, heavy head, or heavy limbs;

④ White greasy tongue coating;

⑤ Slippery pulse.

(2) Blood stasis syndrome: □ No □ Yes

Any item of ①②③④ is met can be diagnosed (please select with " √ "before the numbers, multiple choices are allowed)

① Sharp pain on fixed position, aggravated at night;

② Numbness and pain of limbs or even hemiplegia;

③ Scaly dry skin, or purple lips;

④ Purple and dark tongue, or with ecchymosis, or purple and expanded sublingual veins.

The current Chinese medicine □ None □ Yes

If "Yes", please continue to fill in the medication name (multiple choices are allowed).

Note: Chinese patent medicines (such as Yishen Huashi Granule and Yishen Huazhuo Granule) with effect of tonifying Kidney and dispelling dampness should be avoided.

□ Huangkui capsule □ Cordyceps preparation □ Sanqi oral liquid

□ Shenyan Kangfu tablet □ Niaoduqing Granule □ Shenshuaining Tablet

□ Haikun Shenxi Capsule □ Niaodukang Granule □ Niaoduling Granule

□ Others: ________________

Western medicine treatment (please select the medicine)

1. Anti-hyperglycemic drugs: □ None □ Yes

□ Metformin

□ Acarbose

□ Non-sulfonylurea insulin secretagogue (Repaglinide/Nateglinide)

□ Sulfonylurea (Gliquidone/Glibenclamide/Glimepiride)

□ Thiazolidinedione (Pioglitazone/Rosiglitazone)

□ DPP-4 inhibitor (Sitagliptin phosphate/Repagliptin/Vigliptin)

□ SGLT-2 inhibitor (Empagliflozin/Dapagliflozin/Canagliflozin)

□ GLP-1 receptor agonist (Liraglutide)

□ Other oral hypoglycemic agents:

____

□ Insulin

2. Antihypertensive drugs: □ None □ Yes

□ ACEI/ARB drugs □ Calcium antagonist □ β receptor blockers

□ α receptor blockers □ Others: ___________

3. Diuretics: □ None □ Yes

□ Furosemide □ Spironolactone □ Torasemide □ Hydrochlorothiazide

□ Others: ___________

4. Lipid-lowering drugs: □ None □ Yes

□ Statins □ Fibrates □ Others: ___________

5. Uric acid lowering drugs: □ None □ Yes

□ Febuxostat □ Benzbromarone □ Allopurinol □ Sodium bicarbonate tablets

□ Others: ___________

6. Drugs for renal anemia: □ None □ Yes

□ Erythropoietin □ Iron □ Folic acid tablets

□ Others: ________________________________

7. Correct the disorder of calcium and phosphorus metabolism: □ None □ Yes

□ Calcium carbonate □ Calcitriol □ Silam □ Lanthanum carbonate

□ Others: ________________________________

8. Anticoagulant and anti-polymer drugs: □ None □ Yes

□ Aspirin □ Plavix □ Clopidogrel bisulfate

□ Others: _________________________

9. Cardiovascular drugs: □ None □ Yes

□ Trimetazidine Hydrochloride Tablets (Wan Shuang Li)

□ Bisoprolol Fumarate (Kang Xin)

□ Others: __________________________________

10. Other drugs: □ None □ Yes

If yes, there are:________________________________

Name and signature of the investigator: ___________

Date: ___/___/____

Visit 11 (Week 38)

Note: 15 ml of venous blood sample, 10 ml of urine sample will be collected from participants at this visit. Chinese medicine syndrome of dampness assessment forms are required to complete by the guidance of investigators.

Participant profile

Questions	Status	Notes
Did the participant lost to follow-up?	□ Yes □ No	If yes, please fill in the Research Completion Form.
Did any adverse event or serious adverse event occur?	□ Yes □ No	If yes, please fill in the Adverse Event Form or Serious Adverse Event Form.
Did any composite renal endpoint events (receipt of a kidney transplant, initiation of maintenance dialysis, death from kidney failure, or a sustained low GFR and a sustained percent decline in GFR) occur?	□ Yes □ No	If yes, please complete the Endpoint Event Laboratory Record and the Composite Renal Endpoint Event Form.

Physical examination data

Heart Rate	Respiratory Rate	Blood pressure	mmHg	Weight	kg

Laboratory parameters: please paste the results of following outcomes on the "Laboratory Outcome Report (stick here) (Visit 11)"

Outcomes:

eGFR (by CKD-EPI equation)
Urinary protein creatinine ratio
Urinary albuminuria creatinine ratio
HbA1c, fasting blood-glucose
Serum creatinine, blood urea nitrogen, serum uric acid, total carbon dioxide)
ALT, AST, AST/ALT, plasma albumin and globulin)
Triglyceride, cholesterol, LDL-C, HDL-C
Serum potassium/phosphorus/calcium
White blood cell, lymphocyte, neutrophil, monocyte, red blood cell, hemoglobin)
Fecal routine and occult blood test
Electrocardiogram and urinary colour ultrasound

Diagnosis of Chinese medicine syndrome (required)

1. Primary syndrome:

Please select specific syndrome with " √ " (single choice):

- ☐ *qi* deficiency of Spleen and Kidney
- ☐ *yang* deficiency of Spleen and Kidney
- ☐ *qi* and *yin* deficiency

 Signature of investigator:

RMIT Classification: Trusted

- ☐ *yin* deficiency of Liver and Kidney
- ☐ *yin* and *yang* deficiency
- ☐ Others ___________________

2. Secondary syndrome:

(1) Dampness syndrome: □ No □ Yes

If "Yes", please select specific syndrome with " √ " (single choice):

☐ Damp-turbidity syndrome can be diagnosed by meeting any two items of ①②③④ (please select with " √ " before the numbers, multiple choices are allowed).

① Loss of appetite, or accompanied by nausea and vomiting, or accompanied by abdominal fullness, or indifferent expression, or restlessness, or itchy skin;

② Sticky mouth and smelly urine;

③ Difficult defecation, or even constipation, accompanied by frequent urination at night, or less urination;

④ Greasy tongue coating.

☐ Damp-heat syndrome can be diagnosed if any 2 items of ①②③ are met, or if any item of ①②③ plus any item of ④⑤ (please select with " √ " before the numbers, multiple choices are allowed).

① Dizziness, or soreness and weakness of waist and legs, heavy limbs, or stomach distension, or abdominal fullness, or nausea, or loss of appetite;

② Sticky mouth or sweet mouth;

③ Sticky stool, or deep-yellow urine, astringent pain in urination, or more leucorrhea, or smelly leucorrhea;

④ Red tongue with yellow greasy tongue coating;

⑤ Slippery pulse, or rolling and rapid pulse.

☐ Water-dampness syndrome can be diagnosed if any item is met (please select with " √ " before the numbers, multiple choices are allowed).

① Edema of eyelids, ankles, face, limbs and even body, or pleural effusion and ascites;

② Oliguria and anuria;

③ Slippery tongue coating;

④ Deep pulse.

□ Phlegm-dampness syndrome can be diagnosed by meeting both items of ①②, or any item of them plus any one of ③④⑤ (please select with " √ " before the numbers, multiple choices are allowed).

① Chest distress, accompanied by abdominal distension, or tight throat, or unfavorable expectoration, or nausea and vomiting with much sputum;

② Obesity;

③ Dizziness, heavy head, or heavy limbs;

④ White greasy tongue coating;

⑤ Slippery pulse.

(2) Blood stasis syndrome: □ No □ Yes

Any item of ①②③④ is met can be diagnosed (please select with " √ "before the numbers, multiple choices are allowed)

① Sharp pain on fixed position, aggravated at night;

② Numbness and pain of limbs or even hemiplegia;

③ Scaly dry skin, or purple lips;

④ Purple and dark tongue, or with ecchymosis, or purple and expanded sublingual veins.

The current Chinese medicine □ None □ Yes

If "Yes", please continue to fill in the medication name (multiple choices are allowed).

Note: Chinese patent medicines (such as Yishen Huashi Granule and Yishen Huazhuo Granule) with effect of tonifying Kidney and dispelling dampness should be avoided.

□ Huangkui capsule □ Cordyceps preparation □ Sanqi oral liquid

□ Shenyan Kangfu tablet □ Niaoduqing Granule □ Shenshuaining Tablet

□ Haikun Shenxi Capsule □ Niaodukang Granule □ Niaoduling Granule

□ Others: ________________

Western medicine treatment (please select the medicine)

1. Anti-hyperglycemic drugs: □ None □ Yes

□ Metformin

□ Acarbose

□ Non-sulfonylurea insulin secretagogue (Repaglinide/Nateglinide)

□ Sulfonylurea (Gliquidone/Glibenclamide/Glimepiride)

□ Thiazolidinedione (Pioglitazone/Rosiglitazone)

□ DPP-4 inhibitor (Sitagliptin phosphate/Repagliptin/Vigliptin)

□ SGLT-2 inhibitor (Empagliflozin/Dapagliflozin/Canagliflozin)

□ GLP-1 receptor agonist (Liraglutide)

□ Other oral hypoglycemic agents:

____

□ Insulin

2. Antihypertensive drugs: □ **None** □ **Yes**

□ ACEI/ARB drugs □ Calcium antagonist □ β receptor blockers

□ α receptor blockers □ Others: ___________

3. Diuretics: □ **None** □ **Yes**

□ Furosemide □ Spironolactone □ Torasemide □ Hydrochlorothiazide

□ Others: ___________

4. Lipid-lowering drugs: □ **None** □ **Yes**

□ Statins □ Fibrates □ Others: ___________

5. Uric acid lowering drugs: □ **None** □ **Yes**

□ Febuxostat □ Benzbromarone □ Allopurinol □ Sodium bicarbonate tablets

□ Others: ___________

6. Drugs for renal anemia: □ **None** □ **Yes**

□ Erythropoietin □ Iron □ Folic acid tablets

□ Others: ____________________________________

7. Correct the disorder of calcium and phosphorus metabolism: □ **None** □ **Yes**

□ Calcium carbonate □ Calcitriol □ Silam □ Lanthanum carbonate

□ Others: ____________________________________

8. Anticoagulant and anti-polymer drugs: □ **None** □ **Yes**

□ Aspirin □ Plavix □ Clopidogrel bisulfate

□ Others: ____________________________

9. Cardiovascular drugs: □ **None** □ **Yes**

□ Trimetazidine Hydrochloride Tablets (Wan Shuang Li)

□ Bisoprolol Fumarate (Kang Xin)
□ Others: ____

10. Other drugs: □ **None** □ **Yes**
If yes, there are:______________________________________

Have you filled in the assessment forms (Week 38) of dampness syndrome of Chinese medicine? □ **Yes** □ **No** If "No" is selected, please fill in.

Whether biological specimens have been obtained?
Blood □ Obtained □ Not Obtained
Urine □ Obtained □ Not Obtained

Name and signature of the investigator: ____________
Date: ___/___/____

Laboratory Outcome Report (stick here)
(Visit 11)

Signature of investigator:

Visit 12 (Week 42)

Participant profile

Questions	Status	Notes
Did the participant lost to follow-up?	□ Yes □ No	If yes, please fill in the Research Completion Form.
Did any adverse event or serious adverse event occur?	□ Yes □ No	If yes, please fill in the Adverse Event Form or Serious Adverse Event Form.
Did any composite renal endpoint events (receipt of a kidney transplant, initiation of maintenance dialysis, death from kidney failure, or a sustained low GFR and a sustained percent decline in GFR) occur?	□ Yes □ No	If yes, please complete the Endpoint Event Laboratory Record and the Composite Renal Endpoint Event Form.

Physical examination data

Heart Rate	Respiratory Rate	Blood pressure	mmHg	Weight	kg

Diagnosis of Chinese medicine syndrome (required)

1. Primary syndrome:

Please select specific syndrome with “ √ ” (single choice):

- ☐ *qi* deficiency of Spleen and Kidney
- ☐ *yang* deficiency of Spleen and Kidney
- ☐ *qi* and *yin* deficiency
- ☐ *yin* deficiency of Liver and Kidney
- ☐ *yin* and *yang* deficiency
- ☐ Others ____________________

2. Secondary syndrome:

(1) Dampness syndrome: □ No □ Yes

If "Yes", please select specific syndrome with “ √ ” (single choice):

☐ Damp-turbidity syndrome can be diagnosed by meeting any two items of ①②③④ (please select with “ √ ” before the numbers, multiple choices are allowed).

① Loss of appetite, or accompanied by nausea and vomiting, or accompanied by abdominal fullness, or indifferent expression, or restlessness, or itchy skin;

② Sticky mouth and smelly urine;

③ Difficult defecation, or even constipation, accompanied by frequent urination at night, or less urination;

④ Greasy tongue coating.

Signature of investigator:

☐ Damp-heat syndrome can be diagnosed if any 2 items of ①②③ are met, or if any item of ①②③ plus any item of ④⑤ (please select with " √ " before the numbers, multiple choices are allowed).

① Dizziness, or soreness and weakness of waist and legs, heavy limbs, or stomach distension, or abdominal fullness, or nausea, or loss of appetite;

② Sticky mouth or sweet mouth;

③ Sticky stool, or deep-yellow urine, astringent pain in urination, or more leucorrhea, or smelly leucorrhea;

④ Red tongue with yellow greasy tongue coating;

⑤ Slippery pulse, or rolling and rapid pulse.

☐ Water-dampness syndrome can be diagnosed if any item is met (please select with " √ " before the numbers, multiple choices are allowed).

① Edema of eyelids, ankles, face, limbs and even body, or pleural effusion and ascites;

② Oliguria and anuria;

③ Slippery tongue coating;

④ Deep pulse.

☐ Phlegm-dampness syndrome can be diagnosed by meeting both items of ①②, or any item of them plus any one of ③④⑤ (please select with " √ " before the numbers, multiple choices are allowed).

① Chest distress, accompanied by abdominal distension, or tight throat, or unfavorable expectoration, or nausea and vomiting with much sputum;

② Obesity;

③ Dizziness, heavy head, or heavy limbs;

④ White greasy tongue coating;

⑤ Slippery pulse.

(2) Blood stasis syndrome: □ No □ Yes

Any item of ①②③④ is met can be diagnosed (please select with " √ "before the numbers, multiple choices are allowed)

① Sharp pain on fixed position, aggravated at night;

② Numbness and pain of limbs or even hemiplegia;

 Signature of investigator:

③ Scaly dry skin, or purple lips;

④ Purple and dark tongue, or with ecchymosis, or purple and expanded sublingual veins.

The current Chinese medicine □ None □ Yes

If "Yes", please continue to fill in the medication name (multiple choices are allowed).

Note: Chinese patent medicines (such as Yishen Huashi Granule and Yishen Huazhuo Granule) with effect of tonifying Kidney and dispelling dampness should be avoided.

□ Huangkui capsule □ Cordyceps preparation □ Sanqi oral liquid

□ Shenyan Kangfu tablet □ Niaoduqing Granule □ Shenshuaining Tablet

□ Haikun Shenxi Capsule □ Niaodukang Granule □ Niaoduling Granule

□ Others: ________________

Western medicine treatment (please select the medicine)

1. Anti-hyperglycemic drugs: □ None □ Yes

□ Metformin

□ Acarbose

□ Non-sulfonylurea insulin secretagogue (Repaglinide/Nateglinide)

□ Sulfonylurea (Gliquidone/Glibenclamide/Glimepiride)

□ Thiazolidinedione (Pioglitazone/Rosiglitazone)

□ DPP-4 inhibitor (Sitagliptin phosphate/Repagliptin/Vigliptin)

□ SGLT-2 inhibitor (Empagliflozin/Dapagliflozin/Canagliflozin)

□ GLP-1 receptor agonist (Liraglutide)

□ Other oral hypoglycemic agents:

____

□ Insulin

2. Antihypertensive drugs: □ None □ Yes

□ ACEI/ARB drugs □ Calcium antagonist □ β receptor blockers

□ α receptor blockers □ Others: ___________

3. Diuretics: □ None □ Yes

□ Furosemide □ Spironolactone □ Torasemide □ Hydrochlorothiazide

□ Others: ___________

4. Lipid-lowering drugs: □ **None** □ **Yes**

□ Statins □ Fibrates □ Others: ___________

5. Uric acid lowering drugs: □ **None** □ **Yes**

□ Febuxostat □ Benzbromarone □ Allopurinol □ Sodium bicarbonate tablets

□ Others: ___________

6. Drugs for renal anemia: □ **None** □ **Yes**

□ Erythropoietin □ Iron □ Folic acid tablets

□ Others: __________________________________

7. Correct the disorder of calcium and phosphorus metabolism: □ **None** □ **Yes**

□ Calcium carbonate □ Calcitriol □ Silam □ Lanthanum carbonate

□ Others: __________________________________

8. Anticoagulant and anti-polymer drugs: □ **None** □ **Yes**

□ Aspirin □ Plavix □ Clopidogrel bisulfate

□ Others: __________________________

9. Cardiovascular drugs: □ **None** □ **Yes**

□ Trimetazidine Hydrochloride Tablets (Wan Shuang Li)

□ Bisoprolol Fumarate (Kang Xin)

□ Others: ____________________________________

10. Other drugs: □ **None** □ **Yes**

If yes, there are:__________________________________

Name and signature of the investigator: ___________

Date: ___/___/____

RMIT Classification: Trusted

Visit 13 (Week 46)

Participant profile

Questions	Status	Notes
Did the participant lost to follow-up?	□ Yes □ No	If yes, please fill in the Research Completion Form.
Did any adverse event or serious adverse event occur?	□ Yes □ No	If yes, please fill in the Adverse Event Form or Serious Adverse Event Form.
Did any composite renal endpoint events (receipt of a kidney transplant, initiation of maintenance dialysis, death from kidney failure, or a sustained low GFR and a sustained percent decline in GFR) occur?	□ Yes □ No	If yes, please complete the Endpoint Event Laboratory Record and the Composite Renal Endpoint Event Form.

Physical examination data

Heart Rate	Respiratory Rate	Blood pressure	mmHg	Weight	kg

Diagnosis of Chinese medicine syndrome (required)

1. Primary syndrome:

Please select specific syndrome with “ √ ” (single choice):

- ☐ *qi* deficiency of Spleen and Kidney
- ☐ *yang* deficiency of Spleen and Kidney
- ☐ *qi* and *yin* deficiency
- ☐ *yin* deficiency of Liver and Kidney
- ☐ *yin* and *yang* deficiency
- ☐ Others ____________________

2. Secondary syndrome:

(1) Dampness syndrome: □ No □ Yes

If "Yes", please select specific syndrome with “ √ ” (single choice):

☐ Damp-turbidity syndrome can be diagnosed by meeting any two items of ①②③④ (please select with “ √ ” before the numbers, multiple choices are allowed).

① Loss of appetite, or accompanied by nausea and vomiting, or accompanied by abdominal fullness, or indifferent expression, or restlessness, or itchy skin;

② Sticky mouth and smelly urine;

③ Difficult defecation, or even constipation, accompanied by frequent urination at night, or less urination;

④ Greasy tongue coating.

 Signature of investigator:

□ Damp-heat syndrome can be diagnosed if any 2 items of ①②③ are met, or if any item of ①②③ plus any item of ④⑤ (please select with " √ " before the numbers, multiple choices are allowed).

① Dizziness, or soreness and weakness of waist and legs, heavy limbs, or stomach distension, or abdominal fullness, or nausea, or loss of appetite;

② Sticky mouth or sweet mouth;

③ Sticky stool, or deep-yellow urine, astringent pain in urination, or more leucorrhea, or smelly leucorrhea;

④ Red tongue with yellow greasy tongue coating;

⑤ Slippery pulse, or rolling and rapid pulse.

□ Water-dampness syndrome can be diagnosed if any item is met (please select with " √ " before the numbers, multiple choices are allowed).

① Edema of eyelids, ankles, face, limbs and even body, or pleural effusion and ascites;

② Oliguria and anuria;

③ Slippery tongue coating;

④ Deep pulse.

□ Phlegm-dampness syndrome can be diagnosed by meeting both items of ①②, or any item of them plus any one of ③④⑤ (please select with " √ " before the numbers, multiple choices are allowed).

① Chest distress, accompanied by abdominal distension, or tight throat, or unfavorable expectoration, or nausea and vomiting with much sputum;

② Obesity;

③ Dizziness, heavy head, or heavy limbs;

④ White greasy tongue coating;

⑤ Slippery pulse.

(2) Blood stasis syndrome: □ No □ Yes

Any item of ①②③④ is met can be diagnosed (please select with " √ "before the numbers, multiple choices are allowed)

① Sharp pain on fixed position, aggravated at night;

② Numbness and pain of limbs or even hemiplegia;

 Signature of investigator:

③ Scaly dry skin, or purple lips;

④ Purple and dark tongue, or with ecchymosis, or purple and expanded sublingual veins.

The current Chinese medicine □ None □ Yes

If "Yes", please continue to fill in the medication name (multiple choices are allowed).

Note: Chinese patent medicines (such as Yishen Huashi Granule and Yishen Huazhuo Granule) with effect of tonifying Kidney and dispelling dampness should be avoided.

□ Huangkui capsule □ Cordyceps preparation □ Sanqi oral liquid

□ Shenyan Kangfu tablet □ Niaoduqing Granule □ Shenshuaining Tablet

□ Haikun Shenxi Capsule □ Niaodukang Granule □ Niaoduling Granule

□ Others: ________________

Western medicine treatment (please select the medicine)

1. Anti-hyperglycemic drugs: □ None □ Yes

□ Metformin

□ Acarbose

□ Non-sulfonylurea insulin secretagogue (Repaglinide/Nateglinide)

□ Sulfonylurea (Gliquidone/Glibenclamide/Glimepiride)

□ Thiazolidinedione (Pioglitazone/Rosiglitazone)

□ DPP-4 inhibitor (Sitagliptin phosphate/Repagliptin/Vigliptin)

□ SGLT-2 inhibitor (Empagliflozin/Dapagliflozin/Canagliflozin)

□ GLP-1 receptor agonist (Liraglutide)

□ Other oral hypoglycemic agents:

____

□ Insulin

2. Antihypertensive drugs: □ None □ Yes

□ ACEI/ARB drugs □ Calcium antagonist □ β receptor blockers

□ α receptor blockers □ Others: ___________

3. Diuretics: □ None □ Yes

□ Furosemide □ Spironolactone □ Torasemide □ Hydrochlorothiazide

□ Others: ___________

4. Lipid-lowering drugs: □ None □ Yes

□ Statins □ Fibrates □ Others: ___________

5. Uric acid lowering drugs: □ None □ Yes

□ Febuxostat □ Benzbromarone □ Allopurinol □ Sodium bicarbonate tablets

□ Others: ___________

6. Drugs for renal anemia: □ None □ Yes

□ Erythropoietin □ Iron □ Folic acid tablets

□ Others: ___________________________________

7. Correct the disorder of calcium and phosphorus metabolism: □ None □ Yes

□ Calcium carbonate □ Calcitriol □ Silam □ Lanthanum carbonate

□ Others: ___________________________________

8. Anticoagulant and anti-polymer drugs: □ None □ Yes

□ Aspirin □ Plavix □ Clopidogrel bisulfate

□ Others: ___________________________

9. Cardiovascular drugs: □ None □ Yes

□ Trimetazidine Hydrochloride Tablets (Wan Shuang Li)

□ Bisoprolol Fumarate (Kang Xin)

□ Others: _____________________________________

10. Other drugs: □ None □ Yes

If yes, there are:___________________________________

Name and signature of the investigator: ___________

Date: ___/___/____

Visit 14 (Week 50)

Note: 15 ml of venous blood sample, 10 ml of urine sample will be collected from participants at this visit. Chinese medicine syndrome of dampness assessment forms are required to complete by the guidance of investigators.

Participant profile

Questions	Status	Notes
Did the participant lost to follow-up?	□ Yes □ No	If yes, please fill in the Research Completion Form.
Did any adverse event or serious adverse event occur?	□ Yes □ No	If yes, please fill in the Adverse Event Form or Serious Adverse Event Form.
Did any composite renal endpoint events (receipt of a kidney transplant, initiation of maintenance dialysis, death from kidney failure, or a sustained low GFR and a sustained percent decline in GFR) occur?	□ Yes □ No	If yes, please complete the Endpoint Event Laboratory Record and the Composite Renal Endpoint Event Form.

Physical examination data

Heart Rate	Respiratory Rate	Blood pressure	mmHg	Weight		kg

Laboratory parameters: please paste the results of following outcomes on the "Laboratory Outcome Report (stick here) (Visit 14)"

Outcomes:

eGFR (by CKD-EPI equation)
Urinary protein creatinine ratio
Urinary albuminuria creatinine ratio
HbA1c, fasting blood-glucose
Serum creatinine, blood urea nitrogen, serum uric acid, total carbon dioxide)
ALT, AST, AST/ALT, plasma albumin and globulin)
Triglyceride, cholesterol, LDL-C, HDL-C
Serum potassium/phosphorus/calcium
White blood cell, lymphocyte, neutrophil, monocyte, red blood cell, hemoglobin)
Fecal routine and occult blood test
Electrocardiogram and urinary colour ultrasound

Diagnosis of Chinese medicine syndrome (required)

1. Primary syndrome:

Please select specific syndrome with “ √ ” (single choice):

- ☐ *qi* deficiency of Spleen and Kidney
- ☐ *yang* deficiency of Spleen and Kidney
- ☐ *qi* and *yin* deficiency
- ☐ *yin* deficiency of Liver and Kidney

 Signature of investigator:

- ☐ *yin* and *yang* deficiency
- ☐ Others ___________________

2. Secondary syndrome:

(1) Dampness syndrome: □ No □ Yes

If "Yes", please select specific syndrome with “ √ ” (single choice):

☐ Damp-turbidity syndrome can be diagnosed by meeting any two items of ①②③④ (please select with “ √ ” before the numbers, multiple choices are allowed).

① Loss of appetite, or accompanied by nausea and vomiting, or accompanied by abdominal fullness, or indifferent expression, or restlessness, or itchy skin;

② Sticky mouth and smelly urine;

③ Difficult defecation, or even constipation, accompanied by frequent urination at night, or less urination;

④ Greasy tongue coating.

☐ Damp-heat syndrome can be diagnosed if any 2 items of ①②③ are met, or if any item of ①②③ plus any item of ④⑤ (please select with “ √ ” before the numbers, multiple choices are allowed).

① Dizziness, or soreness and weakness of waist and legs, heavy limbs, or stomach distension, or abdominal fullness, or nausea, or loss of appetite;

② Sticky mouth or sweet mouth;

③ Sticky stool, or deep-yellow urine, astringent pain in urination, or more leucorrhea, or smelly leucorrhea;

④ Red tongue with yellow greasy tongue coating;

⑤ Slippery pulse, or rolling and rapid pulse.

☐ Water-dampness syndrome can be diagnosed if any item is met (please select with “ √ ” before the numbers, multiple choices are allowed).

① Edema of eyelids, ankles, face, limbs and even body, or pleural effusion and ascites;

② Oliguria and anuria;

③ Slippery tongue coating;

④ Deep pulse.

☐ Phlegm-dampness syndrome can be diagnosed by meeting both items of ①②, or any item of them plus any one of ③④⑤ (please select with " √ " before the numbers, multiple choices are allowed).

① Chest distress, accompanied by abdominal distension, or tight throat, or unfavorable expectoration, or nausea and vomiting with much sputum;

② Obesity;

③ Dizziness, heavy head, or heavy limbs;

④ White greasy tongue coating;

⑤ Slippery pulse.

(2) Blood stasis syndrome: □ No □ Yes

Any item of ①②③④ is met can be diagnosed (please select with " √ "before the numbers, multiple choices are allowed)

① Sharp pain on fixed position, aggravated at night;

② Numbness and pain of limbs or even hemiplegia;

③ Scaly dry skin, or purple lips;

④ Purple and dark tongue, or with ecchymosis, or purple and expanded sublingual veins.

The current Chinese medicine □ None □ Yes

If "Yes", please continue to fill in the medication name (multiple choices are allowed).

Note: Chinese patent medicines (such as Yishen Huashi Granule and Yishen Huazhuo Granule) with effect of tonifying Kidney and dispelling dampness should be avoided.

□ Huangkui capsule □ Cordyceps preparation □ Sanqi oral liquid

□ Shenyan Kangfu tablet □ Niaoduqing Granule □ Shenshuaining Tablet

□ Haikun Shenxi Capsule □ Niaodukang Granule □ Niaoduling Granule

□ Others: ________________

Western medicine treatment (please select the medicine)

1. Anti-hyperglycemic drugs: □ None □ Yes

□ Metformin

□ Acarbose

□ Non-sulfonylurea insulin secretagogue (Repaglinide/Nateglinide)

□ Sulfonylurea (Gliquidone/Glibenclamide/Glimepiride)

□ Thiazolidinedione (Pioglitazone/Rosiglitazone)

□ DPP-4 inhibitor (Sitagliptin phosphate/Repagliptin/Vigliptin)

□ SGLT-2 inhibitor (Empagliflozin/Dapagliflozin/Canagliflozin)

□ GLP-1 receptor agonist (Liraglutide)

□ Other oral hypoglycemic agents:

____

□ Insulin

2. Antihypertensive drugs: □ **None** □ **Yes**

□ ACEI/ARB drugs □ Calcium antagonist □ β receptor blockers

□ α receptor blockers □ Others: __________

3. Diuretics: □ **None** □ **Yes**

□ Furosemide □ Spironolactone □ Torasemide □ Hydrochlorothiazide

□ Others: __________

4. Lipid-lowering drugs: □ **None** □ **Yes**

□ Statins □ Fibrates □ Others: __________

5. Uric acid lowering drugs: □ **None** □ **Yes**

□ Febuxostat □ Benzbromarone □ Allopurinol □ Sodium bicarbonate tablets

□ Others: __________

6. Drugs for renal anemia: □ **None** □ **Yes**

□ Erythropoietin □ Iron □ Folic acid tablets

□ Others: ______________________________

7. Correct the disorder of calcium and phosphorus metabolism: □ **None** □ **Yes**

□ Calcium carbonate □ Calcitriol □ Silam □ Lanthanum carbonate

□ Others: ______________________________

8. Anticoagulant and anti-polymer drugs: □ **None** □ **Yes**

□ Aspirin □ Plavix □ Clopidogrel bisulfate

□ Others: ________________________

9. Cardiovascular drugs: □ **None** □ **Yes**

□ Trimetazidine Hydrochloride Tablets (Wan Shuang Li)

□ Bisoprolol Fumarate (Kang Xin)

□ Others: ____________________________________

10. Other drugs: □ None □ Yes
If yes, there are:__________________________________

Have you filled in the assessment forms (Week 50) of dampness syndrome of Chinese medicine? □ Yes □ No If "No" is selected, please fill in.

Whether biological specimens have been obtained?
Blood □ Obtained □ Not Obtained
Urine □ Obtained □ Not Obtained

This is the end of treatment period.
Please complete the following questionnaire.

 Signature of investigator:

Credibility of Blinding Questionnaire

For assessing the effectiveness of the blinding procedure employed in this study, please indicate which treatment you believe you have received (please √):

☐ Tangshen Qushi Formula granule treatment

☐Tangshen Qushi Formula placebo treatment

☐ Not sure

Name and signature of the investigator: ___________

Date: ___/___/____

RMIT Classification: Trusted

Laboratory Outcome Report (stick here)
(Visit 14)

Signature of investigator:

Visit 15 (Week 54)

Participant profile

Questions	Status	Notes
Did the participant lost to follow-up?	□ Yes □ No	If yes, please fill in the Research Completion Form.
Did any adverse event or serious adverse event occur?	□ Yes □ No	If yes, please fill in the Adverse Event Form or Serious Adverse Event Form.
Did any composite renal endpoint events (receipt of a kidney transplant, initiation of maintenance dialysis, death from kidney failure, or a sustained low GFR and a sustained percent decline in GFR) occur?	□ Yes □ No	If yes, please complete the Endpoint Event Laboratory Record and the Composite Renal Endpoint Event Form.

Physical examination data

Heart Rate	Respiratory Rate	Blood pressure	mmHg	Weight	kg

Diagnosis of Chinese medicine syndrome (required)

1. Primary syndrome:

Please select specific syndrome with “ √ ” (single choice):

- ☐ *qi* deficiency of Spleen and Kidney
- ☐ *yang* deficiency of Spleen and Kidney
- ☐ *qi* and *yin* deficiency
- ☐ *yin* deficiency of Liver and Kidney
- ☐ *yin* and *yang* deficiency
- ☐ Others ____________________

2. Secondary syndrome:

(1) Dampness syndrome: □ No □ Yes

If "Yes", please select specific syndrome with “ √ ” (single choice):

☐ Damp-turbidity syndrome can be diagnosed by meeting any two items of ①②③④ (please select with “ √ ” before the numbers, multiple choices are allowed).

① Loss of appetite, or accompanied by nausea and vomiting, or accompanied by abdominal fullness, or indifferent expression, or restlessness, or itchy skin;

② Sticky mouth and smelly urine;

③ Difficult defecation, or even constipation, accompanied by frequent urination at night, or less urination;

④ Greasy tongue coating.

 Signature of investigator:

□ Damp-heat syndrome can be diagnosed if any 2 items of ①②③ are met, or if any item of ①②③ plus any item of ④⑤ (please select with " √ " before the numbers, multiple choices are allowed).

① Dizziness, or soreness and weakness of waist and legs, heavy limbs, or stomach distension, or abdominal fullness, or nausea, or loss of appetite;

② Sticky mouth or sweet mouth;

③ Sticky stool, or deep-yellow urine, astringent pain in urination, or more leucorrhea, or smelly leucorrhea;

④ Red tongue with yellow greasy tongue coating;

⑤ Slippery pulse, or rolling and rapid pulse.

□ Water-dampness syndrome can be diagnosed if any item is met (please select with " √ " before the numbers, multiple choices are allowed).

① Edema of eyelids, ankles, face, limbs and even body, or pleural effusion and ascites;

② Oliguria and anuria;

③ Slippery tongue coating;

④ Deep pulse.

□ Phlegm-dampness syndrome can be diagnosed by meeting both items of ①②, or any item of them plus any one of ③④⑤ (please select with " √ " before the numbers, multiple choices are allowed).

① Chest distress, accompanied by abdominal distension, or tight throat, or unfavorable expectoration, or nausea and vomiting with much sputum;

② Obesity;

③ Dizziness, heavy head, or heavy limbs;

④ White greasy tongue coating;

⑤ Slippery pulse.

(2) Blood stasis syndrome: □ No □ Yes

Any item of ①②③④ is met can be diagnosed (please select with " √ "before the numbers, multiple choices are allowed)

① Sharp pain on fixed position, aggravated at night;

② Numbness and pain of limbs or even hemiplegia;

Signature of investigator:

③ Scaly dry skin, or purple lips;

④ Purple and dark tongue, or with ecchymosis, or purple and expanded sublingual veins.

The current Chinese medicine □ None □ Yes

If "Yes", please continue to fill in the medication name (multiple choices are allowed).

Note: Chinese patent medicines (such as Yishen Huashi Granule and Yishen Huazhuo Granule) with effect of tonifying Kidney and dispelling dampness should be avoided.

□ Huangkui capsule □ Cordyceps preparation □ Sanqi oral liquid

□ Shenyan Kangfu tablet □ Niaoduqing Granule □ Shenshuaining Tablet

□ Haikun Shenxi Capsule □ Niaodukang Granule □ Niaoduling Granule

□ Others: ________________

Western medicine treatment (please select the medicine)

1. Anti-hyperglycemic drugs: □ None □ Yes

□ Metformin

□ Acarbose

□ Non-sulfonylurea insulin secretagogue (Repaglinide/Nateglinide)

□ Sulfonylurea (Gliquidone/Glibenclamide/Glimepiride)

□ Thiazolidinedione (Pioglitazone/Rosiglitazone)

□ DPP-4 inhibitor (Sitagliptin phosphate/Repagliptin/Vigliptin)

□ SGLT-2 inhibitor (Empagliflozin/Dapagliflozin/Canagliflozin)

□ GLP-1 receptor agonist (Liraglutide)

□ Other oral hypoglycemic agents:

____

□ Insulin

2. Antihypertensive drugs: □ None □ Yes

□ ACEI/ARB drugs □ Calcium antagonist □ β receptor blockers

□ α receptor blockers □ Others: ___________

3. Diuretics: □ None □ Yes

□ Furosemide □ Spironolactone □ Torasemide □ Hydrochlorothiazide

□ Others: ___________

4. Lipid-lowering drugs: □ **None** □ **Yes**
□ Statins □ Fibrates □ Others: ___________

5. Uric acid lowering drugs: □ **None** □ **Yes**
□ Febuxostat □ Benzbromarone □ Allopurinol □ Sodium bicarbonate tablets
□ Others: ___________

6. Drugs for renal anemia: □ **None** □ **Yes**
□ Erythropoietin □ Iron □ Folic acid tablets
□ Others: __________________________________

7. Correct the disorder of calcium and phosphorus metabolism: □ **None** □ **Yes**
□ Calcium carbonate □ Calcitriol □ Silam □ Lanthanum carbonate
□ Others: __________________________________

8. Anticoagulant and anti-polymer drugs: □ **None** □ **Yes**
□ Aspirin □ Plavix □ Clopidogrel bisulfate
□ Others: __________________________

9. Cardiovascular drugs: □ **None** □ **Yes**
□ Trimetazidine Hydrochloride Tablets (Wan Shuang Li)
□ Bisoprolol Fumarate (Kang Xin)
□ Others: ____________________________________

10. Other drugs: □ **None** □ **Yes**
If yes, there are:__________________________________

Name and signature of the investigator: ___________
Date: ___/___/____

Visit 16 (Week 58)

Participant profile

Questions	Status	Notes
Did the participant lost to follow-up?	□ Yes □ No	If yes, please fill in the Research Completion Form.
Did any adverse event or serious adverse event occur?	□ Yes □ No	If yes, please fill in the Adverse Event Form or Serious Adverse Event Form.
Did any composite renal endpoint events (receipt of a kidney transplant, initiation of maintenance dialysis, death from kidney failure, or a sustained low GFR and a sustained percent decline in GFR) occur?	□ Yes □ No	If yes, please complete the Endpoint Event Laboratory Record and the Composite Renal Endpoint Event Form.

Physical examination data

Heart Rate	Respiratory Rate	Blood pressure	mmHg	Weight	kg

Diagnosis of Chinese medicine syndrome (required)

1. Primary syndrome:

Please select specific syndrome with “ √ ” (single choice):

- ☐ *qi* deficiency of Spleen and Kidney
- ☐ *yang* deficiency of Spleen and Kidney
- ☐ *qi* and *yin* deficiency
- ☐ *yin* deficiency of Liver and Kidney
- ☐ *yin* and *yang* deficiency
- ☐ Others ____________________

2. Secondary syndrome:

(1) Dampness syndrome: □ No □ Yes

If "Yes", please select specific syndrome with “ √ ” (single choice):

☐ Damp-turbidity syndrome can be diagnosed by meeting any two items of ①②③④ (please select with “ √ ” before the numbers, multiple choices are allowed).

① Loss of appetite, or accompanied by nausea and vomiting, or accompanied by abdominal fullness, or indifferent expression, or restlessness, or itchy skin;

② Sticky mouth and smelly urine;

③ Difficult defecation, or even constipation, accompanied by frequent urination at night, or less urination;

④ Greasy tongue coating.

 Signature of investigator:

☐ Damp-heat syndrome can be diagnosed if any 2 items of ①②③ are met, or if any item of ①②③ plus any item of ④⑤ (please select with " √ " before the numbers, multiple choices are allowed).

① Dizziness, or soreness and weakness of waist and legs, heavy limbs, or stomach distension, or abdominal fullness, or nausea, or loss of appetite;

② Sticky mouth or sweet mouth;

③ Sticky stool, or deep-yellow urine, astringent pain in urination, or more leucorrhea, or smelly leucorrhea;

④ Red tongue with yellow greasy tongue coating;

⑤ Slippery pulse, or rolling and rapid pulse.

☐ Water-dampness syndrome can be diagnosed if any item is met (please select with " √ " before the numbers, multiple choices are allowed).

① Edema of eyelids, ankles, face, limbs and even body, or pleural effusion and ascites;

② Oliguria and anuria;

③ Slippery tongue coating;

④ Deep pulse.

☐ Phlegm-dampness syndrome can be diagnosed by meeting both items of ①②, or any item of them plus any one of ③④⑤ (please select with " √ " before the numbers, multiple choices are allowed).

① Chest distress, accompanied by abdominal distension, or tight throat, or unfavorable expectoration, or nausea and vomiting with much sputum;

② Obesity;

③ Dizziness, heavy head, or heavy limbs;

④ White greasy tongue coating;

⑤ Slippery pulse.

(2) Blood stasis syndrome: □ No □ Yes

Any item of ①②③④ is met can be diagnosed (please select with " √ "before the numbers, multiple choices are allowed)

① Sharp pain on fixed position, aggravated at night;

② Numbness and pain of limbs or even hemiplegia;

③ Scaly dry skin, or purple lips;

④ Purple and dark tongue, or with ecchymosis, or purple and expanded sublingual veins.

The current Chinese medicine □ None □ Yes

If "Yes", please continue to fill in the medication name (multiple choices are allowed).

Note: Chinese patent medicines (such as Yishen Huashi Granule and Yishen Huazhuo Granule) with effect of tonifying Kidney and dispelling dampness should be avoided.

□ Huangkui capsule □ Cordyceps preparation □ Sanqi oral liquid

□ Shenyan Kangfu tablet □ Niaoduqing Granule □ Shenshuaining Tablet

□ Haikun Shenxi Capsule □ Niaodukang Granule □ Niaoduling Granule

□ Others: ________________

Western medicine treatment (please select the medicine)

1. Anti-hyperglycemic drugs: □ None □ Yes

□ Metformin

□ Acarbose

□ Non-sulfonylurea insulin secretagogue (Repaglinide/Nateglinide)

□ Sulfonylurea (Gliquidone/Glibenclamide/Glimepiride)

□ Thiazolidinedione (Pioglitazone/Rosiglitazone)

□ DPP-4 inhibitor (Sitagliptin phosphate/Repagliptin/Vigliptin)

□ SGLT-2 inhibitor (Empagliflozin/Dapagliflozin/Canagliflozin)

□ GLP-1 receptor agonist (Liraglutide)

□ Other oral hypoglycemic agents:

____

□ Insulin

2. Antihypertensive drugs: □ None □ Yes

□ ACEI/ARB drugs □ Calcium antagonist □ β receptor blockers

□ α receptor blockers □ Others: ___________

3. Diuretics: □ None □ Yes

□ Furosemide □ Spironolactone □ Torasemide □ Hydrochlorothiazide

□ Others: ___________

 Signature of investigator:

4. Lipid-lowering drugs: □ None □ Yes

□ Statins □ Fibrates □ Others: ___________

5. Uric acid lowering drugs: □ None □ Yes

□ Febuxostat □ Benzbromarone □ Allopurinol □ Sodium bicarbonate tablets

□ Others: ___________

6. Drugs for renal anemia: □ None □ Yes

□ Erythropoietin □ Iron □ Folic acid tablets

□ Others: ________________________________

7. Correct the disorder of calcium and phosphorus metabolism: □ None □ Yes

□ Calcium carbonate □ Calcitriol □ Silam □ Lanthanum carbonate

□ Others: ________________________________

8. Anticoagulant and anti-polymer drugs: □ None □ Yes

□ Aspirin □ Plavix □ Clopidogrel bisulfate

□ Others: ________________________

9. Cardiovascular drugs: □ None □ Yes

□ Trimetazidine Hydrochloride Tablets (Wan Shuang Li)

□ Bisoprolol Fumarate (Kang Xin)

□ Others: __________________________________

10. Other drugs: □ None □ Yes

If yes, there are:________________________________

Name and signature of the investigator: ___________

Date: ___/___/____

Visit 17 (Week 62)

Note: 15 ml of venous blood sample, 10 ml of urine sample, and a stool sample will be collected from participants at this visit. Chinese medicine syndrome of dampness assessment forms are required to complete by the guidance of investigators.

Participant profile

Questions	Status	Notes
Did the participant lost to follow-up?	□ Yes □ No	If yes, please fill in the Research Completion Form.
Did any adverse event or serious adverse event occur?	□ Yes □ No	If yes, please fill in the Adverse Event Form or Serious Adverse Event Form.
Did any composite renal endpoint events (receipt of a kidney transplant, initiation of maintenance dialysis, death from kidney failure, or a sustained low GFR and a sustained percent decline in GFR) occur?	□ Yes □ No	If yes, please complete the Endpoint Event Laboratory Record and the Composite Renal Endpoint Event Form.

Physical examination data

Heart Rate	Respiratory Rate	Blood pressure	mmHg	Weight	kg

Laboratory parameters: please paste the results of following outcomes on the "Laboratory Outcome Report (stick here) (Visit 17)"

Outcomes:

eGFR (by CKD-EPI equation)
Urinary protein creatinine ratio
Urinary albuminuria creatinine ratio
HbA1c, fasting blood-glucose
Serum creatinine, blood urea nitrogen, serum uric acid, total carbon dioxide)
ALT, AST, AST/ALT, plasma albumin and globulin)
Triglyceride, cholesterol, LDL-C, HDL-C
Serum potassium/phosphorus/calcium
White blood cell, lymphocyte, neutrophil, monocyte, red blood cell, hemoglobin)
Fecal routine and occult blood test
Electrocardiogram and urinary colour ultrasound

Diagnosis of Chinese medicine syndrome (required)

1. Primary syndrome:

Please select specific syndrome with " √ " (single choice):

- ☐ *qi* deficiency of Spleen and Kidney
- ☐ *yang* deficiency of Spleen and Kidney

 Signature of investigator:

- ☐ *qi* and *yin* deficiency
- ☐ *yin* deficiency of Liver and Kidney
- ☐ *yin* and *yang* deficiency
- ☐ Others ___________________

2. Secondary syndrome:

(1) Dampness syndrome: □ No □ Yes

If "Yes", please select specific syndrome with " √ " (single choice):

☐ Damp-turbidity syndrome can be diagnosed by meeting any two items of ①②③④ (please select with " √ " before the numbers, multiple choices are allowed).

① Loss of appetite, or accompanied by nausea and vomiting, or accompanied by abdominal fullness, or indifferent expression, or restlessness, or itchy skin;

② Sticky mouth and smelly urine;

③ Difficult defecation, or even constipation, accompanied by frequent urination at night, or less urination;

④ Greasy tongue coating.

☐ Damp-heat syndrome can be diagnosed if any 2 items of ①②③ are met, or if any item of ①②③ plus any item of ④⑤ (please select with " √ " before the numbers, multiple choices are allowed).

① Dizziness, or soreness and weakness of waist and legs, heavy limbs, or stomach distension, or abdominal fullness, or nausea, or loss of appetite;

② Sticky mouth or sweet mouth;

③ Sticky stool, or deep-yellow urine, astringent pain in urination, or more leucorrhea, or smelly leucorrhea;

④ Red tongue with yellow greasy tongue coating;

⑤ Slippery pulse, or rolling and rapid pulse.

☐ Water-dampness syndrome can be diagnosed if any item is met (please select with " √ " before the numbers, multiple choices are allowed).

① Edema of eyelids, ankles, face, limbs and even body, or pleural effusion and ascites;

② Oliguria and anuria;

③ Slippery tongue coating;

④ Deep pulse.

☐ Phlegm-dampness syndrome can be diagnosed by meeting both items of ①②, or any item of them plus any one of ③④⑤ (please select with " √ " before the numbers, multiple choices are allowed).

① Chest distress, accompanied by abdominal distension, or tight throat, or unfavorable expectoration, or nausea and vomiting with much sputum;

② Obesity;

③ Dizziness, heavy head, or heavy limbs;

④ White greasy tongue coating;

⑤ Slippery pulse.

(2) Blood stasis syndrome: □ No □ Yes

Any item of ①②③④ is met can be diagnosed (please select with " √ "before the numbers, multiple choices are allowed)

① Sharp pain on fixed position, aggravated at night;

② Numbness and pain of limbs or even hemiplegia;

③ Scaly dry skin, or purple lips;

④ Purple and dark tongue, or with ecchymosis, or purple and expanded sublingual veins.

The current Chinese medicine □ None □ Yes

If "Yes", please continue to fill in the medication name (multiple choices are allowed).

Note: Chinese patent medicines (such as Yishen Huashi Granule and Yishen Huazhuo Granule) with effect of tonifying Kidney and dispelling dampness should be avoided.

□ Huangkui capsule □ Cordyceps preparation □ Sanqi oral liquid

□ Shenyan Kangfu tablet □ Niaoduqing Granule □ Shenshuaining Tablet

□ Haikun Shenxi Capsule □ Niaodukang Granule □ Niaoduling Granule

□ Others: ________________

Western medicine treatment (please select the medicine)

1. Anti-hyperglycemic drugs: □ None □ Yes

□ Metformin

□ Acarbose

□ Non-sulfonylurea insulin secretagogue (Repaglinide/Nateglinide)

□ Sulfonylurea (Gliquidone/Glibenclamide/Glimepiride)

□ Thiazolidinedione (Pioglitazone/Rosiglitazone)

□ DPP-4 inhibitor (Sitagliptin phosphate/Repagliptin/Vigliptin)

□ SGLT-2 inhibitor (Empagliflozin/Dapagliflozin/Canagliflozin)

□ GLP-1 receptor agonist (Liraglutide)

□ Other oral hypoglycemic agents:

____

□ Insulin

2. Antihypertensive drugs: □ **None** □ **Yes**

□ ACEI/ARB drugs □ Calcium antagonist □ β receptor blockers

□ α receptor blockers □ Others: ___________

3. Diuretics: □ **None** □ **Yes**

□ Furosemide □ Spironolactone □ Torasemide □ Hydrochlorothiazide

□ Others: ___________

4. Lipid-lowering drugs: □ **None** □ **Yes**

□ Statins □ Fibrates □ Others: ___________

5. Uric acid lowering drugs: □ **None** □ **Yes**

□ Febuxostat □ Benzbromarone □ Allopurinol □ Sodium bicarbonate tablets

□ Others: ___________

6. Drugs for renal anemia: □ **None** □ **Yes**

□ Erythropoietin □ Iron □ Folic acid tablets

□ Others: ________________________________

7. Correct the disorder of calcium and phosphorus metabolism: □ **None** □ **Yes**

□ Calcium carbonate □ Calcitriol □ Silam □ Lanthanum carbonate

□ Others: ________________________________

8. Anticoagulant and anti-polymer drugs: □ **None** □ **Yes**

□ Aspirin □ Plavix □ Clopidogrel bisulfate

□ Others: _________________________

9. Cardiovascular drugs: □ **None** □ **Yes**

□ Trimetazidine Hydrochloride Tablets (Wan Shuang Li)

□ Bisoprolol Fumarate (Kang Xin)
□ Others: ______________________________________

10. Other drugs: □ **None** □ **Yes**
If yes, there are:____________________________________

Have you filled in the assessment forms (Week 62) of dampness syndrome of Chinese medicine? □ **Yes** □ **No** If "No" is selected, please fill in.

Whether biological specimens have been obtained?
Blood □ Obtained □ Not Obtained
Urine □ Obtained □ Not Obtained
Feces □ Obtained □ Not Obtained

Name and signature of the investigator: ____________
Date: ___/___/____

Laboratory Outcome Report (stick here)
(Visit 17)

Signature of investigator:

Other Laboratory Outcome Report (Stick here)
(Non-scheduled visit or test)

Signature of investigator:

RMIT Classification: Trusted

Appendix 1: Endpoint Event Laboratory Record

Laboratory examination (clinical significance judgment: 0 not checked; 1. Normal; 2. Abnormal without clinical significance; 3. Abnormal clinical significance)

Parameters	Date	Result	Unit	Clinical significance				Note
				0	1	2	3	
Blood biochemistry								
Serum creatinine			µmol/L	□	□	□	□	
eGFR (EPI equation)			ml/min	□	□	□	□	
Serum uric acid			µmol/L	□	□	□	□	
Serum potassium			mmol/L	□	□	□	□	
Carbon dioxide combining power			mmol/L	□	□	□	□	
Albumin			g/L	□	□	□	□	
Alanine aminotransferase			IU/L	□	□	□	□	
Aspartate aminotransferase			IU/L	□	□	□	□	
Blood routine test								
RBC			10^{12}/L	□	□	□	□	
Hb			g/L	□	□	□	□	

Name and signature of the investigator: ___________

Date: _______________

Laboratory Report at Dialysis Endpoint (Stick here)

Signature of investigator:

RMIT Classification: Trusted

Appendix 2: Composite Renal Endpoint Event Form

Is there a composite renal endpoint event? ☐Yes ☐No
If yes, please fill in the following table:

<table>
<tr><td colspan="2">Endpoint event</td><td>Date of occurrence</td><td>Measurements</td></tr>
<tr><td colspan="4">□ Endpoint event 1: Receipt of a kidney transplant</td></tr>
<tr><td colspan="4">□ Endpoint event 2: Initiation of maintenance dialysis
(if "yes", please complete the following details, at least select one item)</td></tr>
<tr><td rowspan="9"></td><td>(Decrease of GFR: eGFR ≤ 10 mL/min/1.73m^2(EPI equation). No change after one week of conservative medical treatment or occur twice out of three times within one month.</td><td rowspan="9">Date</td><td rowspan="9">□ Hemodialysis
□ Peritoneal dialysis
□ Mixed Hemodialysis and Peritoneal dialysis</td></tr>
<tr><td>(Hyperkalemia: serum potassium ≥ 6.5 mmol/l. No change after one week of conservative medical treatment or occur twice out of three times within one month.</td></tr>
<tr><td>Metabolic acidosis: the binding capacity of carbon dioxide was less than 13mmol / L, and no change after one week of conservative treatment in internal medicine or twice out of three times within one month</td></tr>
<tr><td>Uremic pericarditis or pleurisy</td></tr>
<tr><td>Uremic encephalopathy</td></tr>
<tr><td>Refractory hypertension</td></tr>
<tr><td>Congestive heart failure</td></tr>
<tr><td>Obvious bleeding tendency</td></tr>
<tr><td>Progressive malnutrition (including anorexia, weight loss, and decreasing serum albumin)</td></tr>
<tr><td rowspan="3"></td><td colspan="3">□ End point event 3: a sustained low GFR and a sustained percent decline in GFR</td></tr>
<tr><td>□ Sustained low GFR means GFR <15 mL/min per 1.73 m^2 sustained over at least 4 weeks.</td><td rowspan="2">Date</td><td rowspan="2">___ ___ ___ ___ / ___ ___ / ___ ___
(year/month/day)</td></tr>
<tr><td>□ Sustained percent decline in GFR means percent decline in GFR of 40% from a baseline start point sustained over at least 4 weeks.</td></tr>
<tr><td colspan="4">□ Endpoint 4: Death from kidney failure
Date of death: ___ ___ ___ ___ / ___ ___ / ___ ___ (year/month/day)</td></tr>
</table>

Name and signature of the investigator: ___________ Date: _______________

Appendix 3: Research Completion Form

Summary of research completion

Last visit (date)__________

Is the study completed? Yes, no

If not, date of discontinuation or termination____________

Please select the following main reasons for discontinuation / participant's withdrawal of the study: ☐Yes ☐No

☐ **The researchers stopped the study for the following reasons:**

☐ Serious adverse events (please fill in the registration form of adverse reactions / events)

☐ Severe comorbidities were found in the course of the study, and it is estimated that the nephrology treatment plan is no longer applicable to this patient (see the protocol for details).

Endpoint events (please fill in endpoint event registration form)

☐ **The specific reasons were as follows:**

☐ Death during the study observation period (please fill in the end event registration form)

☐ The patient asked to withdraw from the study

☐ Patients lost follow-up.

☐ Patients think the curative effect is not good.

□ **Others:**

Name and signature of the investigator: ___________

Date: _______________

RMIT Classification: Trusted

Appendix 4: Adverse Event Form

Are there any adverse events? □ Yes □ No
If so, please complete following table:

STUDY TITLE: TANGSHEN QUSHI FORMULA FOR PATIENTS WITH STAGE 2-4 DIABETIC KIDNEY DISEASE: A PILOT RANDOMISED CONTROLLED TRIAL AND QUALITATIVE STUDY

**Site Name:_________________________**<br>**Participant ID:_________________________**	**This form is cumulative and captures adverse events of a single participant throughout the study.**

Severity	Study Intervention Relationship	Action Taken Regarding Study Intervention	Outcome of AE	Expected	Serious Adverse Event (SAE)
1 = Mild<br>2 = Moderate<br>3 = Severe<br>4 = Life-Threatening	0 = Not related<br>1 = Unlikely related<br>2 = Possibly related<br>3 = Probably related<br>4 = Definitely related	0 = None<br>1 = Dose modification<br>2 = Medical Intervention<br>3 = Hospitalization<br>4 = Intervention discontinued<br>5 = Other	1 = Resolved<br>2 = Recovered with minor sequelae<br>3 = Recovered with major sequelae<br>4 = Ongoing/Continuing treatment<br>5 = Condition worsening<br>6 = Death<br>7 = Unknown	1 = Yes<br>2 = No	1 = Yes<br>2 = No<br>(if yes, complete SAE form)

At end of study only: Check this box if participant had no adverse events **None**

Adverse Event	Start Date	Stop Date	Severity	Relationship	Action Taken	Outcome of AE	Expected?	SAE?

Name and signature of the investigator: ____________ Date: ________________

Appendix 5: Serious Adverse Event (SAE) Report Form

Are there any adverse events? □ Yes □ No
If yes, please complete following table:

STUDY TITLE: TANGSHEN QUSHI FORMULA FOR PATIENTS WITH STAGE 2-4 DIABETIC KIDNEY DISEASE: A PILOT RANDOMISED CONTROLLED TRIAL AND QUALITATIVE STUDY	
**Site Name:_________________________**<br>**Pt ID:_________________________**	**Date Participant Reported/Date of Site Awareness:**<br>**____/_________/______________(day/month/year)**

SAE Event Term (Diagnosis, ex: Stroke, Myocardial Infarction).
SAE onset date: ___ ___ / ___ ___ / ___ ___ ___ ___ **(day/month/year)**
SAE stop date: ___ ___ / ___ ___ / ___ ___ ___ ___ **(day/month/year)**
Location of SAE:__
Was this an unexpected adverse event? ☐ Yes ☐ No
Diagnosis for study participation:_____
Brief description of the nature of the SAE (attach description if more space is needed): ______
Category of the SAE:
☐ Date of death ___/_____/_______
☐ Life threatening
☐ Hospitalization – initial or prolonged
☐ Disability/incapacity
☐ Congenital anomaly/birth defect
☐ Required intervention to prevent permanent impairment
☐ Other:___
Intervention type:
☐ Medication or nutritional supplement (specify):__
☐ Device (specify):__
☐ Surgery (specify):__
☐ Behavioral/lifestyle (specify):
Relationship of event to intervention:
☐ Unrelated (clearly not related to the intervention)
☐ Possible (may be related to the intervention)
☐ Definite (clearly related to the intervention)
Was study intervention discontinued due to event? ☐ Yes ☐ No
What medications or other steps were taken to treat the SAE? __
List any relevant tests, laboratory data, and history, including preexisting medical conditions: ___________
Was this event a study related endpoint?
Type of report:
☐ Initial
☐ Followup
☐ Final

Signature of principal investigator:__________________ Date:______________
