# Supplementary material for: Oral Chinese Herbal Medicine plus usual care for diabetic kidney disease: study protocol for a randomized, double-blind, placebo-controlled pilot trial
Source: Front Endocrinol (Lausanne). 2024 Feb 8;15:1334609. doi: 10.3389/fendo.2024.1334609 (PMC10881862; doi:10.3389/fendo.2024.1334609)
Supplement: Supplementary File 5 — WHO Trial Registration Dataset. [file DataSheet_5.docx]

| **Data Category** | Information |
| --- | --- |
| **Primary Registry and Trial Identifying Number** | Chinese Clinical Trial Registry (ChiCTR2200062786) |
| **Date of Registration in Primary Registry** | 18 August 2022 |
| **Secondary Identifying Numbers** | N/A |
| **Source(s) of Monetary or Material Support** | State Key Laboratory of Dampness Syndrome of Chinese Medicine, The Second Affiliated Hospital of Guangzhou University of Chinese Medicine |
| **Primary Sponsor** | The Second Affiliated Hospital of Guangzhou University of Chinese Medicine |
| **Secondary Sponsor(s)** | N/A |
| **Contact for Public Queries** | Dr Meifang Liu (PhD candidate)  Tel: +86-020-81887233-35837  E-mail: [meifangliu@gzucm.edu.cn](mailto:meifangliu@gzucm.edu.cn) |
| **Public Title** | Oral Granulated Chinese Herbal Medicine plus Usual Care for Diabetic Kidney Disease: A Study Protocol for A Randomized, Double-Blind, Placebo-Controlled Pilot Trial |
| **Scientific Title** | Tangshen Qushi Formula for People with Stage 2-4 Diabetic Kidney Disease: A Pilot Randomised Controlled Trial and Qualitative Study |
| **Countries of Recruitment** | China |
| **Health Condition(s) or Problem(s) Studied** | Diabetic kidney disease |
| **Intervention (s)** | TQF group: TQF plus usual care.  Placebo group: placebo plus usual care.  Usual care includes lifestyle management, glycaemic control, antihypertensive therapy, control of proteinuria, lipid-lowering therapy, and uric acid control. |
| **Key Inclusion and Exclusion Criteria** | **Inclusion Criteria:**   - 1) Aged 18 years and over; - 2) Diagnosed with DKD using specified diagnostic criteria (see below); - 3) 15 mL/min/1.73m^2^ ≤ eGFR < 90 mL/min/1.73m^2^ and the change of eGFR within the latest 12 weeks less than 30%; - 4) Urinary protein creatinine ratio (UPCR) ≤ 3500 mg/g; - 5) Agree to be available for the period of the study; - 6) Providing written informed consent. - American Diabetes Association recommends that DKD is diagnosed based on a clear history of diabetes and a causal relationship with changes in urinary protein and renal function after excluding other primary and secondary glomerular diseases and systemic diseases. Besides, the diagnosis of DKD should meet at least one of the following criteria in the practical guideline: - 1) Urinary albumin creatinine ratio (UACR) ≥ 30 mg/g or urinary albumin excretion rate ≥ 30 mg/24h, repeated within 3 to 6 months, and 2 out of 3 times reached or exceeded the critical value after eliminating infection and other interfering factors; - 2) Estimated GFR < 60 mL/min/1.73m^2^; - 3) The renal biopsy is consistent with DKD pathological changes.   **Exclusion Criteria:**   - 1) Received maintenance dialysis treatment or kidney transplantation; - 2) Planning to accept maintenance dialysis treatment or kidney transplantation within 2 weeks; - 3) Diagnosed with acute urinary tract infection in the last two weeks; - 4) Diagnosed with other primary chronic kidney diseases (e.g., glomerulonephritis, chronic pyelonephritis, and ischemic kidney disease); - 5) Diagnosed with advanced hepatic disease (e.g., hepatic cirrhosis, decompensated cirrhosis) or other known acute and chronic active hepatitis, cirrhosis; - 6) Diagnosed with heart failure (New York Heart Association grade III-IV (28)), acute coronary syndrome, stroke, or any other cardiovascular or cerebrovascular accident requiring revascularization surgery in the last 12 weeks, or a revascularization operation is urgently needed; - 7) Diagnosed with severe acute diabetic complications include but are not limited to ketoacidosis, lactic acidosis, hypertonic non-ketotic diabetic coma, hypoglycemic coma occurring within 24 weeks; - 8) Diagnosed with active malignant tumor disease within 5 years; - 9) Diagnosed with mental illness or language barrier and are unable or unwilling to cooperate; - 10) Diagnosed with autoimmune diseases (for example, systemic lupus erythematosus) and are receiving hormone or immunosuppressive therapy; - 11) Allergic to any medications or ingredients or intolerance to these medications or ingredients (e.g., lactose intolerance); - 12) Participating in another clinical trial; - 13) Female during pregnancy or lactation; - 14) Other conditions that the investigator judged inappropriate for the study. |
| **Study Type** | Interventional  Allocation: randomised  Intervention model: parallel assignment (2 arms)  Masking: blind for all study participants, study investigators, personnel involved in outcome measurements and statisticians.  Primary purpose: feasibility and treatment  Phase: exploratory  Allocation concealment: the randomisation numbers are delivered to both study sites via a validated web-based randomisation system Interactive Web Response System for Chinese Medicine Trials.  Sequence generation: computer generated random sequence |
| **Date of First Enrollment** | September 2022 |
| **Target Sample Size** | 60 |
| **Recruitment Status** | Ongoing |
| **Primary Outcome(s)** | Changes in estimated glomerular filtration rate (eGFR slope, time frame: 48 weeks) |
| **Key Secondary**  **Outcome(s)** | 1) Change in UACR from baseline to the end of treatment and/or to the end of follow-up;  2) Change in UPCR from baseline to the end of treatment and/or to the end of follow-up;  3) Change in kidney function parameters, including serum creatinine, blood urea nitrogen, serum uric acid, and total carbon dioxide from baseline to the end of treatment and/or to the end of follow-up;  4) Change in HbA1c, fasting blood glucose (FBG), and blood lipids (cholesterol, triglycerides, low-density lipoprotein cholesterol, and high-density lipoprotein cholesterol) from baseline to the end of treatment and/or to the end of follow-up;  5) Change in a score of Dampness Syndrome Scale of Chinese Medicine baseline to the end of treatment and/or to the end of follow-up;  6) The survival time of renal composite endpoint events. The survival time is defined as starting from baseline to the occurrence of any of the below composite endpoints: receipt of a kidney transplant, initiation of maintenance dialysis, death from kidney failure, a sustained low eGFR, and a sustained percent decline in eGFR. |
